# Targeting macrophage TFEB-14-3-3 epsilon Interface by naringenin inhibits abdominal aortic aneurysm

**DOI:** 10.1038/s41421-021-00363-1

**Published:** 2022-03-01

**Authors:** Yiting Jia, Lu Zhang, Ziyi Liu, Chenfeng Mao, Zihan Ma, Wenqiang Li, Fang Yu, Yingbao Wang, Yaqian Huang, Weizhen Zhang, Jingang Zheng, Xian Wang, Qingbo Xu, Jian Zhang, Wei Feng, Caihong Yun, Chuanju Liu, Jinpeng Sun, Yi Fu, Qinghua Cui, Wei Kong

**Affiliations:** 1 Department of Physiology and Pathophysiology, School of Basic Medical Sciences, Peking University; Key Laboratory of Molecular Cardiovascular Science, Ministry of Education, Beijing, China; 2 The Affiliated Hospital of Qingdao University, Qingdao, China; 3 Department of Cardiology, China-Japan Friendship Hospital, Beijing, China; 4 Cardiovascular Division, Kings College London BHF Centre, London, SE5 9NU UK; 5 State Key Laboratory of Oncogenes and Related Genes, Medicinal Chemistry & Bioinformatics Center, Shanghai Jiao Tong University School of Medicine, Shanghai, China; 6 National Laboratory of Biomacromolecules, CAS Center for Excellence in Biomacromolecules, Institute of Biophysics, Chinese Academy of Sciences, Beijing, China; 7 Institute of Systems Biomedicine, School of Basic Medical Sciences, Peking University Health Science Center, Beijing, China; 8 Department of Orthopedic Surgery, New York University Medical Center, New York, NY USA; 9 Department of Cell Biology, New York University School of Medicine, New York, NY USA; 10 Department of Biomedical Informatics, School of Basic Medical Sciences, Peking University, Beijing, China

**Keywords:** Macroautophagy, Transcriptional regulatory elements

## Abstract

Abdominal aortic aneurysm (AAA) is a lethal cardiovascular disease, and there is no proven drug treatment for this condition. In this study, by using the Connectivity Map (CMap) approach, we explored naringenin, a naturally occurring citrus flavonoid, as a putative agent for inhibiting AAA. We then validated the prediction with two independent mouse models of AAA, calcium phosphate (CaPO_4_)-induced C57BL/6J mice and angiotensin II-infused ApoE^−/−^ mice. Naringenin effectively blocked the formation of AAAs and the progression of established AAAs. Transcription factor EB (TFEB) is the master regulator of lysosome biogenesis. Intriguingly, the protective role of naringenin on AAA was abolished by macrophage-specific TFEB depletion in mice. Unbiased interactomics, combined with isothermal titration calorimetry (ITC) and cellular thermal shift assays (CETSAs), further revealed that naringenin is directly bound to 14-3-3 epsilon blocked the TFEB-14-3-3 epsilon interaction, and therefore promoted TFEB nuclear translocation and activation. On one hand, naringenin activated lysosome-dependent inhibition of the NLRP3 inflammasome and repressed aneurysmal inflammation. On the other hand, naringenin induced TFEB-dependent transcriptional activation of GATA3, IRF4, and STAT6 and therefore promoted reparative M2 macrophage polarization. In summary, naturally derived naringenin or macrophage TFEB activation shows promising efficacy for the treatment of AAA.

## Introduction

Abdominal aortic aneurysm (AAA) is a highly lethal disorder characterized by permanent dilatation of the abdominal aorta with an approximately 80% mortality rate upon rupture^1^. AAA is epidemiologically associated with aging, smoking, male sex, high cholesterol, etc., and is mechanistically characterized by chronic aortic wall inflammation, proteolysis of extracellular matrix proteins, and dysfunction of vascular smooth muscle cells^2^. Although progress has been made in surgical options and several pharmacological agents, including angiotensin-converting enzyme inhibitors (ACEIs), angiotensin receptor blockers (ARBs), statins, macrolides, β-blockers, tetracycline, prostaglandin E2 inhibitors, and indomethacin, have been proposed to be effective in experimental animal models, there is currently no proven pharmacological therapy that can slow aneurysm growth or reverse established aneurysms^3–7^.

Recently, Connectivity Map (CMap) has provided a data-driven and systematic approach for discovering associations among genes, chemicals, and diseases based on genome-wide expression profiling^8–11^. Principally, drugs with similar transcriptome signatures may share similar mechanisms, chemical and physiological processes, and efficacies for similar diseases^12^. Previously, doxycycline, a broad-spectrum inhibitor of matrix metalloproteinases (MMPs) and a clinically used tetracycline antibiotic, was proposed to significantly prevent aneurysm formation in animal models based on the observation that aberrant matrix turnover mediated by MMPs plays a pivotal role in the pathogenesis of AAA^13,14^. In the current study, by using CMap and doxycycline as bait, we identified naringenin as an anti-AAA compound that both prevents and reverses the progression of AAA. Mechanistically, we found that naringenin was directly bound to 14-3-3 epsilon and interrupted the 14-3-3-TFEB interaction interface, therefore activating macrophage TFEB. On one hand, naringenin stimulates TFEB-dependent macrophage lysosome biogenesis and therefore inhibits inflammasome activation. On the other hand, naringenin promotes M2 macrophage polarization and reparative responses via TFEB-dependent transcriptional activation of GATA3, IRF4, and STAT6.

## Results

### Naringenin is a candidate compound for the treatment of AAA

**Fig. 1:**
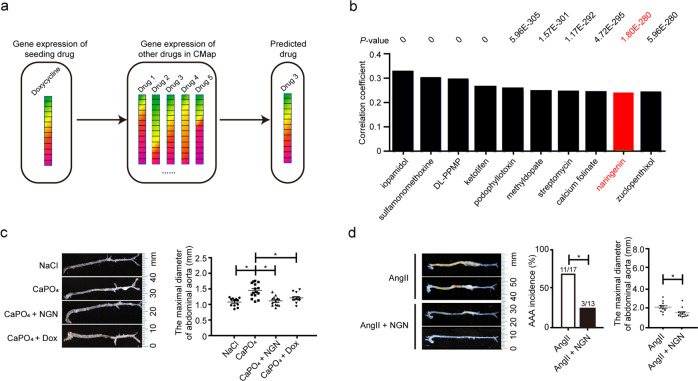
Naringenin inhibited ${\mathrm{CaPO}}_{4^-}$ and Ang II-induced AAA formation. **a** Schematic illustration of the transcriptome in response to doxycycline compared to the CMap database. The top ten drugs showing a significant correlation with doxycycline are listed in **b**. The *y*-axis represents the correlation coefficient (*R*) calculated by Spearman’s correlation analysis. The *P* value is shown on the top. **c** Representative photographs of macroscopic features of the CaPO_4_-induced aneurysms and quantification of the infrarenal aortic maximal diameter in C57BL/6J mice. NaCl, NaCl + PBS-treated plus water gavage (*n* = 12); CaPO_4_, CaCl_2_ + PBS-treated plus water gavage for 7 days (*n* = 15); CaPO_4_ + NGN, CaCl_2_ + PBS-treated plus naringenin gavage (50 mg/kg/day) (*n* = 13); CaPO_4_ + Dox, CaCl_2_ + PBS-treated plus doxycycline gavage (100 mg/kg/day) (*n* = 13). The right panel shows the quantification of the maximal diameter of the infrarenal aorta by ex vivo measurement. The data were analyzed using one-way ANOVA followed by Bonferroni test for post hoc comparison and are presented as the means ± SEM. *P < 0.05. **d** Representative photographs of macroscopic features of aneurysms in the ApoE^−/−^ mice. AngII, AngII (1000 ng/kg/min) infusion for 4 weeks plus water gavage; AngII + NGN, AngII infusion plus naringenin gavage (50 mg/kg/day). The right panel shows the incidence of AAA in the ApoE^−/−^ mice and quantification analysis of the maximal suprarenal aorta diameter by ex vivo measurement. The incidence of AAA was analyzed using the chi-square test. The maximal suprarenal aorta diameter was analyzed using an unpaired *t* test and is presented as the means ± SEM (*n* = 12 mice for each group). *P < 0.05.

We used a drug-repositioning strategy to screen all the compounds that have been integrated into CMap (Fig. 1a, Supplementary Table S1). Figure 1b lists the top ten compounds that showed the highest significance (*P* < 2.2e−16) and correlation (correlation coefficient: 0.236–0.323) with doxycycline based on their drug-responsive gene expression profiles. Among the ten compounds, some are not appropriate for clinical therapy. For example, iopamidol is a contrast material that enhances computed tomographic brain imaging^15^. Sulfamonomethoxine is a widely used antimicrobial agent added to the feed of meat-producing animals to treat infections^16^. DL-PPMP is a widely used inhibitor of glucosylceramide synthase that regulates intracellular ceramide metabolism^17^. Ketotifen is a histamine H1 receptor antagonist and used as an anti-allergic compound but not suitable for oral administration due to its acute toxicity^18^. Podophyllotoxin is used for many years for topical treatment of warts and for cancer recently due to its activity in inducing cell cycle arrest^19,20^. Some drugs, such as streptomycin and methyldopate, are clinically applicable but have frequent or severe side effects after long-term application^21,22^. Calcium folinate is a clinical act as the first-line chemotherapy regimen for colon cancer therapy^23^. Among the others, the naturally occurring grapefruit flavonoid naringenin (NGN) exhibited a significant correlation with doxycycline (*R* = 0.24, *P* < 2.2e−16) and has previously recognized lipid-lowering, anti-atherosclerotic, anti-hypertensive, and anti-inflammatory properties^24–27^. Considering the significant involvement of hypertension, atherosclerosis, and vascular inflammation in the pathogenesis of AAA^28^, we hypothesized that naringenin may inhibit the formation of AAA.

We applied two clinically relevant animal models to validate the effect of naringenin on the pathogenesis of AAA. The first model was the perivascular CaPO_4_-induced mouse model of AAA. 12-week-old male C57BL/6 J mice were randomly divided into four groups: the NaCl group (*n* = 12), the CaPO_4_ group (*n* = 15), the CaPO_4_ + NGN group that received daily oral gavage with naringenin (50 mg/kg/day) for 7 consecutive days (*n* = 13) and the CaPO_4_ + Dox group that received daily oral gavage with doxycycline (100 mg/kg/day) for 7 days (*n* = 13). There was no significant changes in body weight, blood pressure or plasma levels of total cholesterol and triglycerides within the groups (Supplementary Table S2). The maximal infrarenal aortic diameter measured ex vivo was substantially enhanced in the CaPO_4_ group but significantly decreased in both the doxycycline and naringenin groups (Fig. 1c).

Formation of AAA involves severe matrix degradation and MMP activation, aortic inflammation, and vascular smooth muscle cell (VSMC) apoptosis^29,30^. Interestingly, naringenin suppressed MMP-9 expression and activity to the same extent as the broad-spectrum MMP inhibitor doxycycline, as shown by in situ zymography, quantitative gelatin zymography, and real-time quantitative polymerase chain reaction (RT-qPCR) (Supplementary Fig. S1a–c). Interestingly, naringenin did not significantly alter the activity or mRNA level of MMP-2. Similarly, administration of naringenin significantly inhibited CD45^+^ leukocyte and Mac-3^+^ macrophage infiltration into the tunica adventitia (Supplementary Fig. S1d). Of interest, the number of apoptotic cells upon CaPO_4_ injury was reduced by naringenin but not by doxycycline (Supplementary Fig. S1e, f). Further elastin Van Gieson staining revealed dramatically decreased elastin fragmentation in the abdominal aorta of the naringenin-gavaged mice compared with that of the water-gavaged mice (Supplementary Fig. S1g). Overall, naringenin inhibited CaPO_4_-induced AAA in C57BL/6J mice.

To further validate the effect of naringenin on AAA, we next adopted an AngII-induced AAA model. Four-month-old male ApoE^−/−^ mice were infused with AngII (1000 ng/kg/min) for 1 month. The ApoE^−/−^ mice were divided into two groups by a random number table according to body weight, age, blood pressure, total cholesterol, triglycerides, low-density lipoprotein, high-density lipoprotein: the AngII group (*n* = 17) and the AngII + NGN group, which received daily naringenin gavage treatment (50 mg/kg) (*n* = 13). As previously reported, the plasma lipid profile, including total cholesterol, triglycerides, and low-density lipoprotein, was reduced in the naringenin-treated mice 1 month later^31^ (Supplementary Table S3). However, naringenin did not affect body weight, blood pressure, or high-density lipoprotein levels. Infusion of AngII caused six mice to die early due to thoracic aortic dissection (*n* = 1) and AAA rupture (*n* = 5), as well as suprarenal aneurysm formation in 6 ApoE^−/−^ mice. The combined endpoint of aneurysm development and aortic rupture occurred in 64.7% of the mice (11/17, Fig. 1d). In contrast, administration of naringenin for 28 consecutive days resulted in one case of mortality from AAA rupture caused by AngII infusion, and two mice developed aneurysms after 1 month of AngII infusion. Therefore, the total incidence of AAA formation and rupture was 23.1% (3 of 13 mice) in the naringenin group. Consistently, naringenin substantially reduced the maximum diameter of the abdominal aorta. Thus, naringenin was effective in preventing the formation of AngII-induced AAA in ApoE^−/−^ mice.

Similarly, naringenin treatment strongly repressed MMP-9 activity, leukocyte infiltration, and plasma levels of IL-6 and MCP-1, and elastin fragmentation of the abdominal aorta in the AngII-infused ApoE^−/−^ mice was also decreased by naringenin (Supplementary Fig. S1h–l).

### Naringenin reverses established AAAs

**Fig. 2:**
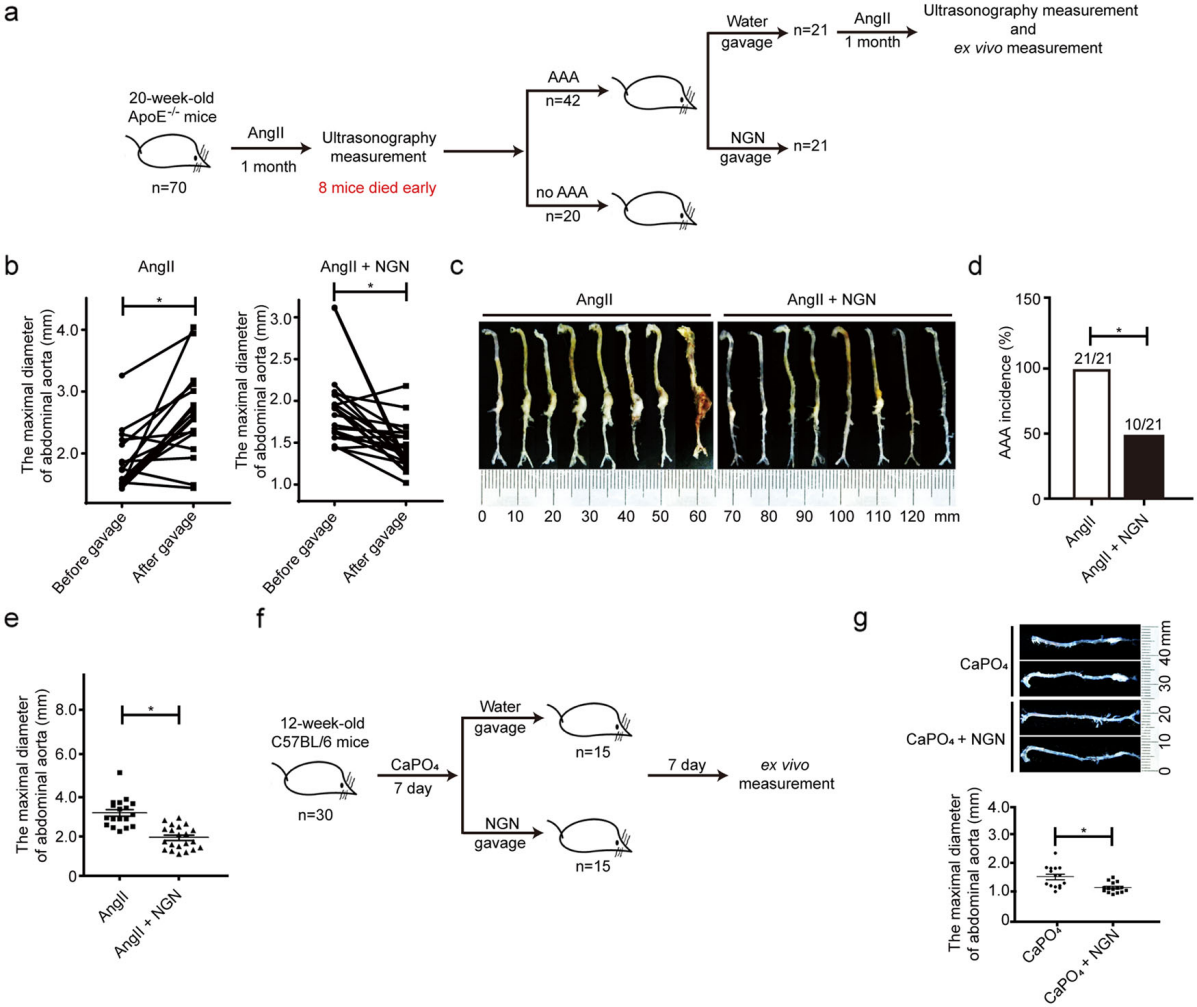
Naringenin rescued established AngII- and CaPO_4_-induced AAA. **a** Schematic illustration of the experiment used to explore the effect of naringenin on established AngII-induced AAA. **b** Quantification of the maximal suprarenal abdominal aortic diameter of the mice measured by ultrasonography with AngII-induced AAA before and after water or naringenin gavage. The data were analyzed using paired *t* test and are presented as the means ± SEM. *n* = 18–20 for each group. *P < 0.05. **c** Representative photographs of macroscopic features of aneurysmal aortas in mice with established AngII induced AAA. **d** The percentage of abdominal aortic aneurysm in established AAA of the ApoE^−/−^ mice by ex vivo measurement. %, the number of aneurysms after vehicle or naringenin gavage relative to the number of established aneurysms in the ApoE^−/−^ mice. The incidence of AAA was analyzed using the chi-square test. **e** Quantification of the maximal suprarenal abdominal aortic diameter of the naringenin- or vehicle-treated ApoE^−/−^ mice by ex vivo measurement. The maximal suprarenal aorta diameter was analyzed using an unpaired *t*-test and is presented as the means ± SEM. *P < 0.05. **f** Representative photographs of macroscopic features of aneurysmal aortas in mice with established CaPO_4_ induced AAA. **g** Representative images (left) and quantification (right) of the maximal abdominal aortic diameter of the naringenin- or vehicle-treated AAA mice by ex vivo measurement. The maximal suprarenal aorta diameter was analyzed using an unpaired *t* test and is presen*t*ed as the means ± SEM. *P < 0.05.

A recent study showed that doxycycline failed to slow the growth of established AAAs in animal models^32^. Thus, we asked whether naringenin was still effective in the treatment of established AAAs. We subcutaneously infused 1000 ng/kg/min AngII into 4-month-old male ApoE^−/−^ mice (*n* = 70). As shown in Fig. 2a, eight mice died early in the study due to thoracic aortic dissection or aneurysm rupture. Twenty-eight days later, all surviving mice underwent ultrasonography to measure the suprarenal aortic diameter; established AAA was defined as a more than 50% increase in the external diameter of the abdominal aorta. According to this criterion, 42 out of the 62 mice developed AAA. The mice were then allocated into two groups by a random number table according to body weight, age, blood pressure, maximal diameters of the abdominal aortas (naringenin: *n* = 21, 1.92 ± 0.10 mm vs vehicle: *n* = 21, 1.86 ± 0.09 mm, Fig. 2b). All 42 mice were infused with AngII (1000 ng/kg/min) for an additional 28 days. Four mice died from AAA rupture in the vehicle-treated group, and the surviving mice further developed more severe AAA. In contrast, none of the naringenin-treated mice died during the second round of the 28-day AngII infusion, and the suprarenal aortic diameter measured by ultrasonography was strongly repressed by naringenin (Fig. 2b). The plasma total cholesterol, triglycerides and low-density lipoprotein were reduced in the naringenin-treated mice (Supplementary Table S4). Ex vivo measurement of the maximum diameter of the suprarenal aorta revealed that only 10 of 21 naringenin-treated mice still had AAAs; the other mice had a major reduction in their AAAs (Fig. 2c, d). The maximum diameter of the suprarenal aorta was 3.143 ± 0.185 mm (*n* = 17) in the AngII group compared to 2.015 ± 0.127 mm (*n* = 21) in the AngII plus naringenin group (Fig. 2e). Next, 12-week-old male C57BL/6J mice (*n* = 30) were treated with CaPO_4_. When AAA developed 7 days later, the 30 mice were randomly divided into two groups. One group received daily oral gavage of naringenin (50 mg/kg/day), whereas the other group received water for another week (*n* = 15 for each). Naringenin markedly slowed the progression of AAA and reduced the diameter of the abdominal aorta compared with those of the control group (naringenin vs water 1.137 ± 0.05 vs 1.538 ± 0.11 mm, *P* < 0.05) (Fig. 2f, g).

The above data revealed that naringenin not only prevents the formation of AAAs but also delays the progression of established AAAs.

### Naringeninactivates TFEB and lysosome biogenesis in macrophages

**Fig. 3:**
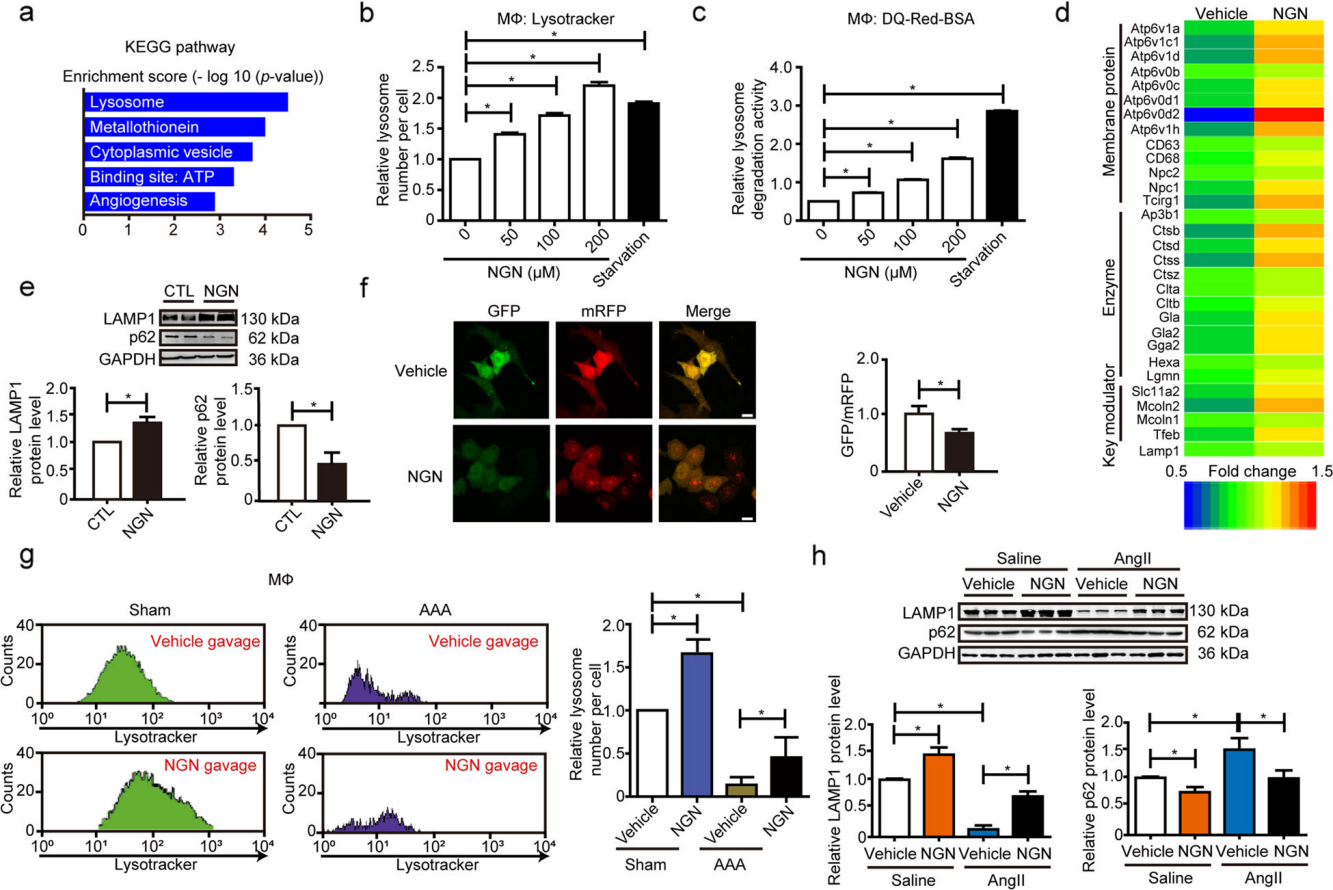
Naringenin promoted macrophage lysosome biogenesis. **a** KEGG analysis of mRNA expression that was upregulated by naringenin in the CMap database. The *x*-axis represents the −log_10_ (*P*-value). **b**, **c** Lysosomal number (**b**) and DQ-red-BSA analysis (**c**) of macrophages stimulated with naringenin (0, 50, 100, 200 µM) for 12 h or EBSS-induced starvation for 30 min. The data were analyzed using one-way ANOVA followed by Bonferroni test for post hoc comparison and are presented as the means ± SEM of six independent experiments. *P < 0.05. **d** Heatmap of genes upregulated by naringenin that are related to lysosomes in the RNA-seq analysis. **e** Western blot analysis and quantification of the protein levels of lysosome membrane protein (LAMP1) and autophagy receptor (p62) in BMDM lysates upon naringenin (200 µM, 12 h) stimulation. The data were analyzed using paired two-tailed Student’s *t* test and are presented as the means ± SEM of six independent experiments. *P < 0.05. **f** Fluorescence microscopy was used to record the autophagic flux in HeLa cells following naringenin (200 µM) treatment for 12 h after transfection with ptfLC3 plasmid. Left, representative images of fluorescent LC3 puncta. Right panel, the ratio of autophagosomes (green dots) to autolysosomes (red dots) per cell upon naringenin stimulation. Data were analyzed using unpaired two-tailed Student’s *t* test and are presen*t*ed as the means ± SEM of six independent experiments; 20–30 cells were analyzed in each independent experiment. *P < 0.05. **g** Twelve-week-old male C57BL/6J mice were periadventitially treated with CaPO_4_ and supplemented with vehicle or naringenin (50 mg/kg/day) for 7 days, and the abdominal aorta was separated and digested into single cells followed by APC-labeled F4/80 and lysotracker green staining. Flow cytometry analysis was performed to measure the fluorescence intensity of lysotracker green of each F4/80-positive macrophage (lysosome number per cell). Totally, 10–15 mice were used in each group in each experiment. The data were analyzed using two-way ANOVA followed by Bonferroni test for post hoc comparison and are presented as the means ± SEM of three independent experiments. *P < 0.05. **h** Western blot analysis and quantification of the protein levels of lysosome membrane protein (LAMP1) and autophagy receptor (p62) in the primary peritoneal macrophage cell lysates of the 12-week-old male ApoE^−/−^ mice that underwent water or AngII (1000 ng/kg/min) infusion for 4 weeks with or without naringenin gavage (50 mg/kg/day). *n* = 3 for each group. The data were analyzed using two-way ANOVA followed by Bonferroni test for post hoc comparison and are presented as the means ± SEM. *P < 0.05.

To explore the potential target of naringenin for its therapeutic effect on AAA, we analyzed naringenin-responsive genes in the CMap database. Gene ontology analysis revealed that the upregulated genes were mostly related to lysosomes, including lysosomal acid-catalyzed hydrolysis and lysosomal ion transporters (Fig. 3a, Supplementary Fig. S2a). We therefore asked whether naringenin can affect lysosome biogenesis. Naringenin dose-dependently increased lysosomal number, endocytosis, and degradation in primary peritoneal macrophages but not in endothelial cells (ECs) or VSMCs in vitro, whereas starvation substantially enhanced lysosomal number and activity in all three types of cells, as revealed by LysoTracker staining and DQ-RED-BSA assays (Fig. 3b, c, Supplementary Fig. S2b, c). By using RNA-seq analysis, we further compared the macrophage mRNA profiles upon naringenin (200 µM) or vehicle treatment. A total of 643 genes were upregulated, whereas 374 genes were downregulated in response to naringenin. DAVID Gene Ontology analysis revealed that the genes upregulated by naringenin were significantly involved in biological processes relevant to lysosomes, synaptic vesicle cycle, phagosomes, and macrophage polarization, etc. (Supplementary Fig. S2d). The upregulated lysosome genes included lysosome membrane proteins (ATP6V1A, etc.) and lysosomal enzymes (such as CTSB, CTSD, etc.), as further verified by RT-qPCR analysis (Fig. 3d, Supplementary Fig. S2e). Similarly, naringenin increased LAMP1 (a marker of lysosome number) but decreased p62 (a marker of autolysosome degradation activity) levels in macrophages, which together indicated induction of autolysosome formation and degradation (Fig. 3e). Consistent with these findings, naringenin promoted autolysosome formation, as evidenced by the mRFP-GFP tandem fluorescent-tagged LC3 (tfLC3) assay (Fig. 3f). Thus, naringenin may accelerate lysosome biogenesis and activity.

We next asked whether naringenin induced macrophage lysosome biogenesis in vivo. Naringenin administered via gavage induced a significant increase in lysosome number within F4/80^+^ macrophages but not in CD31^+^ ECs in the abdominal aorta of the CaPO_4_-induced mouse model of AAA, as evidenced by LysoTracker plus F4/80 or CD31 staining (Fig. 3g, Supplementary Fig. S2f). Reduced lysosomal number (detected by LAMP1 expression) and activity (detected by p62 degradation) were also observed in the peritoneal macrophages of the AngII-infused ApoE^−/−^ mice during the early stage (7 days), but these parameters were rescued by naringenin gavage (Fig. 3h).

**Fig. 4:**
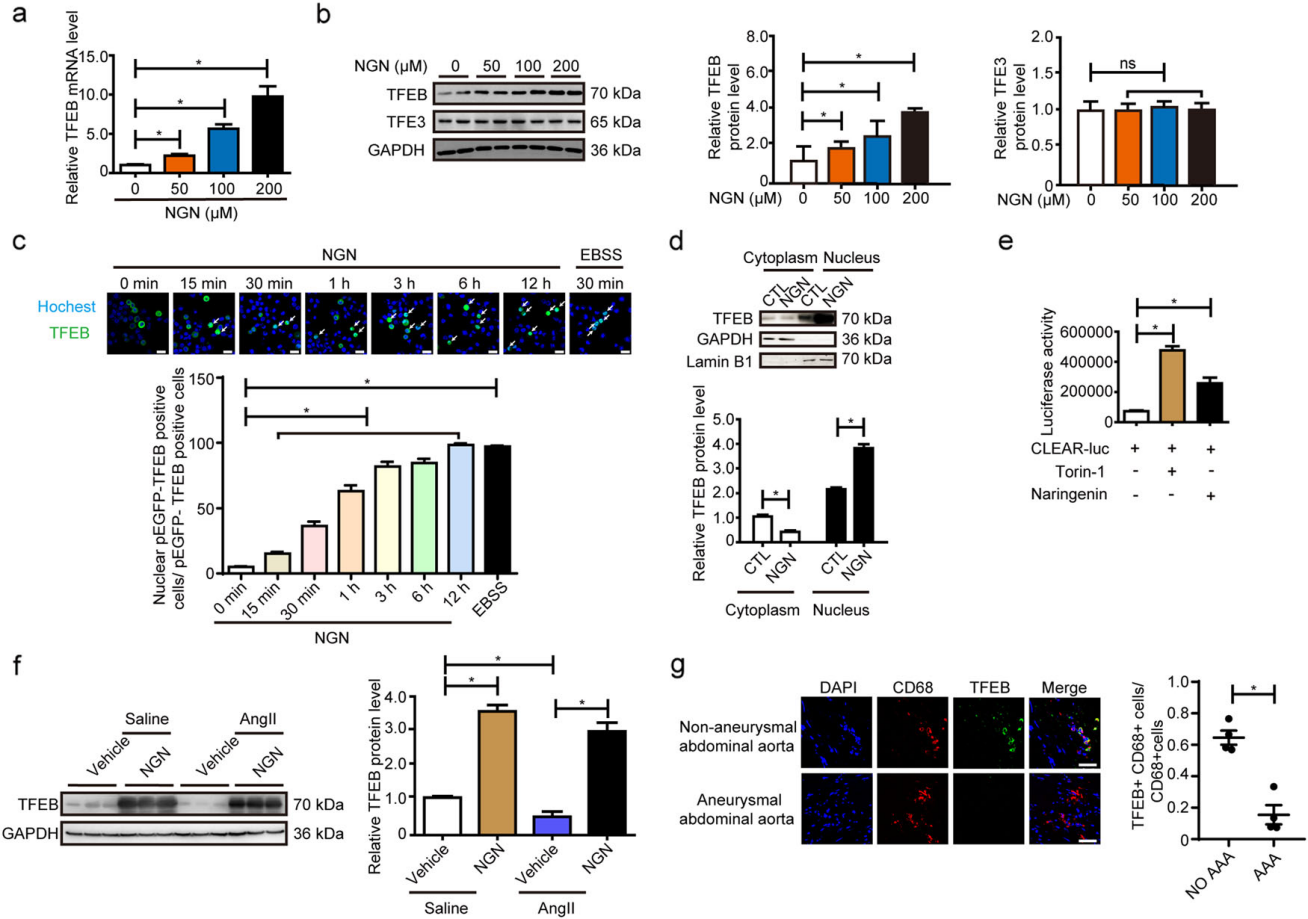
Naringenin promoted macrophage TFEB activation. **a** RT-qPCR validation of TFEB in macrophages stimulated with naringenin (0, 50, 100, 200 µM) for 12 h. The data were analyzed using one-way ANOVA followed by Bonferroni test for post hoc comparison and are presented as means ± SEM of six independent experiments. *P < 0.05. **b** Western blot analysis of TFEB and TFE3 in macrophages after naringenin (0, 50, 100, 200 µM) stimulation for 12 h. The data were analyzed using one-way ANOVA followed by Bonferroni test for post hoc comparison and are presented as the means ± SEM of six independent experiments. *P < 0.05. **c** Transfection of 293 A cells with the pEGFP-N1-TFEB plasmid was followed by vehicle or naringenin (200 µM) stimulation for 15 min to 12 h or EBSS starvation for 30 min, and then, live-cell confocal analysis was performed to study the intracellular localization of TFEB. Hochest indicated the nucleus. The white arrow indicated the cells with TFEB in the nucleus. Scale bar, 25 µm. The data were analyzed using one-way ANOVA followed by Bonferroni test for post hoc comparison and are presented as the means ± SEM of six independent experiments. *P < 0.05. **d** Nuclear-cytoplasmic separation followed by western blot analysis of vehicle- or naringenin (200 µM, 12 h)-stimulated macrophages. The data were analyzed using two-way ANOVA followed by Bonferroni test for post hoc comparison and are presented as the means ± SEM of six independent experiments. *P < 0.05. **e** Luciferase activity assay of the HEK293A cells transfected with 2× CLEAR-luc plasmid and β-galactosidase (CMV-β-gal) (the internal control) after Torin-1 (10 nM, 12 h) and naringenin stimulation (200 µM, 12 h). The data were analyzed using one-way ANOVA followed by Bonferroni test for post hoc comparison and are presented as the means ± SEM of three independent experiments. *P < 0.05. **f** Western blot analysis and quantification of the protein levels of TFEB in the primary peritoneal macrophage cell lysates of the 12-week-old male ApoE^−/−^ mice that underwent water or AngII (1000 ng/kg/min) infusion for 4 weeks with or without naringenin gavage (50 mg/kg/day). *n* = 3 for each group. The data were analyzed using two-way ANOVA followed by Bonferroni test for post hoc comparison and are presented as the means ± SEM. *P < 0.05. **g** Left, representative immunofluorescence images of TFEB (green) and CD68 (red) in human AAA arteries and control arteries. Right, quantification of the percentage of TFEB positive macrophages (CD68-positive cells). The data were analyzed using unpaired two-tailed Student’s *t* test and are presented as the means ± SEM. *n* = 4 for each group. *P < 0.05.

The regulation of lysosome biogenesis is fine-tuned by the MiTF/TFE family of transcription factors (MITF, TFEB, TFE3, and TFEC) and the suppressor Zkscan3^33,34^. Naringenin dose-dependently upregulated TFEB mRNA (Fig. 4a) without affecting the expression of TFE3, MiTF, and Zkacan3 in primary peritoneal macrophages (Supplementary Fig. S2g). TFEB but not TFE3 protein was markedly upregulated by naringenin in a dose-dependent manner (Fig. 4b). In general, the activation of TFEB relies on phosphorylation and dephosphorylation^35–38^. TFEB can be phosphorylated by mTORC1, PKCβ, Akt, etc.^37–41^. Phosphorylated TFEB locates in the cytoplasm by interacting with 14-3-3 proteins and remains inactive^42^. Under nutrient and energy stress, TFEB is dephosphorylated by calcineurin and translocate to the nucleus to activate transcription of several target genes including TFEB itself^43^. Of note, naringenin (200 µM) time-dependently promoted TFEB nuclear translocation in pEGFP-N1-TFEB-transfected HEK293A cells (Fig. 4c). Concurrently, Naringenin increased the nuclear but not the cytoplasmic level of TFEB in macrophages (Fig. 4d). However, p-PKC and p-Akt were not altered by naringenin, indicating that the effect of naringenin on TFEB activation was possibly independent of PKC and Akt (Supplementary Fig. S2h). Furthermore, mTORC1 activity, lysosome number, lysosome degradation activity, and TFEB target gene expression were not altered by naringenin in TSC Complex Subunit 1 (TSC1) or TSC Complex Subunit 2 (TSC2) deficient primary peritoneal macrophages (Supplementary Fig. S2i–p), indicating the naringenin induced TFEB activation and lysosome biogenesis is mTORC1 independent. Consistently, naringenin substantially enhanced the transcriptional activity of TFEB, as evidenced by a 2× CLEAR reporter luciferase assay. Torin-1 was applied as a positive control (Fig. 4e). Similarly, the TFEB level was strongly repressed in the peritoneal macrophages of the 7-day AngII-infused ApoE^−/−^ mice compared to the control mice, but this effect was strongly circumvented by naringenin (Fig. 4f). RT-qPCR analysis followed by flow cytometry cell sorting revealed naringenin gavage resulted in a significant increase of TFEB and TFEB target gene expression in CD11b^+^F4/80^+^ macrophages in aortas from AAA mice (Supplementary Fig. S2q). Immunofluorescence staining further revealed reduced levels of TFEB in the CD68-positive macrophages in human aneurysmal arteries compared to control arteries (Fig. 4g, Supplementary Fig. S2r). Thus, naringenin stimulates TFEB expression/activation and lysosome biogenesis in macrophages in vitro and in vivo.

### Naringenin inhibits and reverses AAA via macrophage TFEB

**Fig. 5:**
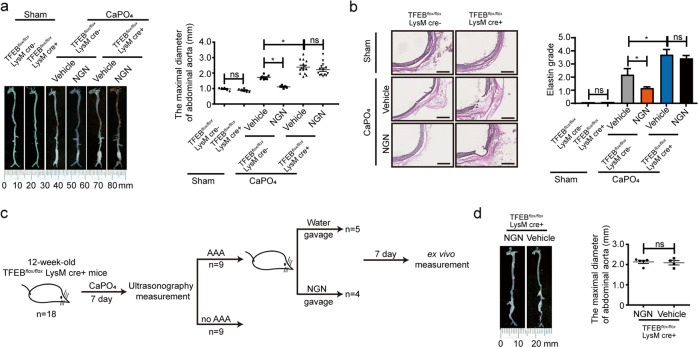
The anti-AAA effect of naringenin depended on macrophage TFEB. **a** Representative photography and maximal suprarenal abdominal aortic diameter quantification of the macroscopic features of CaPO_4_-induced aneurysms. Twelve-week-old male TFEB^*flox/flox*^ mice and TFEB^*MφKO*^ mice underwent sham operation or were periadventitially treated with CaPO_4_ followed by vehicle or naringenin (50 mg/kg/day) gavage for 7 days. *n* = 6–14 for each group. The data were analyzed using two-way ANOVA followed by Bonferroni test for post hoc comparison and are presented as the means ± SEM *P < 0.05. **b** Representative elastin Van Gieson staining and elastin grade quantification of the infrarenal abdominal aortas in panel **a**. The data were analyzed by nonparametric Kruskal–Wallis test with a dumn post hoc test. **c** Schematic illustration of the experiment used to explore the effect of naringenin on established CaPO_4_-induced AAA in TFEB^*MφKO*^ mice. **d** Left, a representative photograph of the macroscopic features of established CaPO_4_-induced aneurysms of 12-week-old TFEB^*MφKO*^ mice that supplemented with vehicle or naringenin (50 mg/kg/day) for 7 days (*n* = 4–5 for each group). Right, quantification of the maximal diameter of the infrarenal aorta by ex vivo measurement. The data were analyzed using unpaired two-tailed Student’s *t*-test and are presented as the means ± SEM. *P < 0.05.

To test whether macrophage TFEB is involved in AAA and whether naringenin inhibits AAA formation via macrophage TFEB in vivo, we generated TFEB^*MφKO*^ mice by crossbreeding TFEB floxed (TFEB^*flox/flox*^) mice with LysM-Cre mice (Supplementary Fig. S3a–c). Twelve-week-old male TFEB^*flox/flox*^ mice and TFEB^*MφKO*^ mice were grouped by a random number table according to body weight and underwent sham operation or exposed to perivascular CaPO_4_ followed by water gavage or naringenin gavage treatment. There was no significant alteration in terms of body weight, blood pressure, total cholesterol or triglycerides within the groups (Supplementary Table S5). No significant difference of infrarenal aortic diameter was observed between TFEB^*MφKO*^ mice and TFEB^*flox/flox*^ mice with the sham operation (TFEB^*flox/flox*^ mice vs TFEB^*MφKO*^: 1.01 ± 0.09 mm, *n* = 6 vs 0.91 ± 0.12 mm, *n* = 6). Meanwhile, the maximum infrarenal aortic diameter was strongly enhanced in the TFEB^*MφKO*^ mice compared to the TFEB^*flox/flox*^ mice that were exposed to perivascular CaPO_4_ (TFEB^*flox/flox*^ mice vs TFEB^*MφKO*^: 1.71 ± 0.19 mm, *n* = 6 vs 2.39 ± 0.58 mm, *n* = 13, *P* < 0.05). More severe elastin degradation was observed. In contrast, naringenin no longer inhibited aortic dilation in the TFEB^*MφKO*^ mice (water vs naringenin: 2.39 ± 0.58 mm, *n* = 13 vs 2.27 ± 0.738 mm, *n* = 14, Fig. 5a, b).

We then asked whether TFEB overexpression could mimic the protective effects of naringenin. 12-week-old male C57BL/6J mice were intravenously injected with AAV2-GFP or AAV2-TFEB (1 × 10^11^ genomic copies per mouse). The overexpression of TFEB in macrophages was confirmed by RT-qPCR (Supplementary Fig. S3d). TFEB overexpression strongly repressed CaPO_4_-induced aortic dilation (AAV2-TFEB vs AAV2-GFP: 1.97 ± 0.18 mm, *n* = 9 vs 1.55 ± 0.12 mm, *n* = 9, *P* < 0.05) (Supplementary Fig. S3e, f) without affecting body weight, blood pressure, total cholesterol, or triglycerides (Supplementary Table S6). Thus, naringenin inhibits AAA by stimulating macrophage TFEB.

Next, we verified whether naringenin could still delay the progression of established AAAs in TFEB^*MφKO*^ mice. 12-week-old male TFEB^*MφKO*^ mice (*n* = 18) were treated with CaPO_4_. When AAA developed 7 days later, the mice underwent ultrasonography to measure the suprarenal aortic diameter. According to this criterion, 9 out of the 18 mice developed AAA. The mice with AAA were then divided into two groups by a random number table by body weight, blood pressure, maximal diameter of the abdominal aorta (naringenin: *n* = 4, 1.60 ± 0.21 mm vs vehicle: *n* = 5, 1.63 ± 0.25 mm) (Fig. 5c, Supplementary Fig. S3g). Another 7 days later, ex vivo measurement revealed that naringenin failed to reduce the diameter of the abdominal aorta compared with those of the control group in TFEB^*MφKO*^ mice (naringenin vs water 2.16 ± 0.17 mm vs 2.08 ± 0.25 mm, *P* < 0.05) (Fig. 5d). Thus, the protective effects of naringenin on established AAAs were macrophage TFEB dependent.

### Naringenin directly binds to 14-3-3 epsilon and inhibits the TFEB-14-3-3 interaction

**Fig. 6:**
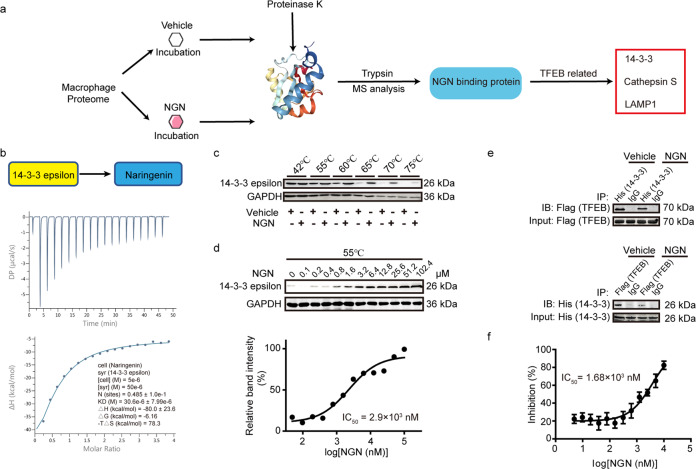
Naringenin directly bound to 14-3-3ε and inhibited its interaction with TFEB. **a** Workflow of the naringenin-interactome approach. **b** Isothermal titration plot of naringenin (in cells) with 14-3-3 (in syringes). The inset shows a graphic representation of the different thermodynamic parameters analyzed. The solid line represents the best nonlinear least-squares fit to a single binding site model. **c**, **d** CETSAs of 14-3-3ε in the absence and presence of naringenin at different temperatures (**c**) and different doses (**d**), the results were evaluated by western blots. The experiments were performed in macrophages on three independent occasions. **e** Co-IP analysis of 14-3-3ε and TFEB in the presence or absence of naringenin in 293A cells transfected with Flag-TFEB plasmid and His-14-3-3ε plasmid. Upper, the lysates were immunoprecipitated with anti-His antibody, and the precipitates were analyzed by immunoblotting with anti-Flag antibody. Lower, the lysates were immunoprecipitated with anti-Flag antibody, and the precipitates were analyzed by immunoblotting with anti-His antibody. **f** Results of the competitive binding assay for naringenin of the p-TFEB peptide (LVGVTSSpSCPADLTQ) and 14-3-3ε. The probe concentration was 10 nM, and the concentration of 14-3-3ε was 10 nM. Data are presented as the means ± SEM of three independent experiments.

To further understand the mechanism of naringenin in macrophage TFEB activation, we conducted an analysis of the naringenin-protein interactome to investigate the potential binding targets of naringenin in macrophages^44^. One-hundred ninety-nine putative naringenin-binding proteins were identified. Among the top 10 putative targets (Supplementary Table S7), 14-3-3 epsilon, a subtype of 14-3-3 proteins, has been reported to interact with phosphorylated TFEB to retain its cytoplasmic localization and inactivation (Fig. 6a). Naringenin bound to purified 14-3-3ε with a relatively high affinity as revealed by isothermal titration calorimetry (ITC), the dissociation constant (*K*_d_) value was 30.6 ± 7.99 μM (Fig. 6b). Further cellular thermal shift assay (CETSA) revealed that macrophage lysates treated with naringenin exhibited an induction in melting temperature of 14-3-3ε, from 70 to 75 °C (Fig. 6c), suggesting an interaction of naringenin and 14-3-3ε. This interaction was additionally verified by isothermal dose-response fingerprint CETSA (ITDRF-CETSA) study. Naringenin dose-dependently (from 0 to 102.4 μM) bound to and significantly stabilized the 14-3-3ε protein (IC_50_: 2.9 μM, Fig. 6d). Thus, naringenin is a direct binding partner of macrophage 14-3-3ε.

We then asked whether naringenin competitively inhibits the 14-3-3ε-TFEB interaction to activate TFEB. Indeed, naringenin significantly inhibited the interaction of TFEB and 14-3-3ε as evidenced by co-immunoprecipitation assays (Fig. 6e). In contrast, naringenin did not affect the binding of 14-3-3ε to FOXO4, a known binding partner of 14-3-3ε^45,46^ (Supplementary Fig. S4a).

The 14-3-3 proteins are phospho-regulatory proteins that can specifically recognize phosphorylated serine/threonine in the conventional motif ‘RSXpS/pTXP’ and form a hydrophilic loop. Nevertheless, the binding of p-TFEB and 14-3-3 proteins was recently described as a novel nonconventional mode by forming a hydrophobic loop^47,48^. We then asked whether naringenin was specifically occupied the hydrophobic interface between p-TFEB and 14-3-3. The miss-digested peptide of 14-3-3ε (from E162 to R171) in our naringenin-macrophage protein interactome was identical to the reported interface fragment (Supplementary Fig. S4b). We synthesized the phosphorylated TFEB peptide (p-TFEB peptide, LVGVTSSpSCPADLTQ) that mediated the 14-3-3ε interaction^47,48^ and the control phosphor-peptide (p-control peptide, MARSHpSYPAKKK) with the C-terminal FITC tag (Supplementary Fig. S4c). Purified 14-3-3ε exhibited fine binding curves to both peptides with equilibrium dissociation constants (*K*_d_) of 9.68 nM (p-TFEB peptide) and 2.4 nM (p-control peptide). Naringenin dose-dependently blocked the p-TFEB peptide-14-3-3ε interaction with an IC_50_ of 1.68 µM but failed to block the p-control peptide-14-3-3ε interaction (Fig. 6f, Supplementary Fig. S4d), suggesting naringenin specifically inhibits the 14-3-3ε-p-TFEB interaction.

Next, 14-3-3ε deficiency in macrophages resulted in dramatically TFEB translocation as well as induction of lysosome biogenesis (Supplementary Fig. S4e–h), suggesting 14-3-3ε deficiency could mimic the effect of naringenin on TFEB activation.

### Naringenin ameliorates AAA via TFEB-dependent inhibition of the NLRP3 inflammasome

**Fig. 7:**
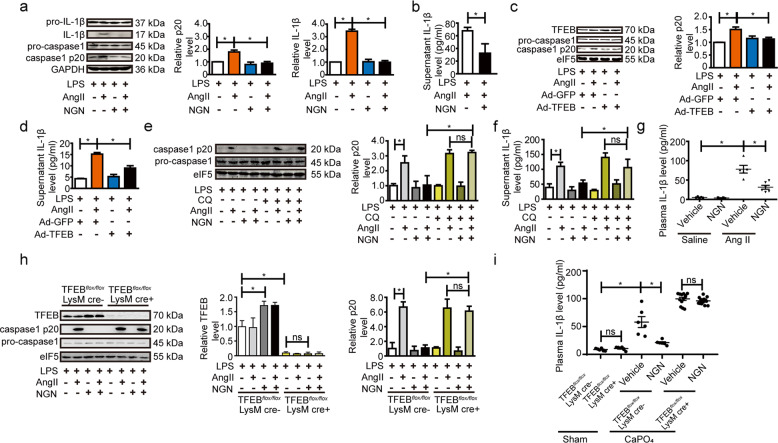
Naringenin attenuated the progression of AAA via inhibition of the NLRP3 inflammasome in a lysosome-dependent manner. **a**–**f** Western blot analysis of the protein levels of caspase-1 and IL-1β in cell lysates (**a**) and ELISAs of the supernatant (**b**) from the vehicle- or naringenin (200 µM, 12 h)-stimulated primary peritoneal macrophages followed by LPS (1 ng/ml, 3 h) and AngII (1 µM, 30 min) treatment. The data were analyzed using two-way ANOVA followed by Bonferroni test for post hoc comparison and are presented as the means ± SEM of six independent experiments. *P < 0.05. Western blot analysis and quantification of the protein level of caspase-1 in cell lysates (**c**) and ELISAs of the supernatant level of IL-1β (**d**) from the Ad-GFP (5 MOI) or Ad-TFEB (5 MOI)-infected primary peritoneal macrophages followed by LPS (1 ng/ml, 3 h) and AngII (1 µM, 30 mis) treatment. The data were analyzed using two-way ANOVA followed by Bonferroni test for post hoc comparison and are presented as the means ± SEM of six independent experiments. *P < 0.05. Western blot analysis and quantification of the protein level of caspase-1 in the cell lysates (**e**) and ELISAs of the supernatant level IL-1β (**f**) from vehicle- or naringenin (200 µM, 12 h)-stimulated primary peritoneal macrophages followed by LPS (1 ng/ml, 3 h) and AngII (1 µM, 30 min) treatment in the presence or absence of CQ (1 µM). The data were analyzed using two-way ANOVA followed by Bonferroni test for post hoc comparison and are presented as the means ± SEM of six independent experiments. *P < 0.05. **g** ELISAs of the plasma IL-1β level in the 4-month-old male ApoE^−/−^ mice gavaged with water or naringenin (50 mg/kg/day) and infused with AngII (1000 ng/kg/min, 4 weeks). *n* = 6–8 for each group. The data were analyzed using two-way ANOVA followed by Bonferroni test for post hoc comparison and are presented as the means ± SEM. **h** Western blot analysis of the protein levels of caspase-1 in cell lysates from the vehicle- or naringenin (200 µM, 12 h)-stimulated primary peritoneal macrophages isolated from 8-week-old male TFEB^*flox/flox*^ mice and TFEB^*MφKO*^ mice followed by LPS (1 ng/ml, 3 h) and AngII (1 µM, 30 min) treatment. The data were analyzed using two-way ANOVA followed by Bonferroni test for post hoc comparison and are presented as the means ± SEM of six independent experiments. *P < 0.05. **i** ELISAs of the plasma IL*-*1β level in 12-week-old male TFEB^*flox/flox*^ mice and TFEB^*MφKO*^ mice underwent sham operation or were periadventitially treated with CaPO_4_ followed by vehicle or naringenin (50 mg/kg/day) gavage for 7 days. *n* = 6–14 for each group. The data were analyzed using two-way ANOVA followed by Bonferroni test for post hoc comparison and are presented as the means ± SEM, *P < 0.05.

Lysosomes are at the epicenter of inflammasome activation in macrophages, whereas inflammasome activation is implicated in the formation of AAA, as evidenced by results from NLRP3, caspase-1, ASC and interleukin 1β (IL-1β) knockout mice^49,50^. Activation of NLRP3 inflammasome depends on 2 signals: a priming signal such as LPS to induce the expression of NLRP3 and pro-IL-1β, and a second signal such as ATP to trigger the activation of caspase-1 as well as the secretion of IL-1β^51,52^. Recently, AngII was shown to be an NLRP3 inflammasome activator during the pathogenesis of AAAs^49,50^. Indeed, naringenin stimulation or TFEB overexpression inhibited AngII-induced macrophage NLRP3 inflammasome activation, as revealed by repressed cleaved caspase-1 (p20) and IL-1β release (Fig. 7a–d). Of interest, the application of chloroquine to inhibit lysosome acidification abolished the inhibitory effect of naringenin on caspase-1 activation and IL-1β secretion (Fig. 7e, f).

To test whether naringenin or TFEB inhibits AAA formation by suppressing NLRP3 inflammasome activation in vivo, we first measured the plasma level of IL-1β in the AngII-induced mouse model of AAA. AngII infusion significantly increased the plasma level of IL-1β (saline + vehicle vs AngII + vehicle, 6.51 ± 1.62 vs 75.68 ± 10.17 pg/ml, *P* < 0.05). Naringenin administration strongly reduced the plasma level of IL-1β in the AngII-induced ApoE^−/−^ mouse model (AngII + vehicle vs AngII + NGN, 75.68 ± 10.17 vs 28.16 ± 6.75 pg/ml, *P* < 0.05) (Fig. 7g). Next, we investigated the effects of naringenin on AngII induced NLRP3 inflammasome activation, in the presence or absence of TFEB. TFEB knockout remarkably blocked the effects of naringenin, as evidenced by western blotting analysis of mature caspase-1 in vitro (Fig. 7h) and ELISA analysis of plasma IL-1β in vivo (Fig. 7i).

Together, naringenin and TFEB attenuate the formation of AAA at least partially through inhibition of the NLRP3 inflammasome in a lysosome-dependent manner.

### Naringenin promotes TFEB-dependent macrophage M2 polarization

**Fig. 8:**
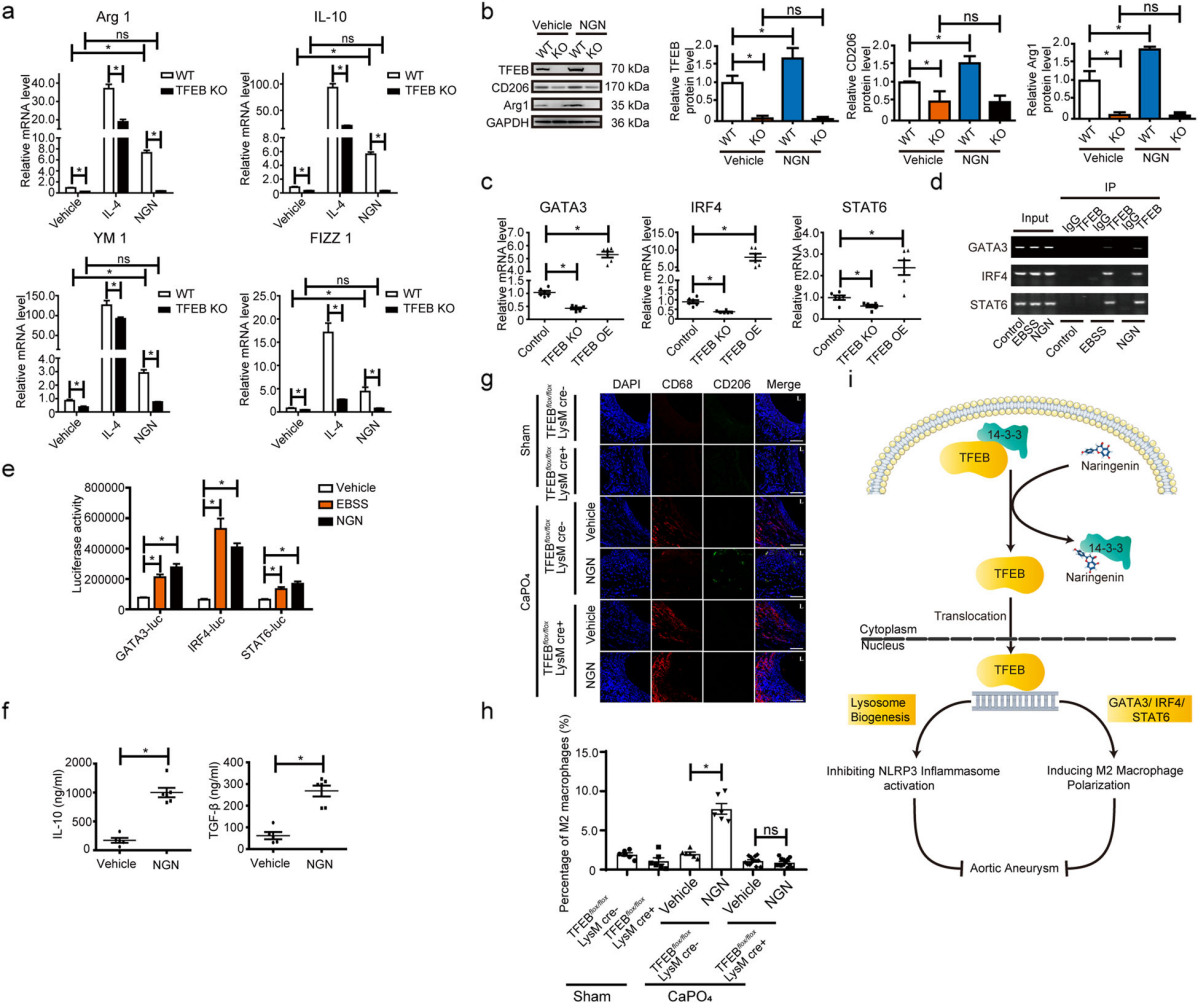
Naringenin promoted macrophage M2 polarization via activation of TFEB. **a** RT-qPCR validation of Arg1, IL-10, YM1, and FIZZ1 in the wild-type (WT) or TFEB knockout (TFEB KO) macrophages treated with vehicle, IL-4 (10 ng/ml, 12 h) or naringenin (200 µM, 12 h). The data were analyzed using two-way ANOVA followed by Bonferroni test for post hoc comparison and are presented as the means ± SEM of six independent experiments. *P < 0.05. **b** Western blot analysis and quantification of the protein level of the macrophage M2 polarization markers Arg1 and CD206 in the lysates of the wild-type (WT) or TFEB knockout (TFEB KO) macrophages treated with vehicle or naringenin (200 µM, 12 h). The data were analyzed using two-way ANOVA followed by Bonferroni test for post hoc comparison and are presented as the means ± SEM of six independent experiments. *P < 0.05. **c** RT-qPCR validation of GATA3, IRF4, and STAT6 in the wild-type (WT, TFEB^*flox/flox*^ macrophages infected with Ad-GFP), TFEB knockout (TFEB KO, TFEB^*flox/flox*^ macrophages infected with Ad-Cre), and TFEB-overexpressing (TFEB OE, TFEB^*flox/flox*^ macrophages infected with Ad-TFEB) macrophages. The data were analyzed using one-way ANOVA and are presented as the means ± SEM of six independent experiments. *P < 0.05. **d** ChIP-qPCR analyses of the interactions between TFEB and promoters of three M2 macrophage polarization-driven genes. Formaldehyde cross-linked genomic DNA from the naringenin (200 µM, 12 h)- or EBSS (30 min)-treated BMDMs was reduced to fragment sizes of 180 bp to 330 bp via enzymatic digestion for immunoprecipitation. **e** Luciferase activity assays of the HEK293A cells transfected with the pGL3-GATA3-Luc plasmid, pGL3-IRF4-Luc plasmid, pGL3-STAT6-Luc plasmid, and β-galactosidase (CMV-β-gal) (the internal control) that underwent EBSS-induced starvation (30 min) or naringenin treatment (200 µM, 12 h). The data were analyzed using two-way ANOVA followed by Bonferroni test for post hoc comparison and are presented as the means ± SEM of six independent experiments. *P < 0.05. **f** ELISAs of the vehicle- or naringenin (200 µM, 12 h)-treated BMDM supernatants containing IL-10 (left) and TGF-β (right). The data were analyzed using unpaired *t* tests and are presented as the means ± SEM of six independent experiments. **g** Representative images of the immunofluorescence staining of CD68 (Red) and CD206 (green) in the aortas of 12-week-old male TFEB^*flox/flox*^ mice and TFEB^*MφKO*^ mice underwent sham operation or were periadventitially treated with CaPO_4_ followed by vehicle or naringenin (50 mg/kg/day) gavage for 7 days. L indicates lumen. **h** Quantification of the percentage of M2 macrophages (CD68^+^CD206^+^ cells) in the aortas of 12-week-old male TFEB^*flox/flox*^ mice and TFEB^*MφKO*^ mice underwent sham operation or were periadventitially treated with CaPO_4_ followed by vehicle or naringenin (50 mg/kg/day) gavage for 7 days. The data were analyzed using two-way ANOVA followed by Bonferroni test for post hoc comparison and are presented as the means ± SEM, *P < 0.05. *n* = 6–14 for each group. **i** Schematic illustration of the naringenin and TFEB act**i**vation in abdominal aortic aneurysm prevention and regression.

Alternatively, activated (M2) macrophages are known to be related to the resolution of inflammation, tissue repair, and remodeling^53,54^. Dysregulation of macrophage polarization and the balance between classically activated (M1) macrophages and alternatively activated M2 macrophages are involved in the pathogenesis of AAA^55,56^. We next investigated whether TFEB deficiency affects macrophage differentiation. No significant differences were observed regarding the numbers of myeloid cells (Ly6C^+^/CD45^+^ in bone marrow) and monocytes (Ly6C^high^ in CD45^+^CD11b^+^Ly6G^−^ blood cells) between the TFEB^*flox/flox*^ mice and the TFEB^*MφKO*^ mice (Supplementary Fig. S5a, b). Next, we cultured bone marrow-derived macrophages (BMDMs) isolated from the TFEB^*flox/flox*^ mice and the TFEB^*MφKO*^ mice and then treated them with IL-4 (10 ng/ml) or LPS (10 ng/ml). RT-qPCR analysis revealed that TFEB knockout macrophages exhibited an abated induction of M2 macrophage markers such as Arg1, IL-10, Ym1, and Fizz1 by IL-4 stimulation, suggesting that TFEB may contribute to IL-4-promoted M2 polarization (Fig. 8a). In contrast, LPS-induced expression of M1 macrophage markers, such as IL-1β, TNF-α, iNOS, and MCP-1, was not altered in the TFEB knockout macrophages or the TFEB-overexpressing macrophages (Supplementary Fig. S5c). We next asked whether naringenin could promote macrophage M2 polarization through TFEB. Both RT-qPCR and Western blotting analysis revealed that the naringenin-treated macrophages exhibited a dramatic induction of M2 markers (Arg1, CD206, etc.), which could be blocked by genetic knockout of TFEB (Fig. 8a, b). The above data indicated that naringenin-mediated promotion of macrophage M2 polarization was TFEB dependent.

The transcription factors IRF9, IRF5, STAT1, KLF6, and RBP-J are known to be responsible for M1 polarization, whereas GATA3, IRF4, STAT6, KLF4, KLF2, etc. are crucial for M2 polarization^57–59^. In TFEB-deficient (KO) or TFEB-overexpressing (OE) macrophages, the expression of the M2 transcription factors GATA3, IRF4, and STAT6 were greatly affected by TFEB, whereas the expression of M1 transcription factors (IRF9, IRF5, STAT1, etc.) were not altered (Fig. 8c, Supplementary Fig. S5d–f). Further ChIP-PCR revealed the direct binding of TFEB to the mouse GATA3, IRF4, and STAT6 promoters, which could be induced by TFEB activation stimulus such as EBSS or naringenin (Fig. 8d). In accordance, activation of TFEB by naringenin or EBSS resulted in a significant induction of GATA3, IRF4, and STAT6 promoter-driven luciferase activity (Fig. 8e). Thus, TFEB is a novel transcriptional regulator of macrophage M2 polarization.

M2 macrophages are involved in the maintenance of tissue homeostasis partly by secreting several pro-repairing cytokines, such as IL-10 and TGF-β^60–63^. Previous reports revealed that both IL-10 and TGF-β could promote VSMC proliferation and elastin expression, thus counteracting aneurysm progression^60,64^. As shown in Fig. 8f, naringenin indeed stimulated macrophages to secrete greater amounts of IL-10 (vehicle vs NGN, 203.1 ± 13.2 pg/ml vs 989.6 ± 22.5 pg/ml) and TGF-β (vehicle vs NGN, 68.7 ± 16.7 pg/ml vs 276.8 ± 21.3 pg/ml).

Next, we performed CD68 (macrophage marker) and CD206 (M2 macrophage marker) immunostaining to address the participation of naringenin-promoted TFEB activation and M2 polarization in the treatment of AAAs in vivo. As shown in Fig. 8g, the quantity of CD68^+^ macrophage was dramatically increased in CaPO_4_ induced AAA aortas of TFEB^*MφKO*^ mice, compared with that of TFEB^*flox/flox*^ mice. Consistent of the data shown in Fig. 5a, naringenin gavage resulted in a reduction of CD68^+^ macrophages in AAA aortas of TFEB^*MφKO*^ mice, but not in AAA aortas of TFEB^*MφKO*^ mice. Intriguingly, naringenin gavage increased the percentage of CD206^+^ CD68^+^ macrophage (M2 macrophages) in AAA aortas of TFEB^*flox/flox*^ mice but not in AAA aortas of TFEB^*MφKO*^ mice (Fig. 8h). Taken together, naringenin, via inducing TFEB activation, promotes M2 polarization of macrophages both in vitro and in vivo.

## Discussion

Our current study identified naringenin as a novel anti-aneurysm drug candidate that suppresses the formation and promotes the regression of AAA. Intriguingly, our study revealed the potential importance of TFEB-dependent macrophage lysosome biogenesis and M2 macrophage polarization during naringenin suppression in AAA (Fig. 8i). Our data also highlight the therapeutic potential of interfering with 14-3-3 epsilon and TFEB binding for macrophage TFEB activation in related vascular inflammation.

AAA is highly lethal with no proven effective pharmacological intervention. Herein, we found that naringenin not only inhibits the pathogenesis of AAA but also prevents the progression of AAA. Currently, although several drugs including doxycycline, ACEI, statin et al. have been proposed to prevent AAA, there is no proven drugs yet for pharmacological intervention of AAA. There is also a lack of any drug known to reverse AAA. Naringenin is the most abundant naturally occurring grapefruit flavonoid, and its cardioprotective effect has been recently recognized^65,66^. Epidemiological studies have shown that greater flavonoid intake from fruits and vegetables is associated with decreased risk of developing cardiovascular disease^67,68^. Intriguingly, a recent prospective cohort study indicated an inverse association between consumption of fruit and the risk of AAA and a particularly more pronounced association with AAA rupture^69–71^. We herein provide a potential explanation of the protective role of fruit in the pathogenesis of AAA. The dose of naringenin we used in the mouse study (50 mg/kg/day) is equivalent to approximately 4.05 mg/kg/day in humans. A previous study estimated the concentration of naringenin glycosides in various brands of grapefruit juices to be between 17 and 76 mg/100 ml^72^. Therefore, the concentration of naringenin indicated for the prevention and treatment of AAA is achievable by daily fruit consumption. Uncovering naringenin as a potential candidate drug may shed light on the clinical therapy of patients with AAA.

Naringenin-mediated inhibition of the formation and progression of AAA may involve pleiotropic effects. Previous animal and in vitro studies have shown that naringenin exhibits antioxidant and anti-inflammatory effects^73–76^. A recent study revealed that naringenin promotes the intracellular degradation of pro-inflammatory cytokines of macrophages which is consistent with our results^77^. The inhibition of MMP-9 activation by naringenin in both animal models fit into the prediction that was made based on the CMap signature similarities between naringenin and doxycycline. However, doxycycline showed no effect on established AAA in mice^32^. Moreover, there have been conflicting reports on the effects of doxycycline on the progression of AAA in patients^78,79^. Furthermore, it has been reported that long-term administration of doxycycline caused minor but frequent side effects in patients^80,81^. However, naringenin exhibits no risk for safety issues in a recent randomized, controlled, single-ascending-dose clinical trial^82^. Additionally, previous studies have suggested that naringenin can improve dyslipidemia and apolipoprotein B overproduction^26,83^. The lipid-lowering effects of naringenin may partially account for its anti-aneurysm effect on ApoE^−/−^ mice. However, the CaPO_4_-induced mouse model of AAA does not mimic hyperlipidemia and atherosclerosis. Thus, other mechanisms beyond the MMP inhibition and lipid-lowering effects of naringenin are responsible for the protection of naringenin on AAA.

In the current study, we found that naringenin exerted AAA protection via macrophage TFEB. Naringenin replenished TFEB-dependent macrophage lysosomal function and promoted TFEB-dependent reparative M2 macrophage polarization. Macrophages are recognized as a central player in the formation and progression of AAA involving the promotion of ECM remodeling, inflammation, and oxidative stress^55^. Lysosomal dysfunction has been recognized in neurodegenerative disease, metabolic disorder, and cancer^84,85^. However, we know nothing about macrophage lysosomes during the pathogenesis of AAA. Herein, we found a dramatic decrease in macrophage lysosome biogenesis in both CaPO_4_-elicited and AngII-infused ApoE^−/−^ mouse models and naringenin stimulated TFEB-dependent macrophage lysosome biogenesis to inhibit AAA. Our study highlights the importance of targeting macrophage lysosomes for fighting aneurysmal diseases. Our finding that naringenin or TFEB inhibited NLRP3 inflammasome activation and reduced IL-1β secretion during AAA is consistent with previous reports that depletion of the NLRP3, caspase-1, and IL-1β inflammasomes protect against aneurysm formation in mice^86^. Although not fully understood, macrophage TFEB inhibition of inflammasomes and IL-1β secretion were also observed in previous studies within atherosclerotic and post-myocardial infarction mice models^87^. An explanation is TFEB mediated autophagy-lysosome pathway is responsible for the inhibition of inflammasomes since TFEB is the master regulator of autophagy–lysosome pathway^88–91^. Interestingly, although the gene expression profiles of naringenin and doxycycline in the Cmap database are of high similarity, the lysosome-related genes were not altered by doxycycline in the Cmap database. Besides, we observed no induction effects of doxycycline on macrophage lysosome biogenesis (Data not shown). This may explain why doxycycline showed promising in the AAA mouse model but not in clinical trials^73^. Moreover, an imbalance between proinflammatory macrophages (M1) and repairing potential macrophages (M2) is recently recognized during the pathogenesis of AAA^92,93^. Targeting CD31 to direct macrophage polarization exhibited potential beneficial effects during the formation and progression of AAA^94^. IL-10 and TGF-β, two key cytokines secreted by M2 macrophages, were reported to inhibit aneurysms^60,95^. Herein, we found that TFEB activation or naringenin stimulation promoted M2 polarization. Mechanistically, we revealed that TFEB was directly bound to the promoters of the M2 transcription factors GATA3, IRF4, and STAT6 and subsequently enhanced IL-10 and TGF-β levels to activate the reparative response. Our data highlight TFEB as a novel regulator of macrophage M2 polarization. Of note, a recent study reported VSMC specific depletion of TFEB inhibits AAA formation through inhibiting VSMC apoptosis^96^, without testing its role during AAA regression. However, we observed no effects of naringenin on lysosome biogenesis as well as lysosome degradation activity in VSMCs (Supplementary Fig. S2b, c). Meanwhile, the naringenin-mediated TFEB-dependent macrophage M2 polarization may contribute to AAA regression, since M2 macrophages are known to regulate tissue repair and remodeling^97^.

Ectopic overexpression of TFEB has been experimentally proven to be beneficial to induce the intracellular clearance of pathogenic factors in murine models of Parkinson’s disease, Alzheimer’s disease, atherosclerosis, and myocardial infarction^87,98–102^. However, how to pharmacologically activate TFEB to protect against cardiovascular diseases remains largely unknown. In general, TFEB is phosphorylated by MTORC1, Akt, PKC, ERK1/2, etc., and binds to the chaperone YWHA/14-3-3 (tyrosine 3-monooxygenase/tryptophan 5-monooxygenase activation protein) to remain in the cytosol and inactivation^42,103,104^. Upon growth factor or amino acid depletion, dephosphorylated TFEB translocates into the nucleus and transcriptionally actives target genes, including TFEB itself. Although previous studies have reported Akt and PKC-dependent activation of TFEB by trehalose or HEP-14^37,43^, how to induce TFEB activation without affecting the cellular kinome activity remains elusive. Herein, we found that naringenin directly targeted the YWHA/14-3-3 epsilon-TFEB interface to regulate TFEB activation and expression, without affecting MTORC1, Akt, PKC activation. Of interest, the recent structural analysis revealed that TFEB binds to 14-3-3 and retains TFEB inactivation via a hydrophobic loop that is distinct from most 14-3-3 binding proteins^47^. In the current study, we revealed that naringenin specifically occupied this hydrophobic groove without interfering with 14-3-3 binding to other partners, such as FOXO4. Targeting the 14-3-3-TFEB interface may lead to potential drug discovery for the treatment of not only aneurysms but also atherosclerosis or other diseases with aberrant macrophage lysosome biogenesis.

## Materials and methods

### Computational pipeline

CMap is based on the principle that drugs with similar gene expression profiles often share similar mechanisms and affect the same chemical and physiological processes and may therefore treat similar diseases. To identify candidate drugs for the treatment of AAA, we first downloaded the dataset of drug-responsive gene expression profiles from CMap (www.broad.mit.edu/cmap)^105^. Using doxycycline as a seed drug, we then performed a correlation analysis between doxycycline and all the other drugs that had been integrated into CMap on an in-house computer program. The top drugs that showed a significant and high correlation with doxycycline were then considered candidate drugs for the treatment of AAA. The framework for the identification of candidate drugs based on the above drug-repositioning strategy is illustrated in Fig. 1a.

### Materials

Angiotensin II (A9525), doxycycline (10592-13-9), naringenin (67604-48-2), and gelatin (9000-70-8) were purchased from Sigma Chemical Co. (Saint Louis, Missouri). The antibody against Mac3 (sc-32790) was purchased from Santa Cruz Biotechnology (Santa Cruz, California). Mouse inflammatory cytokine bead array (CBA) kits (552364) were purchased from BD Biosciences (Clontech, California). Antibodies against eIF5 (13894S), TFEB (4240S), CD68 (97778S), CD206 (24595 S), caspase-1 (2225S), IL-1β (12163S), NLRP3 (13158S), caspase-3 (9662S) and Bax (2774S) were obtained from Cell Signaling Technology (Arundel, Australia). Antibodies against GAPDH (60004-1-Ig), β-tubulin (10068-1-AP), Histone H3 (17168-1-AP), and TFE3 (14480-1-AP) were purchased from Proteintech Group (Wuhan, China). DQ^TM^ Ren BSA (# D12051), Lysotracker^TM^ Deep Red (L12492), and Lysotracker^TM^ Green DND-26 (L7526) were purchased from Life Technologies (San Diego, CA). Fluorescein isothiocyanate (FITC) anti-mouse CD45 (103107), APC anti-mouse CD11b (101211), PE anti-mouse Ly6G (127607), APC anti-mouse CD86 (105011), APC-anti mouse CD31 (102409), and APC anti-mouse F4/80 (157305) used in flow cytometry were purchased from BioLegend (San Diego, CA)

### Animals

All animal studies followed the guidelines of the Animal Care and Use Committee of Peking University. The Tfeb^flox^ mice were generated by using CRISPR/Cas9 technology. The mouse TFEB gene (Gene ID: 21425) has several transcripts, and its exon 4–5 includes a conserved protein functional domain: the HLH domain. Thus, guide RNAs were designed before exon 4 and after exon 5. The plasmids harboring the sgRNA, the donor plasmids, and cas9 mRNA was purified and co-injected into the cytoplasm of zygotes of C57BL/6 J mice. After a short in vitro culture, the injected blastocysts were transferred into pseudopregnant female mice. The founder mice with flox insertion were mated with WT mice. The genotypes of their offspring were verified by PCR using the following primers: forward: 5′-TTGCTCCTGCCTGTTGAATTCCAT-3′, reverse 5′-AACCAGTCGAGCTACTAGGGAGAG-3′.

### Cell culture

Then primary mouse peritoneal macrophages were isolated from thioglycollate-injected 8–10 weeks old male C57BL/6J mice, TFEB^flox/flox^ mice, and TSC1^flox/flox^ mice as described in a previous study^106^. Bone marrow cells of 8–10 weeks old male C57BL/6 J mice TFEB^flox/flox^ mice and TSC1^flox/flox^ mice were isolated and differentiated into BMDMs in RPMI 1640 medium (Life Technologies, Grand Island, NY, USA) containing 30% conditioned medium from L929 cells as previously described^107^. HEK293A cells were purchased from the American Type Culture Collection (Manassas, VA, USA) and cultured in high-glucose DMEM supplemented with 10% FBS, 100 units/mL penicillin, and 100 μg/mL streptomycin. All cells were maintained in a humidified 5% CO_2_ incubator at 37 °C.

### Calcium Phosphate-Induced AAA model in C57BL/6J mice

All studies followed the guidelines of the Animal Care and Use Committee of Peking University. Male C57BL/6J mice that were 12 weeks old were placed under anesthesia. The infrarenal region of the abdominal aorta was isolated through a midline incision. A small piece of gauze that had been soaked in a 0.5 mol/L CaCl_2_ solution was applied perivascularly for 10 min and then replaced with another piece of PBS-soaked gauze for 5 min to form calcium phosphate (CaPO_4_) crystals. The control mice received a similar treatment with 0.5 mol/L NaCl-soaked gauzes that was applied for 10 min, followed by PBS-soaked gauze that was applied for another 5 min^108^. On postoperative day 2, the NaCl control group was given water, while the CaPO_4_-treated mice were divided into three different treatment groups and were intragastrically administered either water, naringenin (50 mg/kg/day)^109,110^, or doxycycline (100 mg/kg/day) every day for one week. Systolic blood pressure was monitored in conscious mice by tail-cuff plethysmography with a BP-98A intravascular blood pressure transducer. Fourteen days later, the mice were killed, and blood samples were collected. The maximum diameter of the infernal aorta was measured by ex vivo measurement under ImageJ software, and the infrarenal regions of the abdominal aortas from the right renal branch to the iliac artery were excised.

For investigating the effects of naringenin on established abdominal aortic aneurysms, CaPO_4_ was applied to 12-week-old male C57BL/6J mice (*n* = 30) as described above. Seven days later, these mice with AAA were further allocated into two groups by a random number table according to body weight and external diameter of the abdominal aorta: a naringenin group (*n* = 15) and a vehicle group (*n* = 15). Another 7 days later, the mice were sacrificed for ex vivo measurement.

For confirmation of whether the role of naringenin on aneurysm formation is macrophage TFEB dependent, CaPO_4_ was also applied to 12-week-old male TFEB^*flox/flox*^ mice and TFEB^*MφKO*^ mice as described above.

For validation of the role of naringenin on established AAAs is macrophage TFEB dependent, CaPO_4_ was perivascularly applied to 12-week-old male TFEB^*MφKO*^ mice (*n* = 18) above. 7 days later, we used ultrasonography to measure the maximal abdominal aortic diameter. AAA was defined as a 50% increase in the external diameter of the abdominal aorta. According to this criterion, 9 out of the 18 mice developed AAA. These mice with AAA were further allocated into two groups by a random number table according to body weight and external diameter of the abdominal aorta: a naringenin group (*n* = 4) and a vehicle group (*n* = 5). Another 7 days later, the mice were sacrificed for ex vivo measurement.

### Angiotensin II-induced AAA Model in ApoE^−/−^ Mice

Alzet osmotic minipumps (model 2004) containing AngII were subcutaneously implanted into male, 16-week-old ApoE^−/−^ mice. AngII was infused into the mice at a rate of 1000 ng/kg/min for 28 days. On postoperative day 2, the mice were randomly divided into two groups by a random number table according to the bodyweight: one group was intragastrically administered water daily for 4 weeks, while the second group received naringenin (50 mg/kg/day). Systolic blood pressure was monitored in conscious mice by tail-cuff plethysmography with a BP-98A intravascular blood pressure transducer. The mice were killed, blood samples were collected, and the suprarenal regions of the abdominal aortas from the proximal aorta to the right renal branch were excised.

For analysis of the role of naringenin in established AAA, 4-month-old male ApoE^−/−^ mice (*n* = 70) were subcutaneously implanted with minipumps containing AngII and infused with AngII at a rate of 1000 ng/kg/min for 28 days. Eight mice died early in this study due to thoracic aortic dissection. We used ultrasonography to measure the maximal suprarenal aortic diameter at the peak of systole with the caliper measurement feature of the Veov 770TM Imaging System (Visual Sonics, Inc., Toronto, Canada in B-mode). AAA was defined as a 50% increase in the external diameter of the abdominal aorta. According to this criterion, 42 out of the 62 mice developed AAA. These mice were further allocated into two groups by a random number table according to body weight, blood pressure, and the external diameter of the abdominal aorta: a naringenin group (*n* = 21) and a vehicle group (*n* = 21). All 42 mice were infused with AngII at a rate of 1000 ng/kg/min for an additional 28 days, and the surviving mice were then imaged with ultrasonography and sacrificed for ex vivo measurement.

### Analysis of plasma lipid levels

Plasma total cholesterol, triglycerides, low-density lipoprotein, and high-density lipoprotein were assayed with kits from Zhong Sheng Biotechnology (Beijing, China)^111^.

### Morphology

Mice were killed and perfused with a 4% paraformaldehyde solution in PBS. The infrarenal aortas of the C57BL/6J mice, TFEB^*flox/flox*^ mice, TFEB^*MφKO*^ mice, and the suprarenal aortas of the ApoE^−/−^ mice were immediately divided into two sections, and serial cryosections (6 μm thickness, 300 μm apart) were made.

### Immunofluorescence staining

Frozen sections were incubated with anti-CD45 (1:200, BD), anti-Mac3-6 (1:50, Santa Cruz), anti-CD68 (1:200, AbD Serotec), anti-CD206 (1:200, Cell Signaling Technology), and anti-TFEB (1:200, Bethyl) antibodies, followed by incubation with secondary antibodies (1:300) (Thermo Fisher, Inc., Waltham, MA). The nuclei were counterstained with Hoechst 33342. The fluorescence signal was measured with a confocal laser scanning microscope (Leica, Germany).

### Cytokine assay

Plasma cytokines were measured with a mouse inflammatory Cytokine Cytometric Bead Array Kit (CBA, BD Biosciences).

### In situ zymography

OCT-embedded, fresh abdominal aortic cryosections were analyzed with in situ zymography using the MMP fluorogenic substrate DQ-gelatin-FITC (Invitrogen). Cryosections were incubated with 40 μg/ml (in PBS) of the quenched fluorogenic substrate DQ-gelatin-FITC for 1 h at 37 °C. The gelatin with a fluorescent tag remained caged (no fluorescence) until the gelatin was cleaved by a gelatinase. The excess fluorogenic substrate was washed with PBS and photographed with a confocal microscope.

### Gelatin zymography

The infrarenal aortas from the C57BL/6J mice treated with CaPO_4_ for one week and the suprarenal aortas from the ApoE^−/−^ mice that were subcutaneously infused with AngII (1000 ng/kg/min) for 4 weeks were incubated in a culture medium for 24 h. The conditioned medium was then electrophoresed on sulfate-polyacrylamide gel electrophoresis (SDS-PAGE) gels containing 1 mg/ml gelatin. The gels were washed twice in 2.5% Triton X-100, incubated for 24 h (37 °C) in zymography buffer (50 mM Tris-HCl [pH 7.5], 150 mM NaCl, 10 mM CaCl_2_, and 0.05% sodium azide) and stained with Coomassie brilliant blue.

### Quantitative RT-PCR and western blot analysis

RT-PCR amplification was performed with the Mx3000 Multiplex Quantitative PCR System (Stratagene Corp, La Jolla, California), SYBR Green I reagent, and the 18S internal control for normalization.

The primer sequences used in the RT-PCR experiments are presented in Supplementary Table S8. All amplification reactions were conducted over 40 cycles (an initial stage of 7 min at 94 °C and then a three-step program of 30 s at 94 °C, 30 s at 58 °C, and 30 s at 72 °C) and were performed in duplicate.

Mouse tissue and cultured cells containing equal amounts of total protein were resolved by 12% SDS-PAGE for Western blot analysis. The blots were incubated with a primary antibody and an IRDye 700DX-conjugated secondary antibody (Rockland, Inc.). The immunofluorescence signal was detected by the Odyssey infrared imaging system (LI-COR Biosciences, Lincoln, Nebraska).

### TUNEL assay

Apoptotic cells were detected with the Tdt-mediated dUTP-biotin nick end labeling assay (TUNEL, Promega Company, USA).

### RNA-seq analysis

Vehicle or naringenin (200 µM) treated BMDMs were prepared for RNA isolation (*n* = 3 for each group) following the Trizol protocol. RNA samples were resuspended in nuclease-free water (Ambion) and genomic DNA contamination was removed using the commercially available Turbo-DNA free kit (Invitrogen). rRNA removal was performed using 50–100 ng total RNA input and a modified protocol for the Ribo-Zero Epidemiology Gold rRNA removal kit (Illumina). The rRNA-depleted samples were purified by precipitating the RNA. The cDNA libraries were individually prepared from each sample by performing a series of procedures including poly(A) enrichment, RNA fragmentation, random hexamer primed cDNA synthesis, linker ligation, size selection, and PCR amplification. The quality and quantification of cDNA libraries were performed by using the Qubit 2.0 Fluorometer (Thermo Scientific) and Agilent 2100 Bioanalyzer (Agilent Technologies, Singapore). The libraries were then sequenced using HiSeq Illumina 2500 sequencing platform (Illumina, San Diego, CA).

### Luciferase assay

The 2× CLEAR-Luc plasmid was obtained from Addgene. The GATA3/IRF4/STAT6-Luc plasmids were conducted by inserting the promoter sequences of mouse GATA3/IRF4/STAT6 genes into pGL3-basic vectors, respectively. The primers used for subcloning were shown in Supplementary Table S10. Luciferase activity was measured using the Steady-Glo® Luciferase Assay System (Promega Corporation). Briefly, HEK293A cells transfected with 2× CLEAR-Luc plasmid or GATA3/IRF4/STAT6-Luc plasmid were cultured in 100 µl of medium using a 96-well white plate, and 100 µl of CCLR (cell culture lysis reagent) with luciferase substrate was added to each well. Luminescence was measured using a SpectroMax M3 Multimode microplate reader (Molecular Devices).

### Autophagic flux assay

The autophagic flux was measured using DQ^™^ Red BSA, which is labeled to a high degree with red fluorescent BODIPY^®^ TR-X dye. As previously reported, DQ^™^ Red BSA will accumulate in autophagosomes and will be combined with functional lysosomes to generate autolysosomes. Then, DQ^™^ Red BSA will be cleaved by proteases in the autolysosomes. The proteolysis of this conjugate can be easily monitored because digestion results in dequenching and releases brightly fluorescent fragments. BMDMs, primary rat vascular smooth muscle cells, and HUVECs that were pretreated with naringenin (0, 50, 100, 200 μM for 12 h) or EBSS (positive control for 30 min) were incubated with DQ^™^ Red BSA (100 μg/ml) for 60 min. Then, flow cytometric analysis was used to detect the RFP fluorescence intensity.

### Lysosomal degradation assay

HEK293A cells were transfected with a tandem fluorescent mRFP-GFP-tagged LC3 plasmid using Lipofectamine 2000. The expression of GFP and mRFP were visualized with an Olympus FV1000 laser scanning confocal microscope (Olympus, Tokyo, Japan). Images were acquired using FV10-ASW3.0 software. Yellow (merged GFP signal and RFP signal) puncta represent early autophagosomes, while red (RFP signal alone) puncta indicate autolysosomes. The lysosomal degradation activity was evaluated by the color change of GFP/mRFP.

### Generating of TSC1^−/−^ macrophages

Six- to eight-week-old male TSC1^flox/flox^ mice were kindly given by Prof. Zhang from Peking University. Then primary mouse peritoneal macrophages were isolated from thioglycollate-injected TSC1^flox/flox^ mice. Briefly, each TSC1^flox/flox^ mouse was intraperitoneally injected with 5 mL of 2.9% thioglycollate. Three days later, the cells were perfused from the peritoneal cavity and cultured with RPMI 1640 medium (Life Technologies, Grand Island, NY, USA) including 10% fetal bovine serum (FBS) (Life Technologies, Grand Island, NY, USA). Twenty-four hours later, the cells were infected with adenovirus that encoded green fluorescent protein (GFP) (10 MOI) or cyclization recombination enzyme (Cre) (10 multiplicity of infection (MOI)) to generate wild type (WT) macrophages or TSC1^−/−^ macrophages, respectively.

### Flow cytometry analysis and macrophage cell sorting

For analyzing the role of naringenin on macrophage TFEB and target gene expression, 20 male C57BL/6J mice that were 10 weeks old were placed under anesthesia. Then the infrarenal region of the abdominal aorta was isolated and incubated with 0.5 mol/L CaCl_2_ solutions perivascularly for 10 min followed by PBS incubation for another 5 min to form calcium phosphate (CaPO_4_) crystals. On a postoperative day 2, the mice were divided into 2 groups by intragastrically administered water or naringenin (50 mg/kg/day) every day for 7 days. Then the mice were killed and the abdominal aortas were collected and gathered together within each group then digested into single cells using 1 mL aortic dissociation enzyme solution as described previously^112^. Macrophages in single-cell suspensions derived from mouse aortas were co-labeled with FITC anti-F4/80 and PE anti-CD11b antibodies. Double positive cells were sorted as aortic macrophages by BD FACS Aria II SORP (BD Biosciences, San Diego, CA, USA) for further RNA isolation and RT-qPCR analysis.

### Construction of adenovirus

Adenoviruses encoding GFP and mouse TFEB were generated by cloning the coding region of mouse TFEB and control GFP into the AdTrack-CMV (cytomegalovirus) vector (Agilent Technologies). Next, the coding region was cloned from the Ad-track vector to the AdEasy vector by homologous recombination in *Escherichia coli*. The adenoviruses were packaged in HEK293A cells and purified by CsCl_2_ density gradient ultracentrifugation. Adenovirus titration was determined by the Adeno-XTM qPCR titration kit (Clontech).

### Chromatin immunoprecipitation assay

ChIP assays were performed with an EZ-ChIP kit (Millipore) according to the manufacturer’s protocol. Purified precipitated DNA was used as a template for PCR. The primers used for ChIP-PCR are listed in Supplementary Table S9.

### Thermal shift experiment

BMDMs (106 cells) were harvested, and cell lysates were incubated with either vehicle (DMSO) or naringenin (200 µM) (final DMSO content). For every volume, 50 µl of the mixture was transiently heated to different temperatures ranging from 42 °C to 75 °C for 5 min using a TaKaRa PCR Thermal Cycler Dice^®^ Gradient (TaKaRa), followed by cooling at room temperature for 5 min. After the heating step, the plate was centrifuged at 12,000*g* for 10 min at 4 °C to separate the soluble proteins from the aggregates, and the supernatant samples were analyzed by SDS-PAGE followed by western blotting.

For the ITDFR-CETSA experiment, naringenin was serially diluted to generate dose–response curves (from 0 to 102.4 µM). Cell lysates were treated with each concentration in a 50 µl system in a 96-well PCR plate for 5 min at 55 °C, followed by cooling at room temperature for 5 min. Then, the heat-treated samples were centrifuged at 12,000*g* for 10 min at 4 °C, and the level of 14-3-3ε was analyzed by SDS-PAGE followed by western blotting.

### Expression and purification of 14-3-3ɛ

Recombinant baculovirus encoding full-length mouse 14-3-3ɛ was generated using the Bac-to-Bac baculovirus expression system (Invitrogen, CA, USA). Briefly, suspended Sf9 cells at a density of 2 × 10^6^ cells/mL were infected with P2 virus stocks at an MOI of 2 for 48 h. The medium supernatant of virus-infected cells was collected by centrifugation at 4000 rpm for 15 min after the addition of 5 mM imidazole and protease inhibitor cocktail (Roche, Basel, Switzerland). The supernatant was incubated with equilibrated Ni-NTA resin (Roche, Basel, Switzerland) with gentle rotation at 4 °C for 12 h. The resin was transferred to a column and washed with 20-bed volumes of lysis buffer. 14-3-3ɛ was eluted by washing the column with 10-bed volumes of a 0.05–0.3 M linear gradient of imidazole in lysis buffer. The protein was dialyzed against lysis buffer and then concentration by ultrafiltration. The protein was determined to be at least 70% pure by 8% SDS-PAGE.

### Fluorescence polarization-based binding assay

Serial dilutions of His-14-3-3ε were made in assay buffer (containing 25 mM HEPES (pH 7.5), 100 mM NaCl, 1 mg/ml BSA, and 0.05% CHAPS) covering a range of 1–4000 nM and dispensed on a black 384-well nonbinding microplate (Greiner Bio-One GmbH, Frickenhausen, Germany) in triplicate (10 μl). Blank controls contained only buffer (10 μl). Ten microliters of the 2× Spindlin1 dilution and 10 μl of 10 nM p-TFEB-FITC were dispensed on the microplate, resulting in a final assay volume of 20 μl per well. Fluorescence polarization (FP) was measured from these wells after incubation for 60 min at 25 °C with the help of an EnVisionTM plate reader (PerkinElmer). For the fluorescence polarization displacement assay, a 2× concentrated pool solution containing 20 nM His-14-3-3ε and 20 nM p-TFEB-FITC was prepared in assay buffer. For IC_50_ determination experiments, a 1:1 naringenin dilution series (2x concentrated) was prepared. Ten microliters of each naringenin solution were dispensed onto a black 384-well nonbinding microplate (Greiner) in triplicate. Ten microliters of an appropriate DMSO solution in assay buffer were dispensed into 6 wells for positive controls and into another 6 wells for negative controls. Ten microliters of His-14-3-3ε/p-TFEB-FITC were added to each well-containing naringenin solution and the positive controls. Ten microliters of the p-TFEB peptide solution were added to the negative control wells. The final concentrations were 10 nM and 10 nM His-14-3-3ε and p-TFEB peptide, respectively, in a final assay volume of 20 μl. Six wells contained 20 μL of DMSO solution in assay buffer to generate blank controls. The microplate was then incubated for 60 min at 25 °C. GraphPad Prism was used for visualization and calculation of the IC_50_ values by plotting inhibition values against logarithmic compound concentrations and applying a sigmoidal dose-response fit (variable slope). The setup of the FP assay for the p-control peptide and His-14-3-3ε was realized by following the same optimization route as described for the p-TFEB peptide.

### ITC assay

The ITC assay was performed using a MicroCalorimeter ITC200 (GE Healthcare Life Sciences, USA) at 25 °C. Prior to the experiment, protein samples were dialyzed in a buffer containing 20 mM Tris-HCl, pH 7.5, and 200 mM NaCl. Naringenin was placed in the sample cell, and the YWHAE/14-3-3ε protein was placed in the syringe of the instrument. YWHAE/14-3-3ε protein (~50 μM) was sequentially injected into the stirred calorimeter cell initially containing naringenin (~5 μM) with an injection sequence of 20 × 2 µl at 2 min intervals.

The heat of dilution obtained by the titration of the peptide into the buffer was subtracted. The integrated corrected and concentration-normalized peak areas of the raw data were finally fitted with a model of one binding site using ORIGIN 7.0 (OriginLab).

### Identification of naringenin-binding proteins

The identification of naringenin-binding proteins was performed as previously reported^44^. Briefly, cell lysates of BMDMs from 3 independent biological replicates were aliquoted in equivalent volumes containing 100 mg of proteome sample and incubated for 10 min at 25 °C with vehicle or naringenin (final concentration: 200 µM). Proteinase K from *Tritirachium album* (Sigma Aldrich) was added simultaneously to all the proteome-naringenin samples at a proteinase K to substrate mass ratio of 1:100 and incubated at 25 °C for 5 min. Digestion reactions were stopped by heating samples for 3 min at 98 °C in a thermocycler followed by the addition of sodium deoxycholate (Sigma Aldrich) to a final concentration of 5%. The samples were then heated again at 98 °C for 3 min in a thermocycler. Protein fragments from the proteinase K-limited proteolysis step were reduced with 5 mM Tris(2-carboxyethyl) phosphine (Thermo Fisher Scientific) for 40 min at 37 °C and then alkylated by incubating for 30 min at 25 °C with 20 mM iodoacetamide (Sigma Aldrich) in the dark. The samples were diluted with 0.1 M ammonium bicarbonate to a final concentration of 1% sodium deoxycholate and predigested with lysylendopeptidase (Wako Chemicals) at an enzyme-substrate ratio of 1:100 for 4 h at 37 °C. Digestions were completed by treatment with sequencing-grade porcine trypsin (Promega) at an enzyme-substrate ratio of 1:100 for 16 h at 37 °C. Trypsin was inactivated to stop peptide digestions by adding a volume of formic acid that lowered the pH to less than 2. Acidified peptide mixtures were loaded onto Sep-Pak tC18 cartridges or into 96-well elution plates (Waters), desalted, and eluted with 50% acetonitrile–0.1% formic acid. The samples were dried in a vacuum centrifuge, solubilized in 0.1% formic acid, and immediately analyzed by mass spectrometry.

### siRNA transfection

The Amaxa Mouse Macrophage Nucleofector Kit (Lonza Cologne AG, Cologne, Germany) was used to transfer siRNAs into mouse peritoneal macrophages. The transfection procedures followed the manufacturers’ instructions. The sequence of siRNA targeting mouse TSC2 is sense 5′-GCAUGCAGUUCUCACCUUATT-3′, antisense 5′-UAAGGUGAGAACUGCAUGCTT-3′. The sequence of siRNA targeting mouse 14-3-3 epsilon is: sense 5′-GGAGUACCGGCAAAUGGUUTT-3′, antisense 5′-AACCAUUUGCCGGUACUCCTT-3′. The sequence of scrambled siRNA is sense 5′-UUCUCCGAACGUGUCACGUTT-3′, antisense 5′-ACGUGACACGUUCGGAGAATT-3′.

### Flow cytometric analysis

Mouse bone marrow cells were labeled using FITC-conjugated anti-mouse CD45, PE-conjugated anti-mouse Gr-1, and APC-conjugated anti-mouse CD11b antibodies to identify myeloid cells. Blood cells were analyzed using FITC anti-CD45 and APC anti-CD11b together with PE-conjugated anti-mouse Ly6C or Ly6G antibodies to identify monocytes or neutrophils, respectively, following the disruption of red blood cells using RBC lysis buffer (Tiangen Biotech, Inc., Beijing, China). Labeling with PE anti-F4/80 or APC-CD31 together with LysoTracker (Thermo Fisher Scientific) was performed to identify the lysosomal numbers in macrophages and ECs in single-cell suspensions derived from mouse aortas.

### Statistical analysis

All results are expressed as the mean ± standard error of the mean and were analyzed by the Shapiro–Wilk normality test to evaluate whether the sample has a normal distribution. After confirmation of the normal distribution of the data, Student’s *t* test was used for a two-group comparison of the effects of naringenin on the aortic diameter, plasma cytokine level, inflammatory cell infiltration, MMP activity, etc. For comparisons among more than two groups, ANOVA with Bonferroni test when appropriate was used to compare and evaluate the effect of naringenin and doxycycline on the diameter of the aorta, inflammatory cell infiltration, MMP activity and apoptosis in the CaPO_4_-treated mice, etc. The chi-square test was used to analyze the incidence of aneurysms. The nonparametric Kruskal–Wallis test with a dumn post hoc test was used to analyze the elastin grade of aortas.

## Supplementary information

Supplementary Information

## References

[1] Nordon IM, Hinchliffe RJ, Loftus IM, Thompson MM (2011). Pathophysiology and epidemiology of abdominal aortic aneurysms. Nat. Rev. Cardiol..

[2] Daugherty A, Cassis LA (2004). Mouse models of abdominal aortic aneurysms. Arterioscler. Thromb. Vasc. Biol..

[3] Habashi JP (2011). Angiotensin II type 2 receptor signaling attenuates aortic aneurysm in mice through ERK antagonism. Science.

[4] Kristensen KE (2015). Angiotensin-converting enzyme inhibitors and angiotensin II receptor blockers in patients with abdominal aortic aneurysms: nation-wide cohort study. Arterioscler. Thromb. Vasc. Biol..

[5] Wang YD, Liu ZJ, Ren J, Xiang MX (2018). Pharmacological therapy of abdominal aortic aneurysm: an update. Curr. Vasc. Pharm..

[6] Walton LJ (1999). Inhibition of prostaglandin E2 synthesis in abdominal aortic aneurysms: implications for smooth muscle cell viability, inflammatory processes, and the expansion of abdominal aortic aneurysms. Circulation.

[7] Tomida S (2019). Indomethacin reduces rates of aortic dissection and rupture of the abdominal aorta by inhibiting monocyte/macrophage accumulation in a murine model. Sci. Rep..

[8] Qu XA, Rajpal DK (2012). Applications of Connectivity Map in drug discovery and development. Drug Discov. Today.

[9] Corbett A (2012). Drug repositioning for Alzheimer’s disease. Nat. Rev. Drug Discov..

[10] Liu W (2018). Revisiting Connectivity Map from a gene co-expression network analysis. Exp. Ther. Med..

[11] Gao Y, Kim S, Lee YI, Lee J (2019). Cellular stress-modulating drugs can potentially be identified by in silico screening with Connectivity Map (CMap). Int. J. Mol. Sci..

[12] Lamb J (2006). The Connectivity Map: using gene-expression signatures to connect small molecules, genes, and disease. Science.

[13] Yu M, Chen C, Cao Y, Qi R (2017). Inhibitory effects of doxycycline on the onset and progression of abdominal aortic aneurysm and its related mechanisms. Eur. J. Pharmacol..

[14] Dodd BR, Spence RA (2011). Doxycycline inhibition of abdominal aortic aneurysm growth: a systematic review of the literature. Curr. Vasc. Pharmacol..

[15] Roman-Goldstein S (1994). Osmotic blood-brain barrier disruption: CT and radionuclide imaging. Am. J. Neuroradiol..

[16] Hasbullah (1996). Medication of feedlot calves infected with Eimeria spp. by a combination of sulfamonomethoxine and ormetoprim. J. Vet. Med. Sci..

[17] Abe A (1992). Improved inhibitors of glucosylceramide synthase. J. Biochem..

[18] in *Drugs and Lactation Database (LactMed)* (2006).

[19] Fan HY (2021). Insight into the molecular mechanism of podophyllotoxin derivatives as anticancer drugs. Front. Cell Dev. Biol..

[20] Shah Z (2021). Podophyllotoxin: History, recent advances and future prospects. Biomolecules.

[21] Bittar GT, Graff-Radford SB (1993). The effects of streptomycin/lidocaine block on trigeminal neuralgia: a double blind crossover placebo controlled study. Headache.

[22] Pillay VK (1976). Some side-effects of alpha-methyldopa. S. Afr. Med. J..

[23] Hou X (2021). Akkermansia muciniphila potentiates the antitumor efficacy of FOLFOX in colon cancer. Front. Pharmacol..

[24] Mulvihill EE (2009). Naringenin prevents dyslipidemia, apolipoprotein B overproduction, and hyperinsulinemia in LDL receptor-null mice with diet-induced insulin resistance. Diabetes.

[25] Assini JM (2013). Naringenin prevents cholesterol-induced systemic inflammation, metabolic dysregulation, and atherosclerosis in Ldlr–/– mice. J. Lipid Res..

[26] Burke AC (2019). Naringenin enhances the regression of atherosclerosis induced by a chow diet in Ldlr–/– mice. Atherosclerosis.

[27] Zobeiri M (2018). Naringenin and its nano-formulations for fatty liver: cellular modes of action and clinical perspective. Curr. Pharm. Biotechnol..

[28] Trollope AF, Golledge J (2011). Angiopoietins, abdominal aortic aneurysm and atherosclerosis. Atherosclerosis.

[29] Golledge J (2019). Abdominal aortic aneurysm: update on pathogenesis and medical treatments. Nat. Rev. Cardiol..

[30] Davis FM, Rateri DL, Daugherty A (2015). Abdominal aortic aneurysm: novel mechanisms and therapies. Curr. Opin. Cardiol..

[31] Mulvihill EE (2010). Naringenin decreases progression of atherosclerosis by improving dyslipidemia in high-fat-fed low-density lipoprotein receptor-null mice. Arterioscler. Thromb. Vasc. Biol..

[32] Xie X (2012). Doxycycline does not influence established abdominal aortic aneurysms in angiotensin II-infused mice. PLoS ONE.

[33] Perera RM, Di Malta C, Ballabio A (2019). MiT/TFE family of transcription factors, lysosomes, and cancer. Annu. Rev. Cancer Biol..

[34] Chauhan S (2013). ZKSCAN3 is a master transcriptional repressor of autophagy. Mol. Cell.

[35] Napolitano G (2018). mTOR-dependent phosphorylation controls TFEB nuclear export. Nat. Commun..

[36] Puertollano R, Ferguson SM, Brugarolas J, Ballabio A (2018). The complex relationship between TFEB transcription factor phosphorylation and subcellular localization. EMBO J..

[37] Li Y (2016). Protein kinase C controls lysosome biogenesis independently of mTORC1. Nat. Cell Biol..

[38] Medina DL (2015). Lysosomal calcium signalling regulates autophagy through calcineurin and TFEB. Nat. Cell Biol..

[39] Vega-Rubin-de-Celis S, Pena-Llopis S, Konda M, Brugarolas J (2017). Multistep regulation of TFEB by MTORC1. Autophagy.

[40] Settembre C (2012). A lysosome-to-nucleus signalling mechanism senses and regulates the lysosome via mTOR and TFEB. EMBO J..

[41] Palmieri M (2017). mTORC1-independent TFEB activation via Akt inhibition promotes cellular clearance in neurodegenerative storage diseases. Nat. Commun..

[42] Roczniak-Ferguson A (2012). The transcription factor TFEB links mTORC1 signaling to transcriptional control of lysosome homeostasis. Sci. Signal..

[43] Rusmini P (2019). Trehalose induces autophagy via lysosomal-mediated TFEB activation in models of motoneuron degeneration. Autophagy.

[44] Piazza I (2018). A Map of protein-metabolite interactions reveals principles of chemical communication. Cell.

[45] Silhan J (2009). 14-3-3 protein masks the DNA binding interface of forkhead transcription factor FOXO4. J. Biol. Chem..

[46] Obsilova V (2005). 14-3-3 protein interacts with nuclear localization sequence of forkhead transcription factor FoxO4. Biochemistry.

[47] Xu Y (2019). YWHA/14-3-3 proteins recognize phosphorylated TFEB by a noncanonical mode for controlling TFEB cytoplasmic localization. Autophagy.

[48] Muslin AJ, Tanner JW, Allen PM, Shaw AS (1996). Interaction of 14-3-3 with signaling proteins is mediated by the recognition of phosphoserine. Cell.

[49] Wakita D (2016). Role of interleukin-1 signaling in a mouse model of Kawasaki Disease-associated abdominal aortic aneurysm. Arterioscler. Thromb. Vasc. Biol..

[50] Johnston WF (2014). Inhibition of interleukin-1beta decreases aneurysm formation and progression in a novel model of thoracic aortic aneurysms. Circulation.

[51] Toldo S, Abbate A (2018). The NLRP3 inflammasome in acute myocardial infarction. Nat. Rev. Cardiol..

[52] Kelley N, Jeltema D, Duan Y, He Y (2019). The NLRP3 inflammasome: an overview of mechanisms of activation and regulation. Int. J. Mol. Sci..

[53] Shapouri-Moghaddam A (2018). Macrophage plasticity, polarization, and function in health and disease. J. Cell Physiol..

[54] Das A (2015). Monocyte and macrophage plasticity in tissue repair and regeneration. Am. J. Pathol..

[55] Raffort J (2017). Monocytes and macrophages in abdominal aortic aneurysm. Nat. Rev. Cardiol..

[56] Cheng Z (2018). Diverse roles of macrophage polarization in aortic aneurysm: destruction and repair. J. Transl. Med..

[57] Lawrence T, Natoli G (2011). Transcriptional regulation of macrophage polarization: enabling diversity with identity. Nat. Rev. Immunol..

[58] Juhas U, Ryba-Stanislawowska M, Szargiej P, Mysliwska J (2015). Different pathways of macrophage activation and polarization. Postepy Hig. Med. Dosw. (Online).

[59] Li C (2018). Macrophage polarization and meta-inflammation. Transl. Res..

[60] Zhu H, Qu X, Zhang C, Yu Y (2019). Interleukin-10 promotes proliferation of vascular smooth muscle cells by inhibiting inflammation in rabbit abdominal aortic aneurysm. Int. J. Clin. Exp. Pathol..

[61] Xu J (2019). Long non-coding RNA HIF1A-AS1 is upregulated in intracranial aneurysms and participates in the regulation of proliferation of vascular smooth muscle cells by upregulating TGF-beta1. Exp. Ther. Med..

[62] Quiros M (2017). Macrophage-derived IL-10 mediates mucosal repair by epithelial WISP-1 signaling. J. Clin. Invest..

[63] Vannella KM, Wynn TA (2017). Mechanisms of organ injury and repair by macrophages. Annu. Rev. Physiol..

[64] Reitamo S, Remitz A, Tamai K, Ledo I, Uitto J (1994). Interleukin 10 up-regulates elastin gene expression in vivo and in vitro at the transcriptional level. Biochem. J..

[65] Heidary Moghaddam R (2020). Naringenin and naringin in cardiovascular disease prevention: a preclinical review. Eur. J. Pharmacol..

[66] Salehi, B. et al. The therapeutic potential of Naringenin: a review of clinical trials. *Pharmaceuticals*10.3390/ph12010011 (2019). 10.3390/ph12010011 PMC6469163 30634637

[67] Assini JM, Mulvihill EE, Huff MW (2013). Citrus flavonoids and lipid metabolism. Curr. Opin. Lipidol..

[68] Kozlowska A, Szostak-Wegierek D (2014). Flavonoids–food sources and health benefits. Rocz. Panstw. Zakl. Hig..

[69] Stackelberg O, Bjorck M, Larsson SC, Orsini N, Wolk A (2013). Fruit and vegetable consumption with risk of abdominal aortic aneurysm. Circulation.

[70] Haring B (2018). Adherence to the dietary approaches to stop hypertension dietary pattern and risk of abdominal aortic aneurysm: Results from the ARIC study. J. Am. Heart Assoc..

[71] Bergwall S, Acosta S, Sonestedt E (2020). Intake of fibre and plant foods and the risk of abdominal aortic aneurysm in a large prospective cohort study in Sweden. Eur. J. Nutr..

[72] Ross SA, Ziska DS, Zhao K, ElSohly MA (2000). Variance of common flavonoids by brand of grapefruit juice. Fitoterapia.

[73] Zheng YZ, Deng G, Guo R, Chen DF, Fu ZM (2019). DFT studies on the antioxidant activity of naringenin and its derivatives: effects of the substituents at C3. Int. J. Mol. Sci..

[74] Hong Y, Yin Y, Tan Y, Hong K, Zhou H (2019). The flavanone, naringenin, modifies antioxidant and steroidogenic enzyme activity in a rat model of letrozole–induced polycystic ovary syndrome. Med. Sci. Monit..

[75] Escribano-Ferrer E, Queralt Regue J, Garcia-Sala X, Boix Montanes A, Lamuela-Raventos RM (2019). In vivo anti-inflammatory and antiallergic activity of pure naringenin, naringenin chalcone, and quercetin in mice. J. Nat. Prod..

[76] Zeng W, Jin L, Zhang F, Zhang C, Liang W (2018). Naringenin as a potential immunomodulator in therapeutics. Pharmacol. Res..

[77] Jin L, Zeng W, Zhang F, Zhang C, Liang W (2017). Naringenin ameliorates acute inflammation by regulating intracellular cytokine degradation. J. Immunol..

[78] Meijer CA (2013). Doxycycline for stabilization of abdominal aortic aneurysms: a randomized trial. Ann. Intern. Med..

[79] Baxter BT (2016). Non-invasive Treatment of Abdominal Aortic Aneurysm Clinical Trial (N-TA(3)CT): design of a Phase IIb, placebo-controlled, double-blind, randomized clinical trial of doxycycline for the reduction of growth of small abdominal aortic aneurysm. Contemp. Clin. Trials.

[80] Bayhan GI, Akbayram S, Ozaydin Yavuz G, Oner AF (2017). Cutaneous side effects of doxycycline: a pediatric case series. Cutan. Ocul. Toxicol..

[81] Bryant SG, Fisher S, Kluge RM (1987). Increased frequency of doxycycline side effects. Pharmacotherapy.

[82] Rebello CJ (2020). Safety and pharmacokinetics of naringenin: a randomized, controlled, single-ascending-dose clinical trial. Diabetes Obes. Metab..

[83] Jia B (2019). Naringenin ameliorates insulin resistance by modulating endoplasmic reticulum stress in hepatitis C virus-infected liver. Biomed. Pharmacother..

[84] Surendran K, Vitiello SP, Pearce DA (2014). Lysosome dysfunction in the pathogenesis of kidney diseases. Pediatr. Nephrol..

[85] Wallings RL, Humble SW, Ward ME, Wade-Martins R (2019). Lysosomal dysfunction at the centre of Parkinson’s disease and frontotemporal dementia/amyotrophic lateral sclerosis. Trends Neurosci..

[86] Usui F (2015). Inflammasome activation by mitochondrial oxidative stress in macrophages leads to the development of angiotensin II-induced aortic aneurysm. Arterioscler. Thromb. Vasc. Biol..

[87] Javaheri, A. et al. TFEB activation in macrophages attenuates postmyocardial infarction ventricular dysfunction independently of ATG5-mediated autophagy. *JCI Insight*10.1172/jci.insight.127312 (2019). 10.1172/jci.insight.127312 PMC6948771 31672943

[88] Cai, B. et al. USP5 attenuates NLRP3 inflammasome activation by promoting autophagic degradation of NLRP3. *Autophagy* 1–15 (2021). 10.1080/15548627.2021.1965426 PMC9196652 34486483

[89] Lv Q (2021). Lonicerin targets EZH2 to alleviate ulcerative colitis by autophagy-mediated NLRP3 inflammasome inactivation. Acta Pharm. Sin. B.

[90] Kim SH (2017). Ezetimibe ameliorates steatohepatitis via AMP activated protein kinase-TFEB-mediated activation of autophagy and NLRP3 inflammasome inhibition. Autophagy.

[91] Bauernfeind F (2011). Inflammasomes: current understanding and open questions. Cell Mol. Life Sci..

[92] Dale MA (2016). Elastin-derived peptides promote abdominal aortic aneurysm formation by modulating M1/M2 macrophage polarization. J. Immunol..

[93] Zhang Z (2018). Mouse macrophage specific knockout of SIRT1 influences macrophage polarization and promotes angiotensin II-induced abdominal aortic aneurysm formation. J. Genet. Genomics.

[94] Fornasa G (2012). A CD31-derived peptide prevents angiotensin II-induced atherosclerosis progression and aneurysm formation. Cardiovasc. Res..

[95] Adam M (2018). Systemic upregulation of IL-10 (interleukin-10) using a nonimmunogenic vector reduces growth and rate of dissecting abdominal aortic aneurysm. Arterioscler. Thromb. Vasc. Biol..

[96] Lu H (2020). Cyclodextrin prevents abdominal aortic aneurysm via activation of vascular smooth muscle cell transcription factor EB. Circulation.

[97] Mantovani A, Biswas SK, Galdiero MR, Sica A, Locati M (2013). Macrophage plasticity and polarization in tissue repair and remodelling. J. Pathol..

[98] Torra A (2018). Overexpression of TFEB drives a pleiotropic neurotrophic effect and prevents Parkinson’s disease-related neurodegeneration. Mol. Ther..

[99] Zhuang XX (2020). Pharmacological enhancement of TFEB-mediated autophagy alleviated neuronal death in oxidative stress-induced Parkinson’s disease models. Cell Death Dis..

[100] Song JX (2020). A small molecule transcription factor EB activator ameliorates beta-amyloid precursor protein and Tau pathology in Alzheimer’s disease models. Aging Cell.

[101] Sergin I (2017). Exploiting macrophage autophagy-lysosomal biogenesis as a therapy for atherosclerosis. Nat. Commun..

[102] Evans TD, Jeong SJ, Zhang X, Sergin I, Razani B (2018). TFEB and trehalose drive the macrophage autophagy-lysosome system to protect against atherosclerosis. Autophagy.

[103] Martina JA, Chen Y, Gucek M, Puertollano R (2012). MTORC1 functions as a transcriptional regulator of autophagy by preventing nuclear transport of TFEB. Autophagy.

[104] Martina JA (2014). The nutrient-responsive transcription factor TFE3 promotes autophagy, lysosomal biogenesis, and clearance of cellular debris. Sci. Signal..

[105] Sirota M (2011). Discovery and preclinical validation of drug indications using compendia of public gene expression data. Sci. Transl. Med..

[106] Fu Y (2012). Caveolin-1 plays a critical role in the differentiation of monocytes into macrophages. Arterioscler. Thromb. Vasc. Biol..

[107] Weischenfeldt J, Porse B (2008). Bone marrow-derived macrophages (BMM): isolation and applications. CSH Protoc..

[108] Yamanouchi D (2012). Accelerated aneurysmal dilation associated with apoptosis and inflammation in a newly developed calcium phosphate rodent abdominal aortic aneurysm model. J. Vasc. Surg..

[109] Annadurai T, Thomas PA, Geraldine P (2013). Ameliorative effect of naringenin on hyperglycemia-mediated inflammation in hepatic and pancreatic tissues of Wistar rats with streptozotocin- nicotinamide-induced experimental diabetes mellitus. Free Radic. Res..

[110] Orsolic N (2011). DNA-protective effects of quercetin or naringenin in alloxan-induced diabetic mice. Eur. J. Pharmacol..

[111] Luo QF, Sun L, Si JY, Chen DH (2008). Hypocholesterolemic effect of stilbenes containing extract-fraction from Cajanus cajan L. on diet-induced hypercholesterolemia in mice. Phytomedicine.

[112] Butcher, M. J., Herre, M., Ley, K. & Galkina, E. Flow cytometry analysis of immune cells within murine aortas. *J. Vis. Exp.*10.3791/2848 (2011). 10.3791/2848 PMC3196167 21750492

