# Supplementary material for: Targeting macrophage TFEB-14-3-3 epsilon Interface by naringenin inhibits abdominal aortic aneurysm
Source: Cell Discov. 2022 Mar 1;8:21. doi: 10.1038/s41421-021-00363-1 (PMC8885854; doi:10.1038/s41421-021-00363-1)
Supplement: Supplementary file 1 — Supplementary Information [file 41421_2021_363_MOESM1_ESM.pdf]

Supplementary Figure Legends

Supplementary Fig. S1. (a) Representative *in situ* zymography photography and quantification analysis of the infrarenal aortas of C57BL/6J mice. n=3 for each group. The data were analyzed using two-way ANOVA followed by Bonferroni test for post hoc comparison and are presented as the mean ± SEM of three independent experiments. *P<0.05. Scale bar = 50 μm. (b) Representative *ex vivo* gelatin zymography of infrarenal aortas of the mice with $CaPO_4$-induced AAA. n=3 mice for each group. (c) The mRNA levels of MMP-2 and MMP-9 in the abdominal aorta of the mice with $CaPO_4$-induced AAA. The data were analyzed using two-way ANOVA followed by Bonferroni test for post hoc comparison and are presented as the mean ± SEM of four independent experiments. *P<0.05. (d) Quantification of the positive immunofluorescence staining area of leukocytes (CD45 positive) and macrophages (Mac-3 positive) in the adventitia of the infrarenal aortas of the mice with $CaPO_4$-induced AAA. The data were analyzed using two-way ANOVA followed by Bonferroni test for post hoc comparison and are presented as the mean ± SEM of five independent experiments. *P<0.05. (e) Representative immunofluorescence (red) TUNEL staining of apoptotic cells and quantification of the number of TUNEL-positive cells per mm^2 area of infrarenal arteries in the mice with $CaPO_4$-induced AAA. Nuclei were stained with Hoechst (blue). The merged are (arrow) indicates the apoptotic cells. The data were analyzed using two-way ANOVA followed by Bonferroni test for post hoc comparison and are presented as the mean ± SEM of five independent experiments. *P<0.05. Scale bar = 50 μm. (f) Western blotting analysis and quantification of the protein levels of cleaved caspase-3 and Bax in the abdominal aorta of the mice with $CaPO_4$-induced AAA. The data were analyzed using two-way ANOVA followed by Bonferroni test for post hoc

comparison and are presented as the mean ± SEM of six independent experiments. *P<0.05. NS, no significance. (g) Representative elastin Van Gieson staining and quantification of the infrarenal abdominal aortas of the mice with $CaPO_4$-induced AAA. The data were analyzed using two-way ANOVA followed by Bonferroni test for post hoc comparison and are presented as the mean ± SEM. n=5-8 for each group. (h) Quantification of *in situ* zymography photography of the infrarenal aortas of the mice with AngII-induced AAA with vehicle or naringenin (50 mg/kg/day) gavage. The data were analyzed using unpaired two-tailed Student's *t*-test. n=3 for each group. (i) Representative *ex vivo* gelatin zymography of the infrarenal aortas of the mice with AngII-induced AAA. n=3 mice for each group. (j) Representative image and quantification of the positive immunofluorescence staining area of leukocytes (CD45 positive) and macrophages (Mac-3 positive) in the abdominal aortas of the mice with AngII-induced AAA. The data were analyzed using unpaired two-tailed Student's *t*-test. n=3 for each group. *P<0.05. (k) ELISAs of IL-6, MCP-1, IL-10, TNF-α and IFN-γ in the plasma of the AngII-induced mice with AAA. The data were analyzed using unpaired *t*-tests and are presented as the mean ± SEM. n=6 for each group. *P<0.05. (l) Representative elastin Van Gieson staining of the infrarenal abdominal aortas of the mice with AngII-induced AAA treated with water or naringenin (50 mg/kg/day) gavage.

Supplementary Fig. S2. (a) Heatmap of naringenin-induced genes related to lysosomes according to the CMap database. (b) Lysosomal number analysis of primary rat vascular smooth muscle cells (left) and HUVECs (right) stimulated with naringenin (0, 50, 100, 200 μM) for 12 h or EBSS-induced starvation for 30 min. The data were analyzed using one-way ANOVA

followed by Bonferroni test for post hoc comparison and are presented as the mean ± SEM of six independent experiments. *$P<0.05$. (c) DQ-red-BSA analysis of primary rat vascular smooth muscle cells (left) and HUVECs (right) stimulated with naringenin (0, 50, 100, 200 μM) for 12 h or CQ (1 μM) for 3 h. The data were analyzed using one-way ANOVA followed by Bonferroni test for post hoc comparison and are presented as the mean ± SEM of six independent experiments. *$P<0.05$. (d) KEGG analysis of mRNA expression that was upregulated by naringenin in the RNA-seq analysis. The X axis represents the -log10 (*p*-value). (e) RT-qPCR validation of lysosomal genes (ATP6V1A, NPC1, CTSS, etc.) in macrophages stimulated with vehicle or naringenin (200 μM) for 12 h. The data were analyzed using two-way ANOVA followed by Bonferroni test for post hoc comparison and are presented as the mean ± SEM of six independent experiments. *$P<0.05$. (f) 12-week-old male C57BL/6J mice were periadventitially treated with $CaPO_4$ and supplemented with vehicle or naringenin (50 mg/kg/day) for 7 days, and the abdominal aorta was separated and digested into single cells. Flow cell sorting was performed to isolate CD31-positive endothelial cells for lysosomal number analysis. n=10-15 mice for each group in each experiment. The data were analyzed using two-way ANOVA followed by Bonferroni test for post hoc comparison and are presented as the mean ± SEM of three independent experiments. *$P<0.05$. (g) RT-qPCR validation of TFE3, MiTF and Zkscan3 in macrophages stimulated with naringenin (0, 50, 100, 200 μM) for 12 h. The data were analyzed using one-way ANOVA followed by Bonferroni test for post hoc comparison and are presented as the mean ± SEM of six independent experiments. *$P<0.05$. (h) Western blotting analysis and quantification of the protein levels of p-PKC and p-Akt in the vehicle- or naringenin (0, 100, 200 μM for 12 hours)-treated BMDMs. The data were analyzed

using one-way ANOVA followed by Bonferroni test for post hoc comparison and are presented as the mean ± SEM of six independent experiments. (i) Primary peritoneal TSC1$^{flox/flox}$ macrophages that transfected with Ad-GFP (10 MOI) (here as WT macrophages) or with Ad-Cre (10 MOI) (here as TSC1$^{-/-}$ macrophages) for 48 hours, then treated with naringenin (0 µM, 100 µM, 200 µM) for another 12 hours followed by Western blot analysis and quantification of the protein levels of TSC1 and mTORC1 downstream phospho-S6 as well as phospho-4EBP1. The data were analyzed using two-way ANOVA followed by Bonferroni test for post hoc comparison and are presented as the mean ± SEM of six independent experiments. *P<0.05. (j) WT macrophages and TSC1$^{-/-}$ macrophages were treated with or without 200 µM naringenin for 12 hours, then followed by Lysotracker Red staining for 30 mins and analyzed by flow cytometry. The data were analyzed using two-way ANOVA followed by Bonferroni test for post hoc comparison and are presented as the mean ± SEM of six independent experiments. *P<0.05. (k) WT macrophages and TSC1$^{-/-}$ macrophages were treated with or without 200 µM naringenin for 12 hours, then followed by DQ-Red-BSA incubation for 30 mins and analyzed by live cell confocal microscopy. Scale bar = 10 µm. (l) RT-qPCR analysis of LAMP1, ATP6V1A, NPC1, NPC2 and CTSB expression in WT macrophages and TSC1$^{-/-}$ macrophages underwent naringenin treatment (200 µM, 12 hours). The data were analyzed using two-way ANOVA followed by Bonferroni test for post hoc comparison and are presented as the mean ± SEM of six independent experiments. *P<0.05. (m) Primary peritoneal macrophages that transfected with scrambled siRNA (50 nM) or TSC2 siRNA (50 nM) for 48 hours, then treated with naringenin (0 µM, 100 µM, 200 µM) for another 12 hours followed by Western blot analysis and quantification of the protein levels of TSC2 and mTORC1 downstream phospho-S6 as well

as phospho-4EBP1. The data were analyzed using two-way ANOVA followed by Bonferroni test for post hoc comparison and are presented as the mean ± SEM of six independent experiments. *P<0.05. (n) Primary peritoneal macrophages that transfected with scrambled siRNA (50 nM) or TSC2 siRNA (50 nM) for 48 hours, then treated with naringenin (200 μM) for another 12 hours followed by Lysotracker Red staining for 30 mins and analyzed by flow cytometry. The data were analyzed using two-way ANOVA followed by Bonferroni test for post hoc comparison and are presented as the mean ± SEM of six independent experiments. *P<0.05. (o) Primary peritoneal macrophages that transfected with scrambled siRNA (50 nM) or TSC2 siRNA (50 nM) for 48 hours, then treated with naringenin (200 μM) for another 12 hours followed by DQ-Red-BSA incubation for 30 mins and analyzed by live cell confocal microscopy. Scale bar = 10 μm. (p) RT-qPCR analysis of TSC2, LAMP1, NPC1 and NPC2 expression in scrambled siRNA transfected or TSC2 siRNA transfected macrophages underwent naringenin treatment (200 μM, 12 hours). The data were analyzed using two-way ANOVA followed by Bonferroni test for post hoc comparison and are presented as the mean ± SEM of six independent experiments. *P<0.05. (q) Upper, Representative flow cytometry results of macrophages in abdominal aortic single cells digested from $CaPO_4$ treated 10-week-old C57BL/6J mice with water or naringenin (50 mg/kg/day) gavage (10 mice per group) for 7 days. Lower, RT-qPCR analysis and quantification of TFEB and target genes (ATP6V1A, NPC2, LAMP1) in aortic macrophages sorted from abdominal aortic tissues of CaPO4 treated 10-week-old C57BL/6J mice with water or naringenin (50 mg/kg/day) gavage (10 mice per group) for 7 days. The data were analyzed using unpaired two-tailed Student's t-test and are presented as the mean ± SEM of six independent experiments. *P<0.05. (r) Representative

immunofluorescence images of IgG staining in human aneurysmal abdominal aortas and non-aneurysmal abdominal aortas.

Supplementary Fig. S3. (a) Schematic view of the generation of TFEB$^{flox/flox}$ mice. (b) Genotyping of the TFEB macrophage-specific knockout mice by PCR analysis. (c) Western blotting analysis of TFEB in the liver, heart, brain, aorta and macrophages from the TFEB$^{flox/flox}$ and macrophage TFEB knockout mice (4 months, male; n=5 mice per genotype). The data were analyzed using two-way ANOVA followed by Bonferroni test for post hoc comparison and are presented as the mean ± SEM. *$P<0.05$. (d) RT-qPCR analysis of TFEB levels in the peritoneal macrophages isolated from mice injected with AAV2-GFP (1×10^{11} genomic copies per mouse) or AAV2-TFEB (1×10^{11} genomic copies per mouse) at day 7. The data were analyzed using unpaired two-tailed Student's *t*-test and are presented as the mean ± SEM. n=3 for each group. *$P<0.05$. (e) Representative photography (left) and maximal infrarenal abdominal aortic diameter quantification (right) of the macroscopic features of $CaPO_4$-induced aneurysms. 12-week-old male C57BL/6J mice injected with AAV2-GFP (1×10^{11} genomic copies per mouse) or AAV2-TFEB (1×10^{11} genomic copies per mouse) were periadventitially treated with $CaPO_4$ for 7 days. n=9-10 for each group. The data were analyzed using unpaired two-tailed Student's *t*-test and are presented as the mean ± SEM. *$P<0.05$. (f) Representative elastin Van Gieson staining and elastin grade quantification of the infrarenal abdominal aortas in panel D. The data were analysis by nonparemetric Kruskal-wallis test with a dumn post hoc test. (g) Quantification of the maximal abdominal aortic diameter of the macrophage TFEB knockout mice with $CaPO_4$-induced AAA before treated by water or naringenin gavage. The data were

analyzed using unpaired *t*-tests and are presented as the mean ± SEM. n=4-5 for each group, ns, no significance.

Supplementary Fig. S4. (a) Lysates of the primary peritoneal macrophages treated with vehicle or naringenin (200 µM, 12 h) were immunoprecipitated with anti-FOXO4 antibody, and the precipitates were analyzed by immunoblotting with anti-14-3-3ε antibody. (b) Diagrammatic view of the incorrectly digested peptide of 14-3-3ε (from E162 to R171) upon naringenin incubation. Red arrows indicate the cleavage sites of trypsin. The blue arrow indicates the missed cleavage site of proteinase K. (c) Titration of p-TFEB peptide (LVGVTSSpSCPADLTQ) and p-control peptide (MARSHpSYPAKKK) with 14-3-3ε. The probe concentration was 10 nM in 5% DMSO (v/v). Data are presented as the mean ± SEM of three independent experiments. (d) Results of the competitive binding assay for naringenin of p-control peptide (MARSHpSYPAKKK) and 14-3-3ε. The peptide concentration was 5 nM, and the concentration of 14-3-3ε was 10 nM. Data are presented as the mean ± SEM of three independent experiments. (e) Nuclear-cytoplasmic separation followed by Western blot analysis of scrambled siRNA- or 14-3-3 epsilon siRNA (50 nM, 48 hours)-transfected primary peritoneal macrophages. The data were analyzed using two-way ANOVA followed by Bonferroni test for post hoc comparison and are presented as the mean ± SEM of six independent experiments. **P*<0.05. (f) Primary peritoneal macrophages were transfected with scrambled siRNA (50 nM) or 14-3-3 epsilon siRNA (50 nM) for 48 hours then followed by Lysotracker Red staining for 30 mins and analyzed by flow cytometry. The data were analyzed using unpaired two-tailed Student’s *t*-test and are presented as the mean ± SEM of four

independent experiments. *$P<0.05$. The cells treated same with (f) were incubated with Lysotracker Green (g) or DQ-Red BSA (h) for 30 mins and then analyzed by live cell confocal microscopy. Scale bar = 10 μm.

Supplementary Fig. S5. (a) Representative image (upper) and quantification (lower) of the number of myeloid cells (Ly6C+/CD45+ in bone marrow) by using flow cytometric assays. The data were analyzed using unpaired two-tailed Student's *t*-test and are presented as the mean ± SEM. N=3-5 for each group. (b) Representative image (upper) and quantification (lower) of the number of monocytes (Ly6C^{high} in CD45+CD11b+Ly6G- blood cells) by using flow cytometric assays. The data were analyzed using unpaired two-tailed Student's *t*-test and are presented as the mean ± SEM. n=3-5 for each group. (c) RT-qPCR validation of IL-1β, TNF-α, iNOS and MCP1 in the wild-type (WT, TFEB$^{flox/flox}$ macrophages infected with Ad-GFP), TFEB knockout (TFEB KO, TFEB$^{flox/flox}$ macrophages infected with Ad-Cre) and TFEB-overexpressing (TFEB OE, TFEB$^{flox/flox}$ macrophages infected with Ad-TFEB) macrophages treated with vehicle or LPS (10 ng/ml, 12 h). The data were analyzed using two-way ANOVA followed by Bonferroni test for post hoc comparison and are presented as the mean ± SEM of six independent experiments. *$P<0.05$. (d) RT-qPCR validation of IRF9, KLF6, IRF5, RBP-J and STAT1 in the wild-type (WT) and TFEB knockout (TFEB KO) macrophages. The data were analyzed using unpaired two-tailed Student's *t*-test and are presented as the mean ± SEM of six independent experiments. *$P<0.05$. (e) RT-qPCR validation of KLF4, KLF2, EGR2, CMAF and STAT3 in the wild-type (WT) and TFEB knockout (TFEB KO) macrophages. The data were analyzed using unpaired two-tailed Student's *t*-test and are presented as the mean ±

SEM of six independent experiments. **P*<0.05. (f) RT-qPCR validation of KLF4, KLF2, EGR2, CMAF and STAT3 in the wild-type (WT) and TFEB-overexpressing (TFEB OE) macrophages. The data were analyzed using unpaired two-tailed Student's *t*-test and are presented as the mean ± SEM of six independent experiments. **P*<0.05.

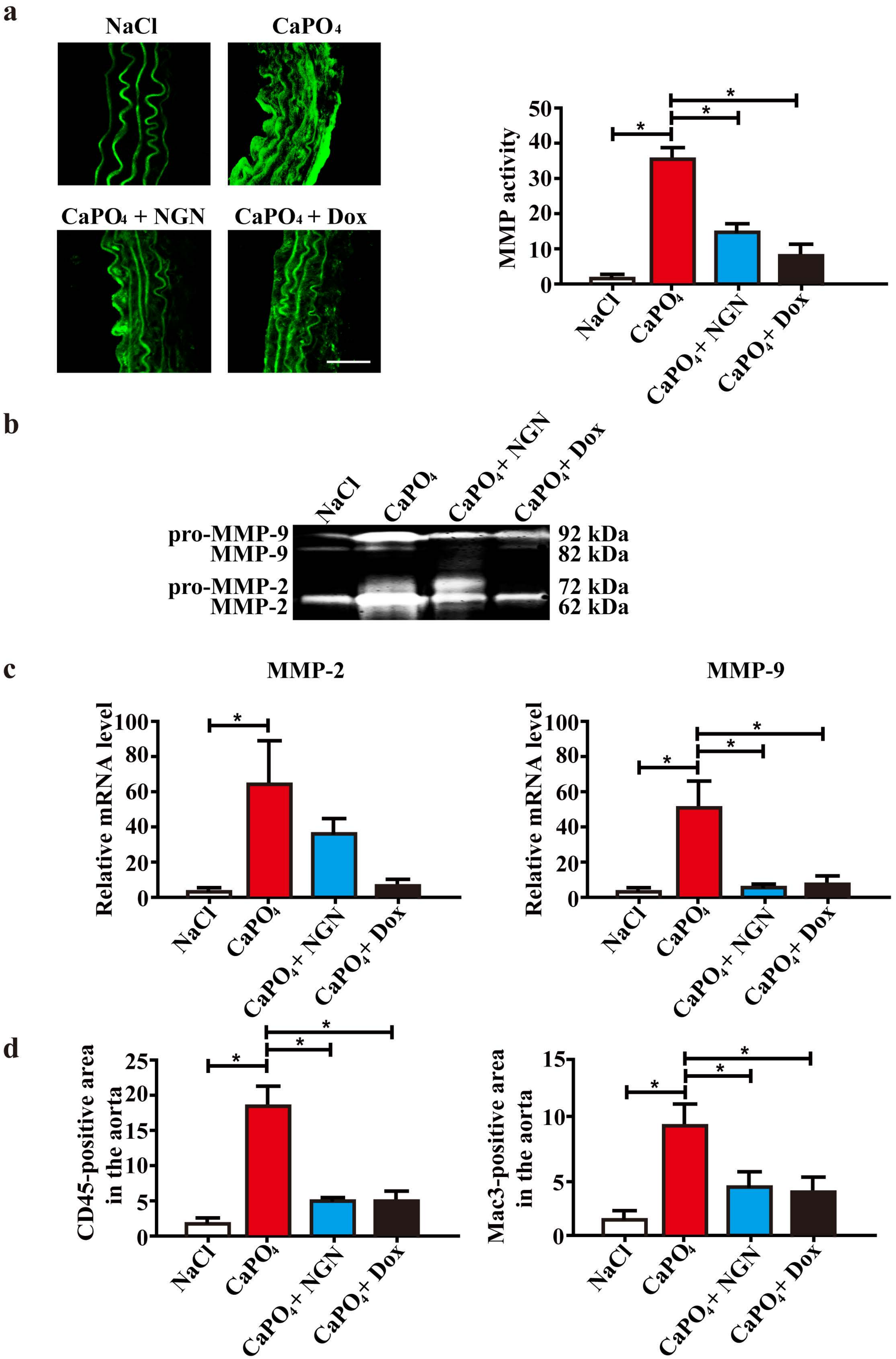

Supplementary Fig.S1

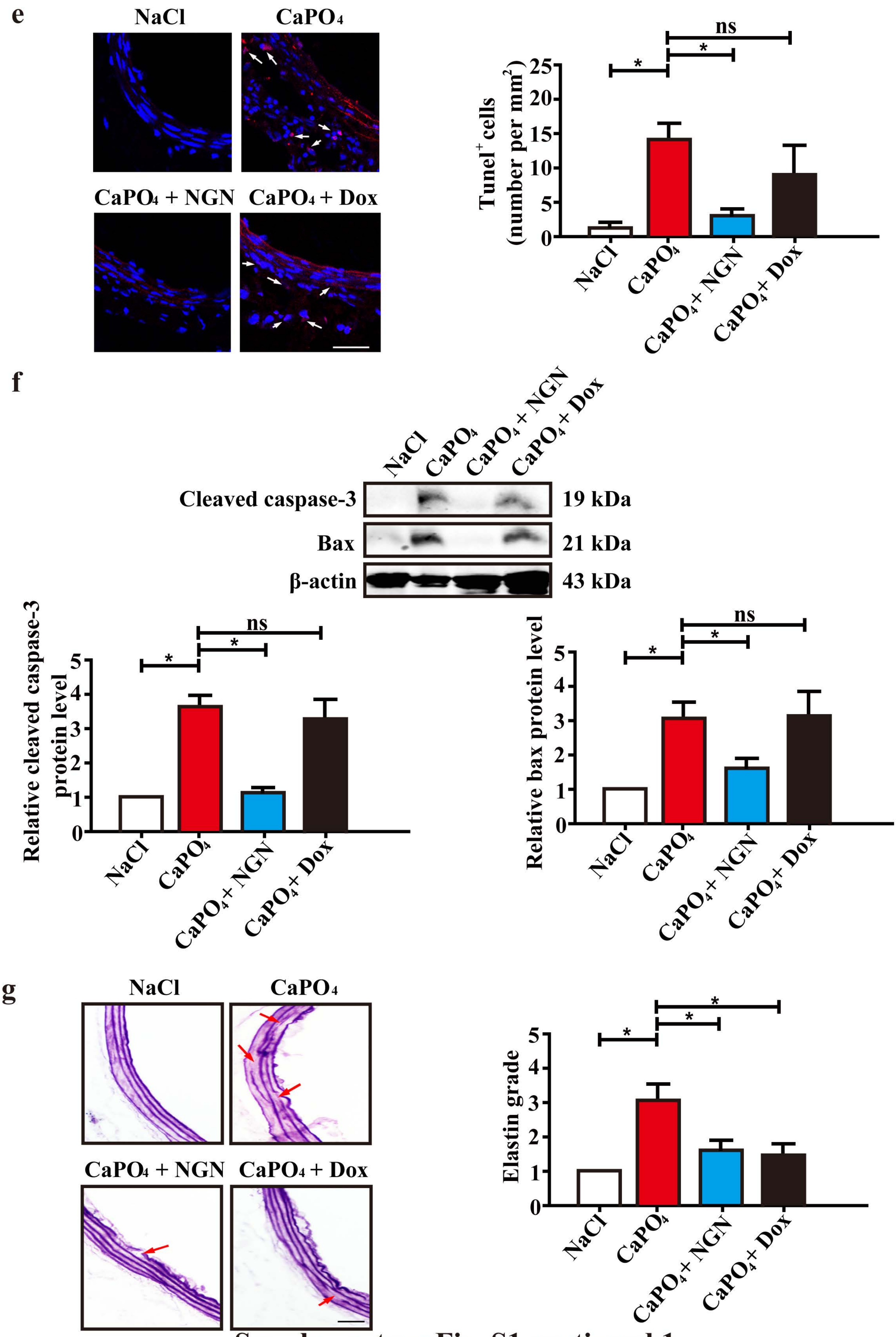

Supplementary Fig. S1 continued 1

h

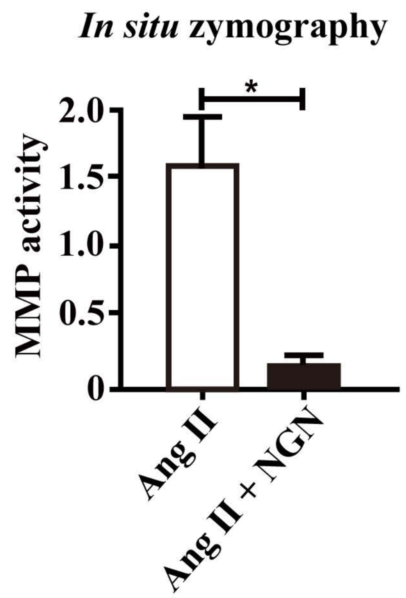

i

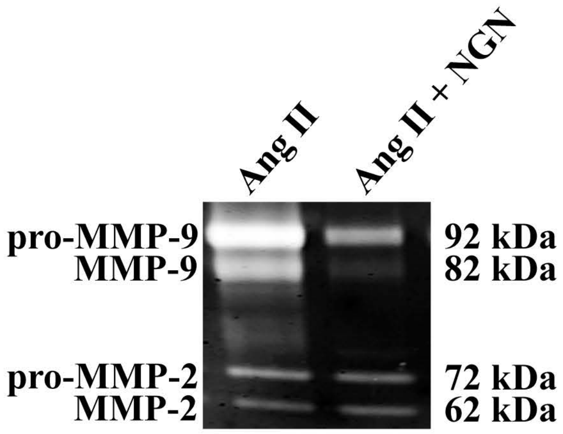

j

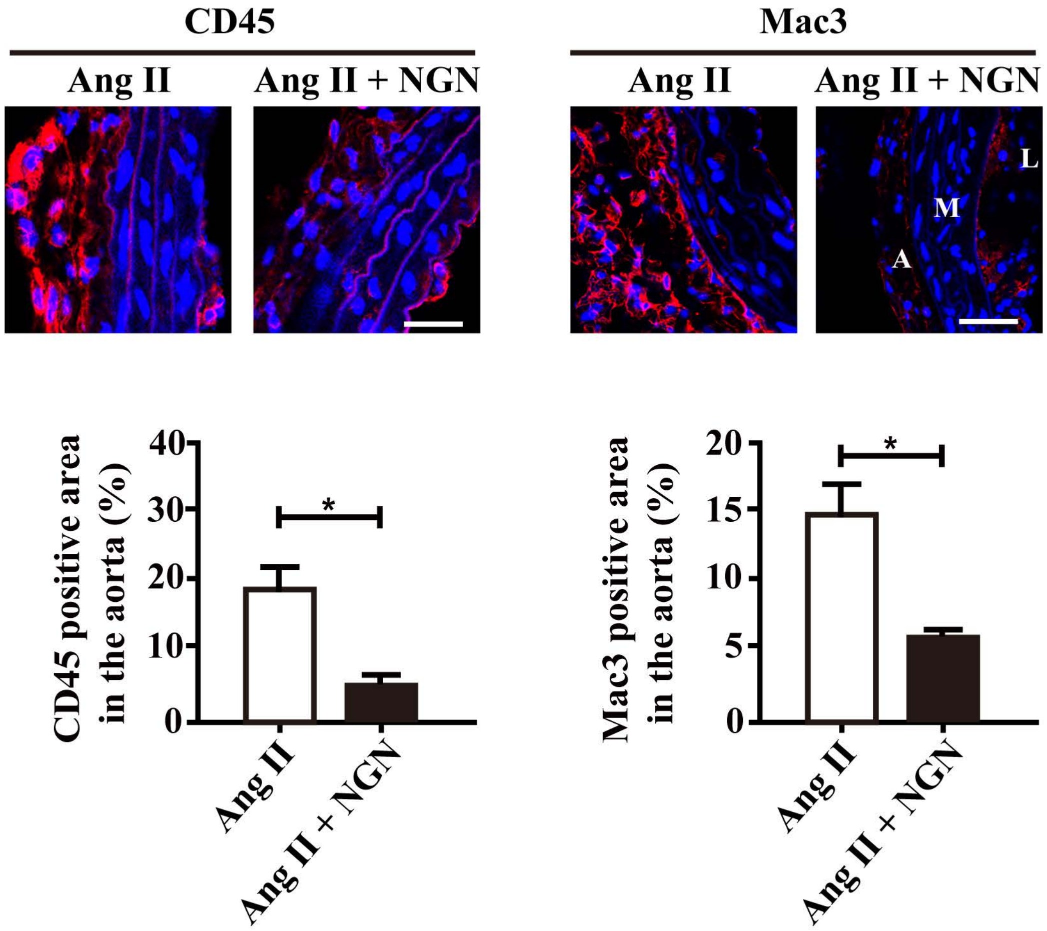

k

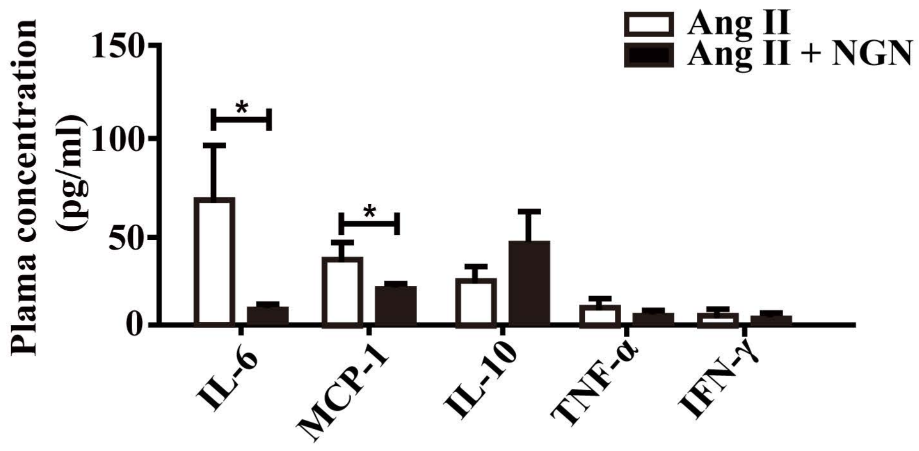

Supplementary Fig. S1 continued 2

l

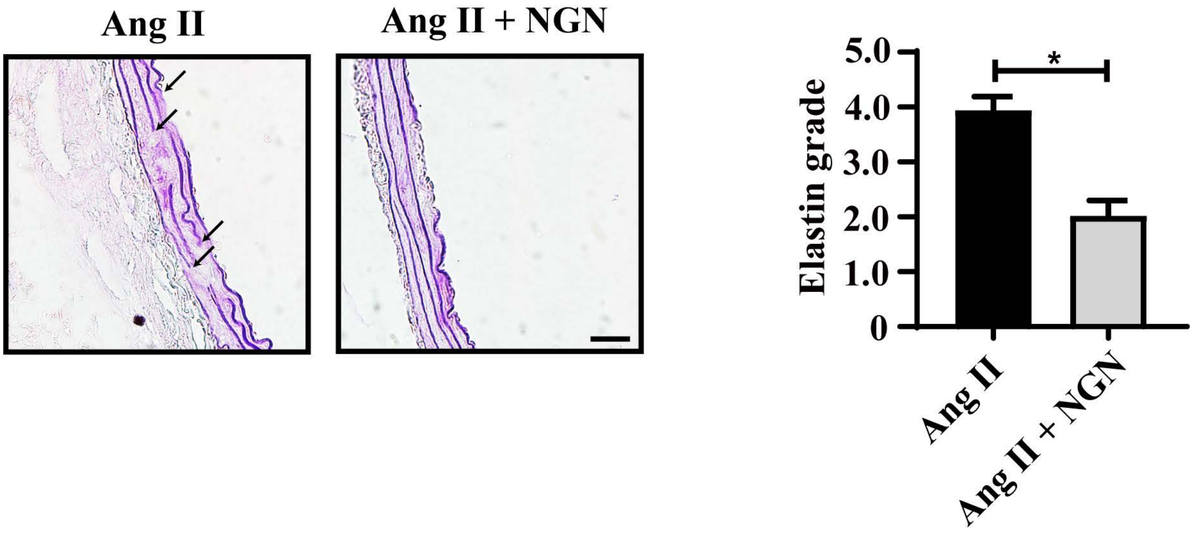

Supplementary Fig. S1 continued 3

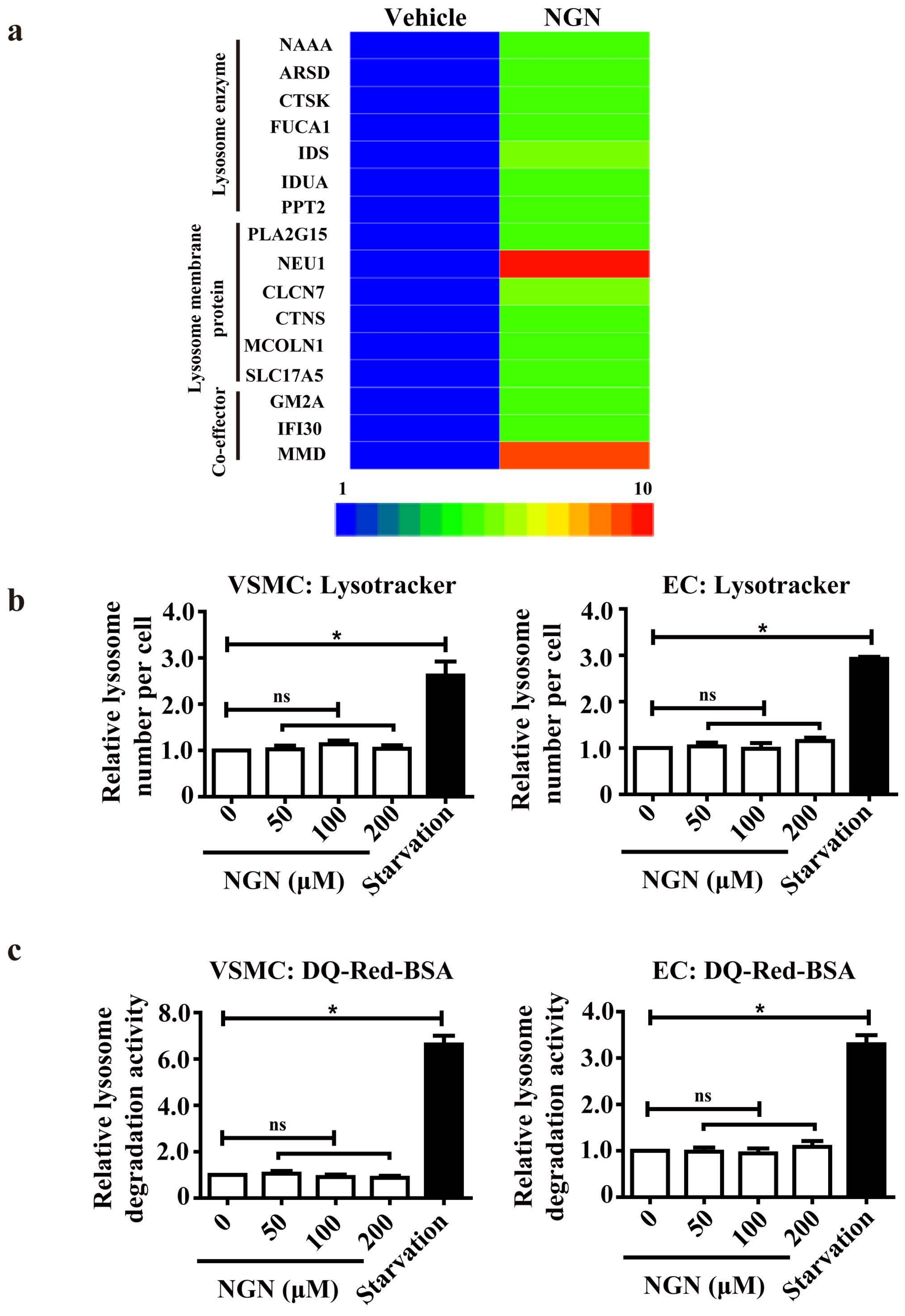

Supplementary Fig. S2

d

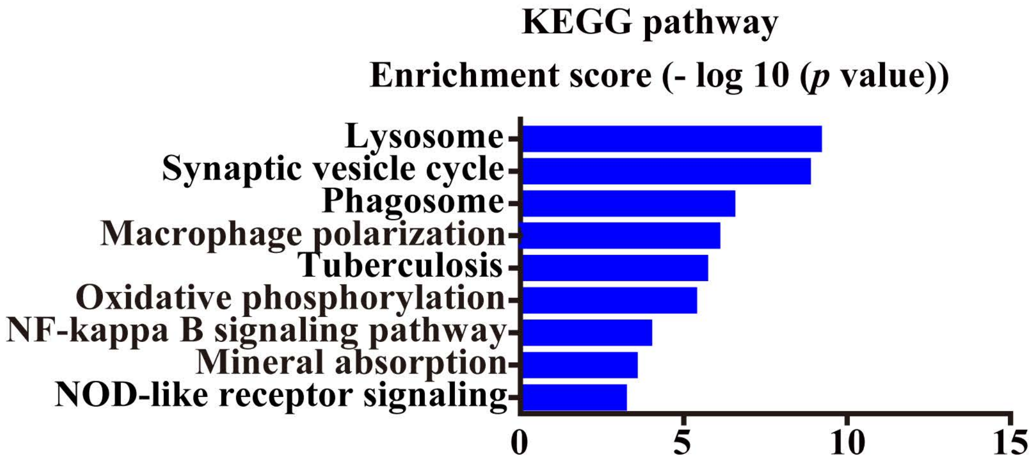

e

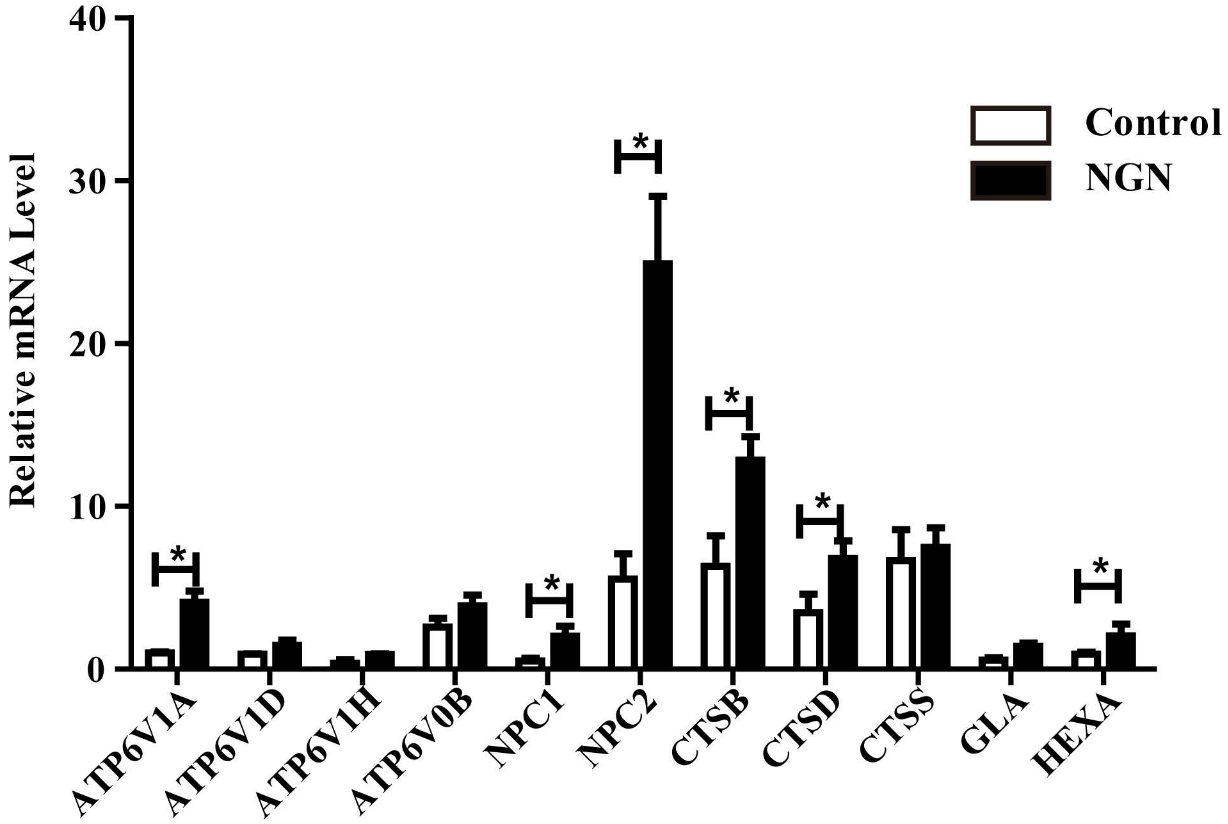

f

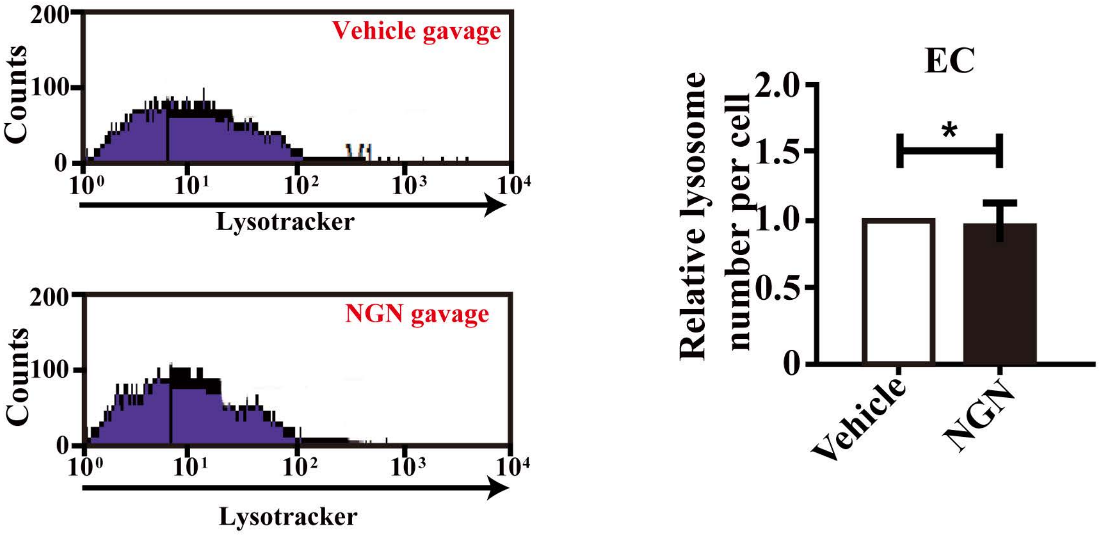

Supplementary Fig. S2 continued 1

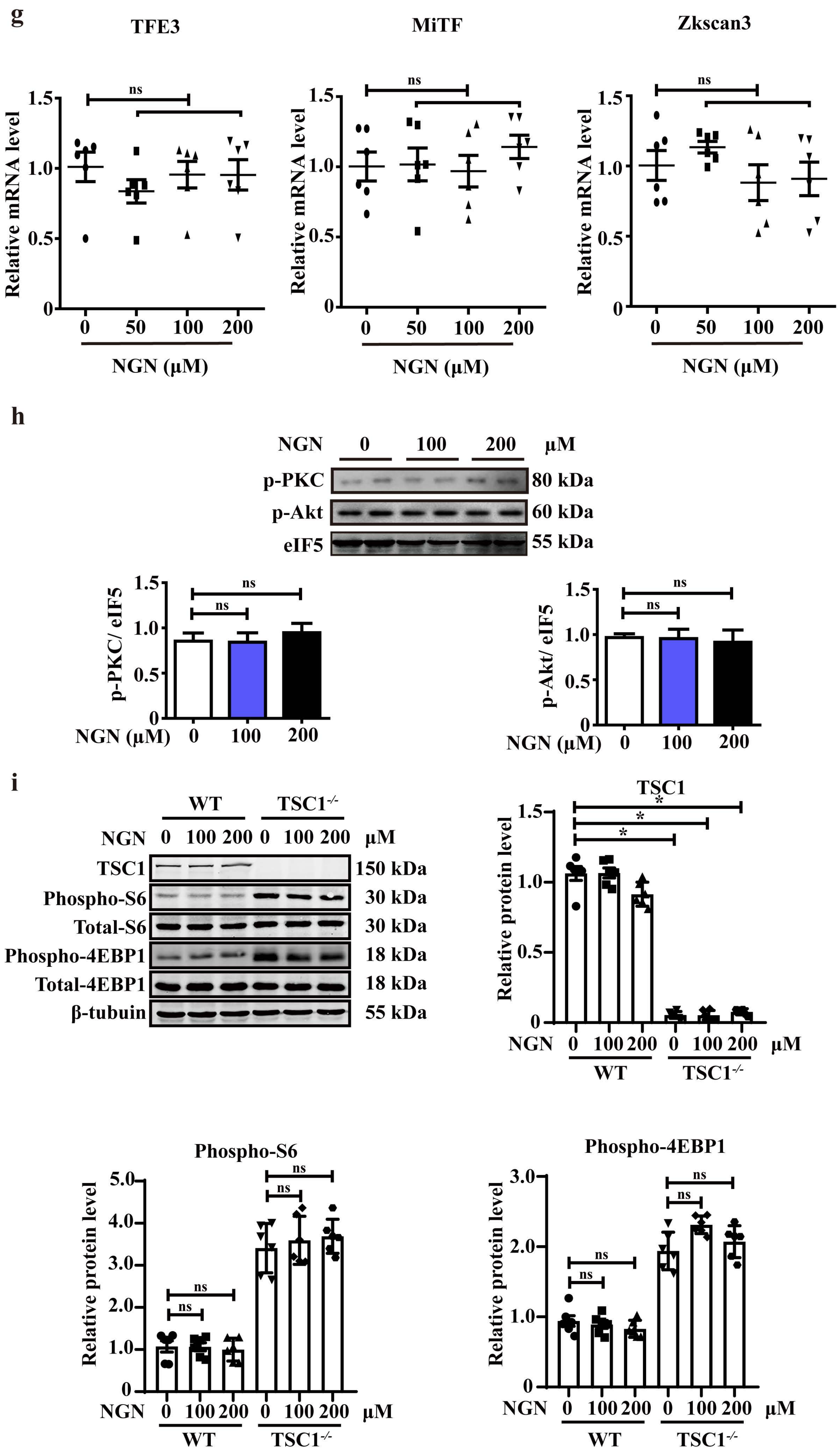

Supplementary Fig.S2 continued 2

j

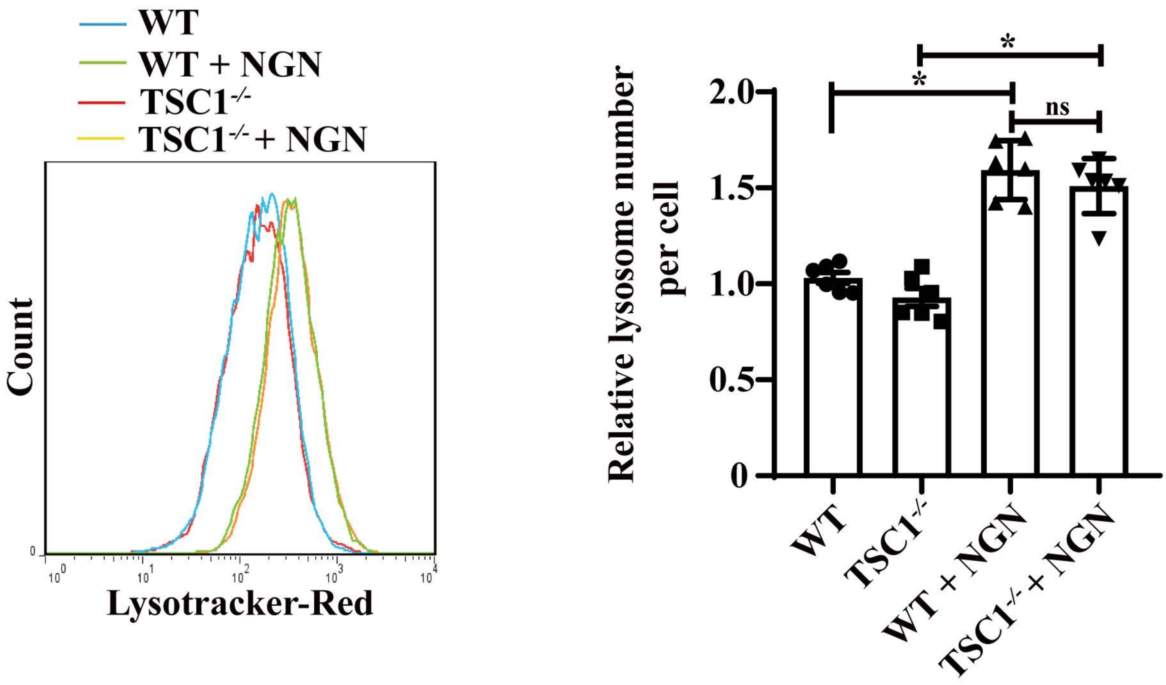

k

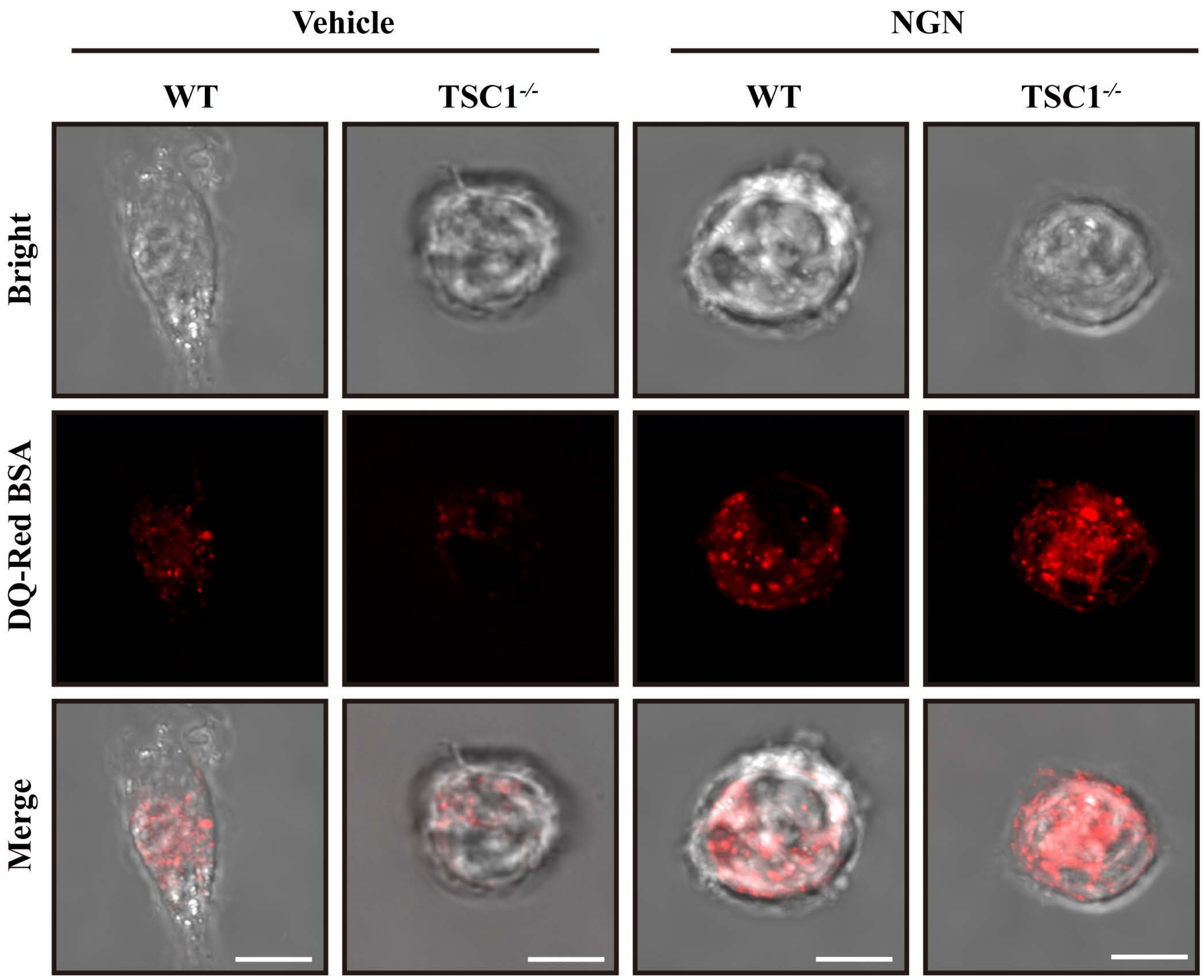

Supplementary Fig.S2 continued 3

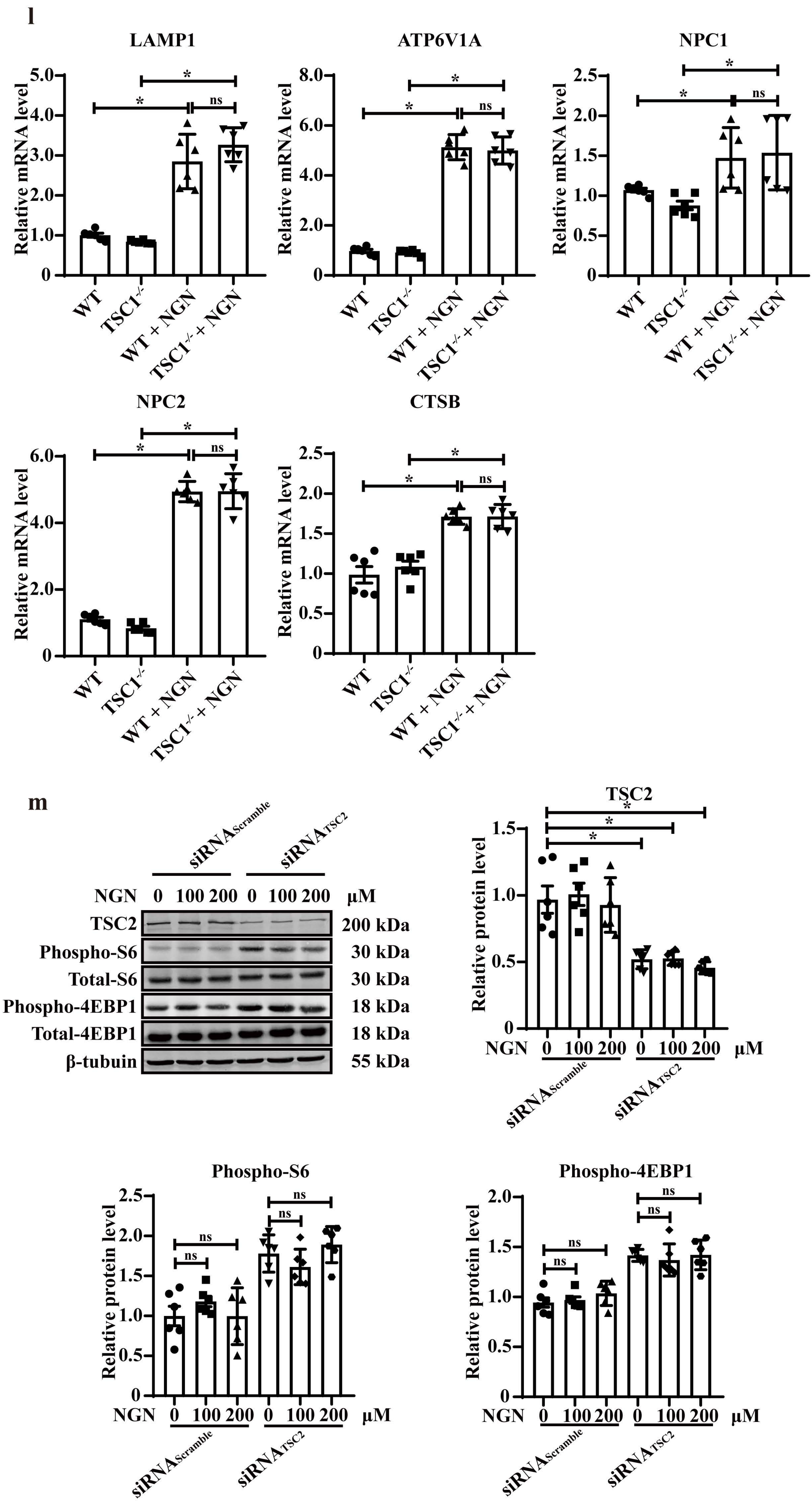

Supplementary Fig.S2 continued 4

n

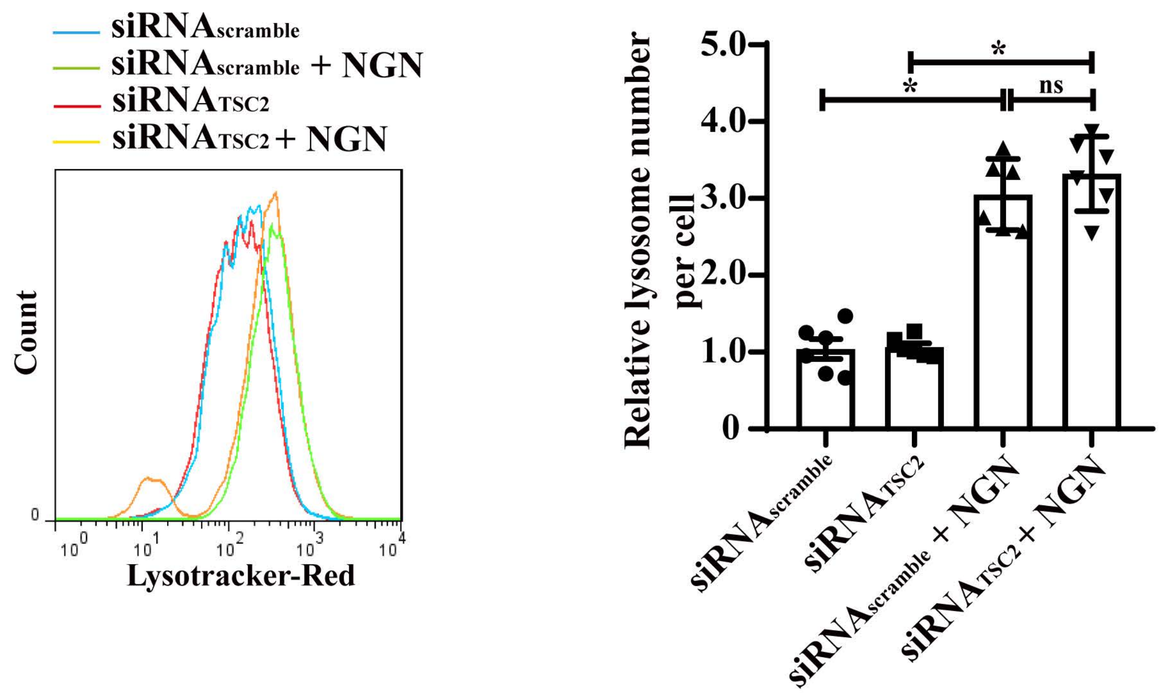

o

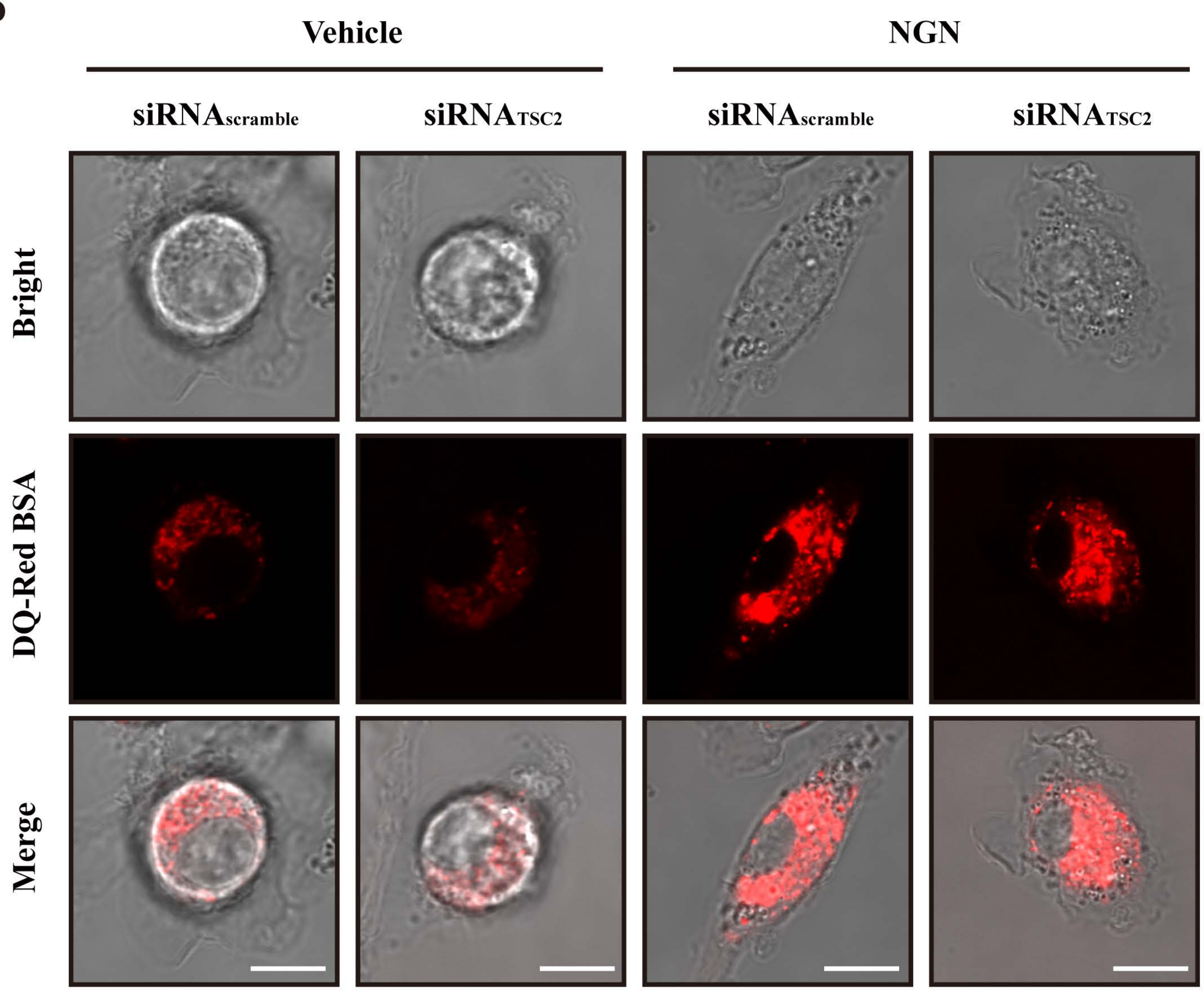

Supplementary Fig.S2 continued 5

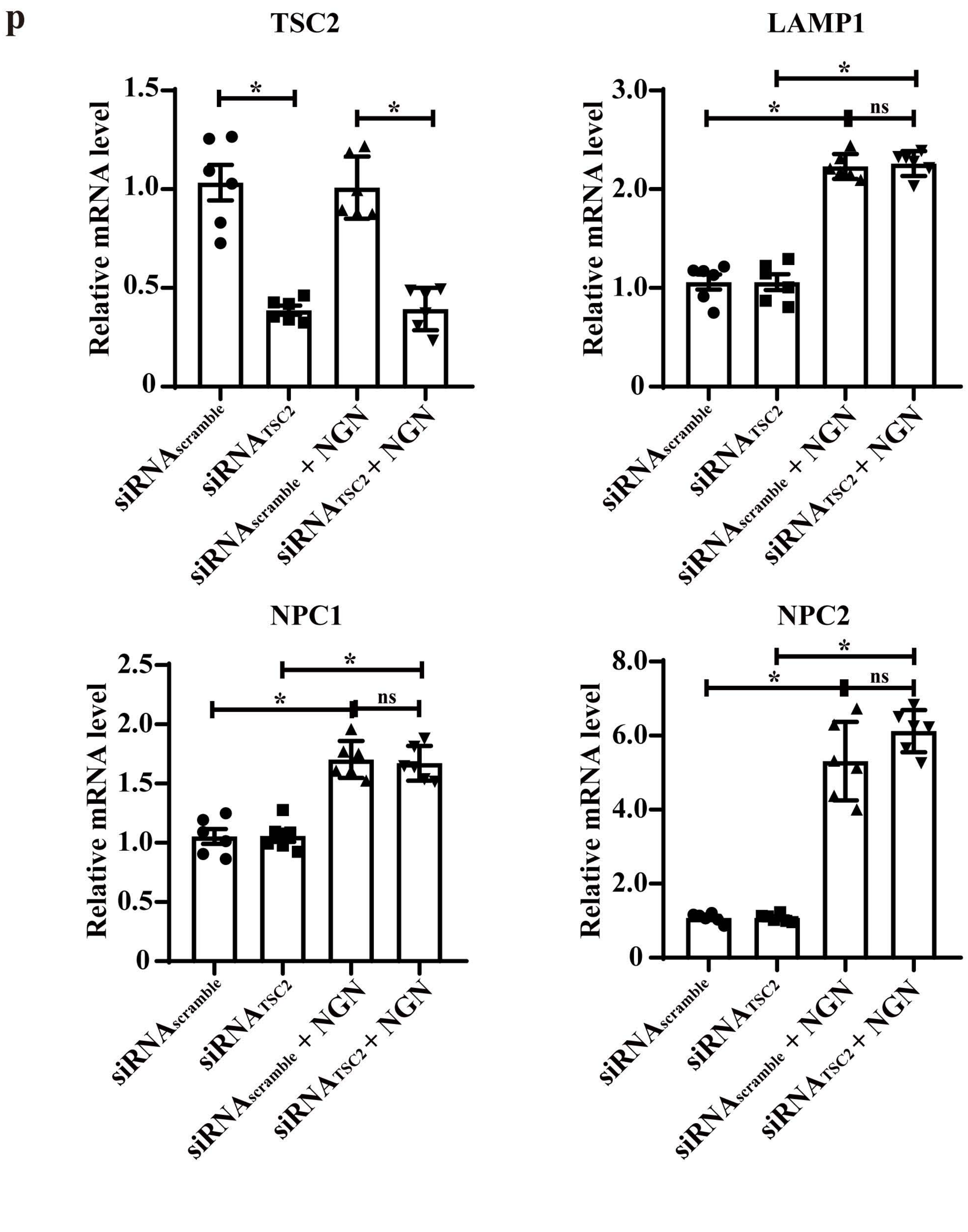

Supplementary Fig.S2 continued 6

q

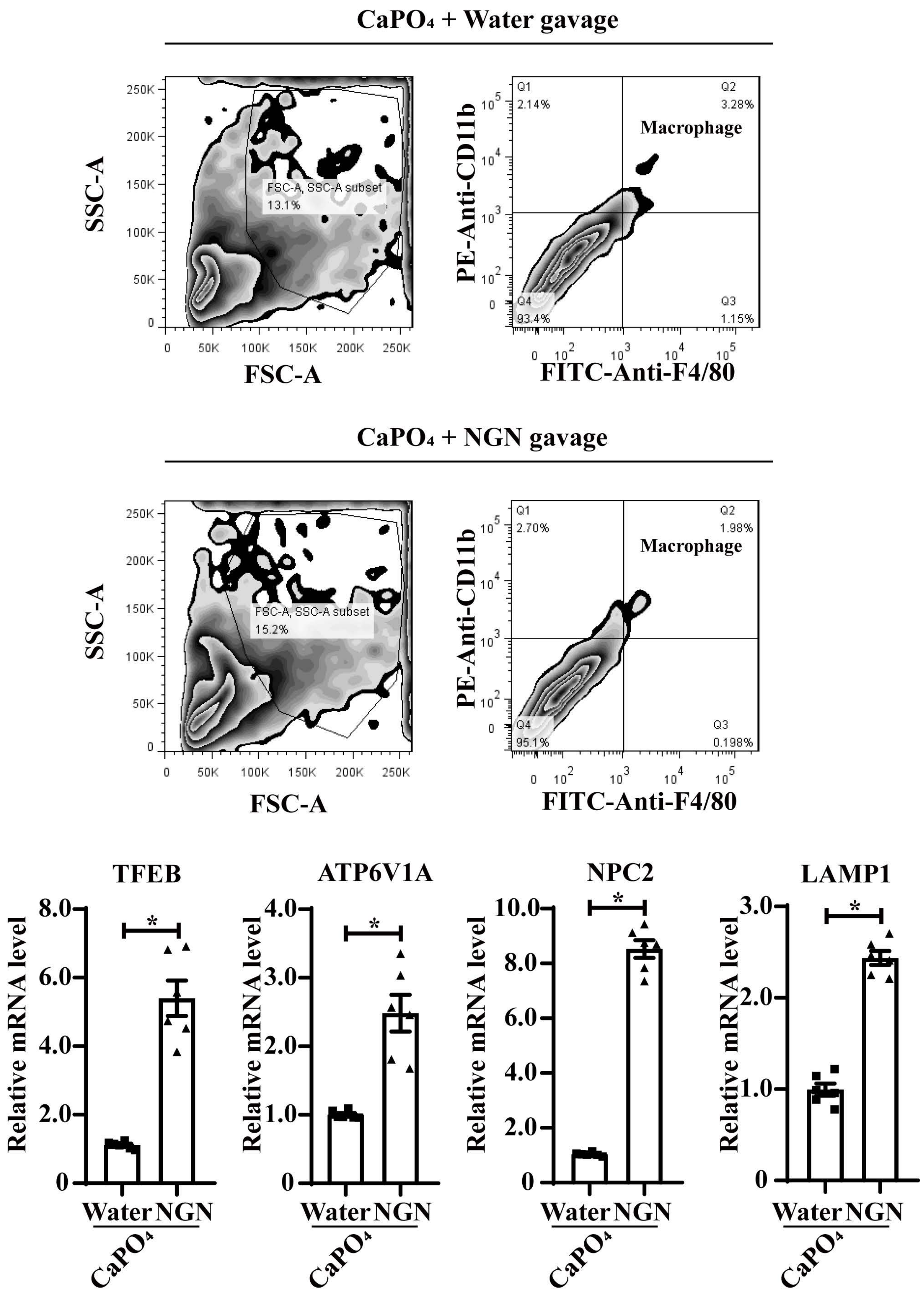

r

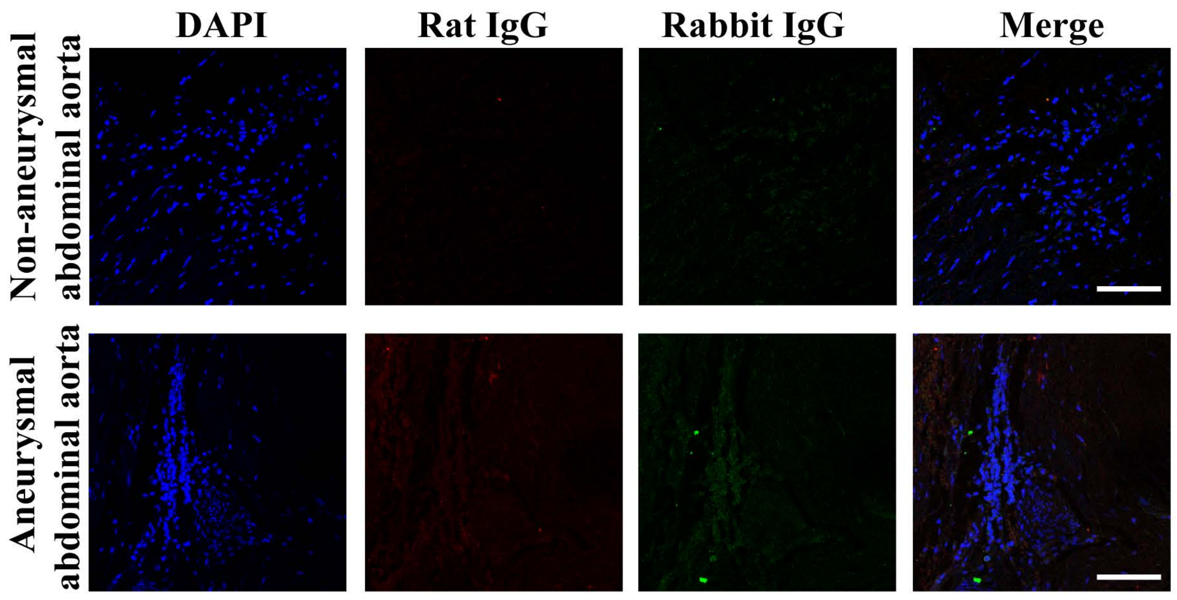

Supplementary Fig.S2 continued 7

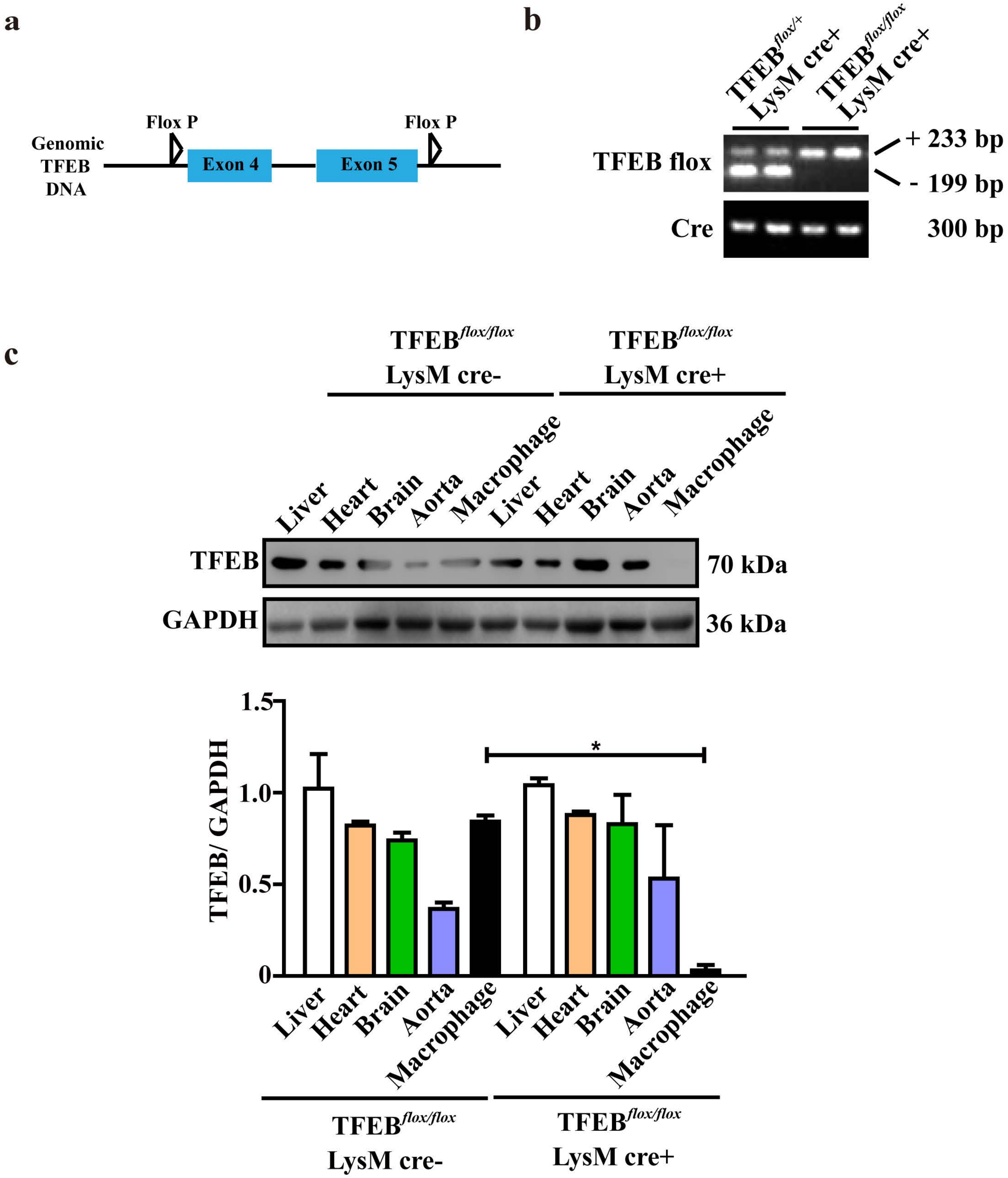

Supplementary Fig.S3

d

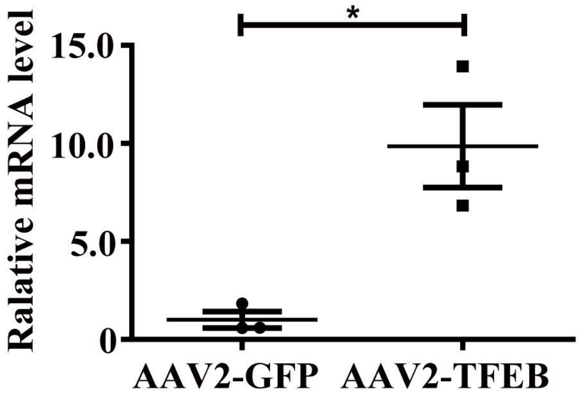

e

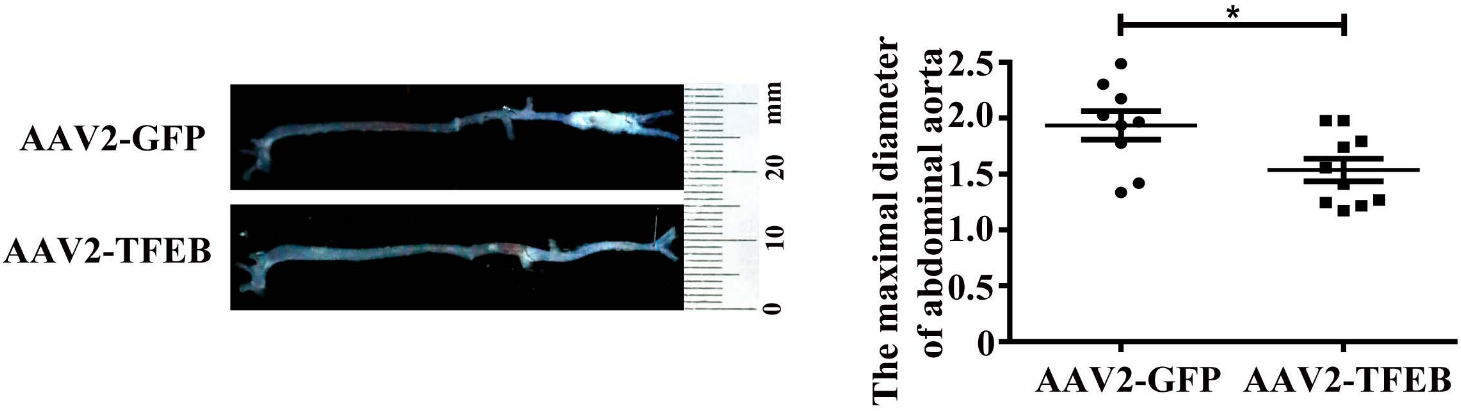

f

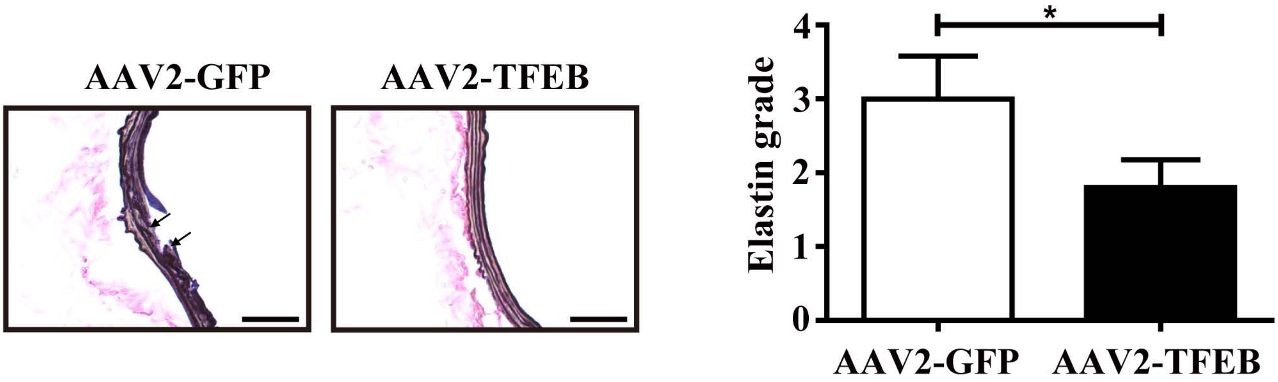

g

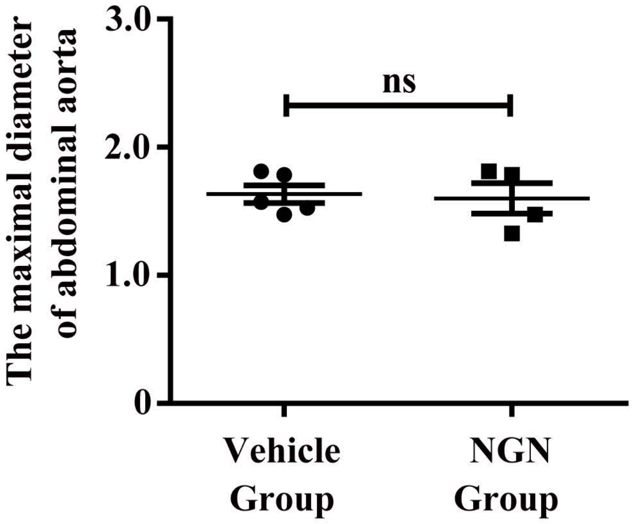

Supplementary Fig.S3 continued

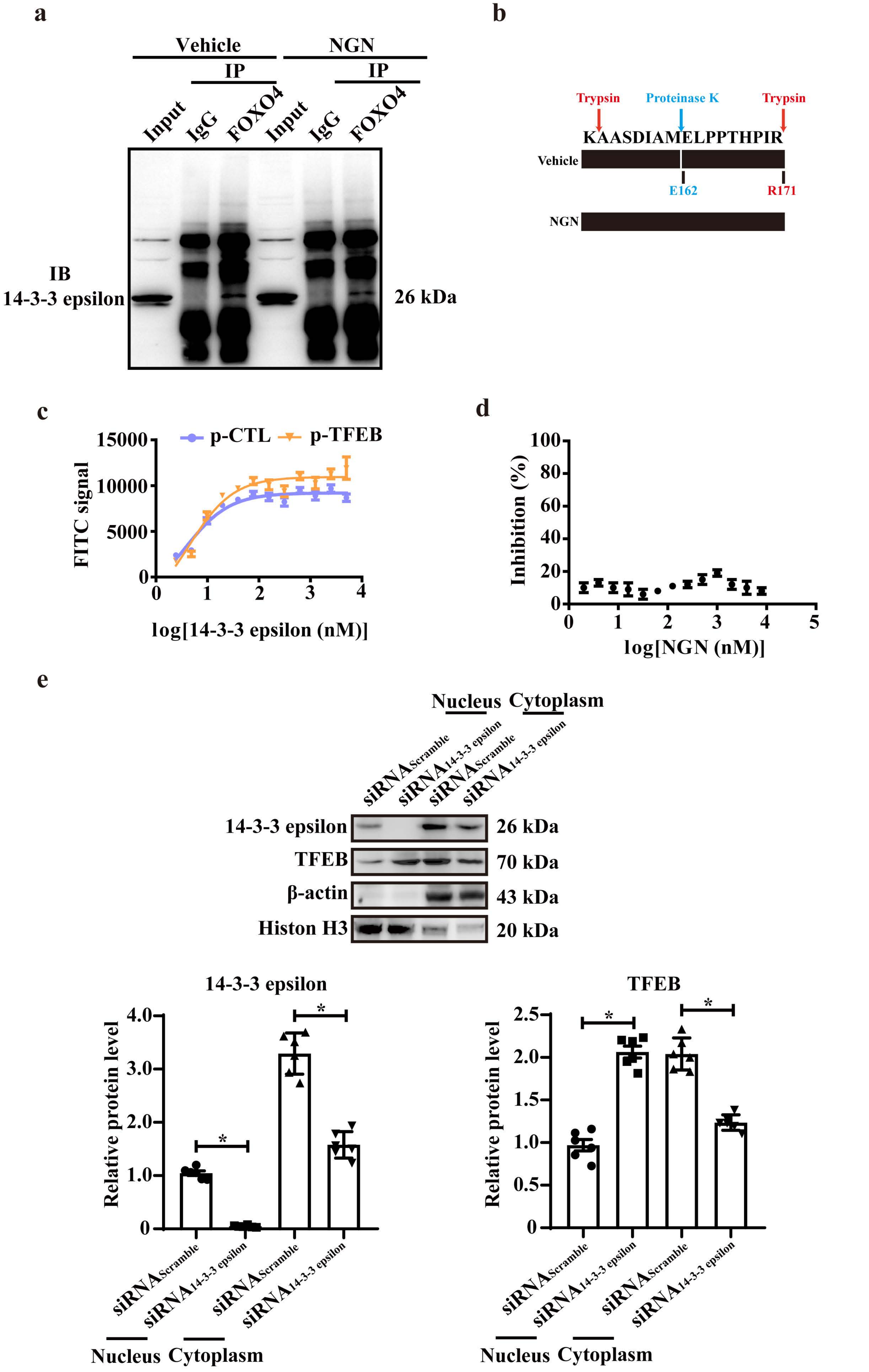

Supplementary Fig.S4

f

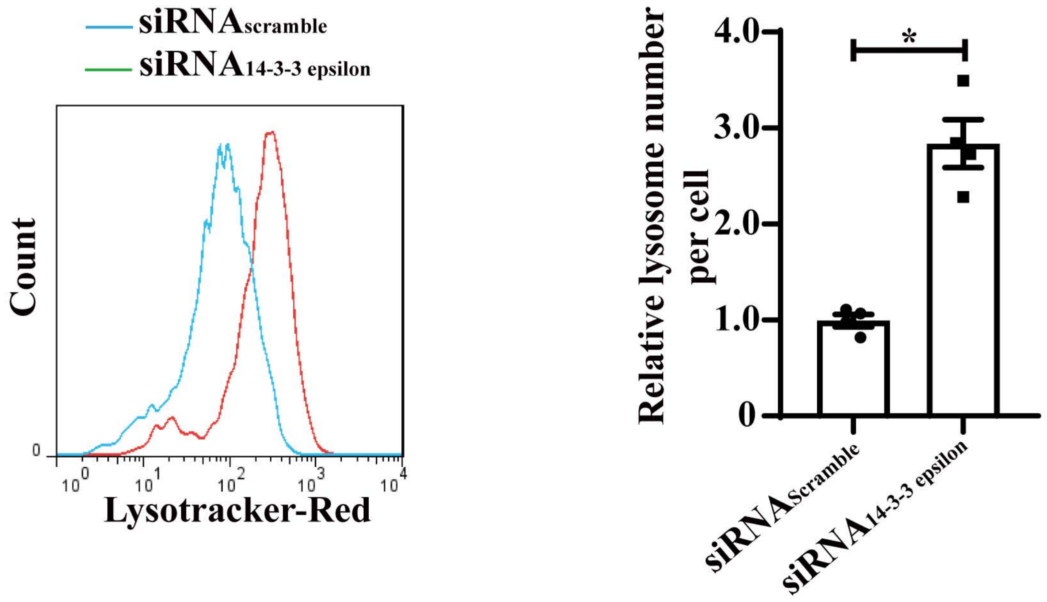

g

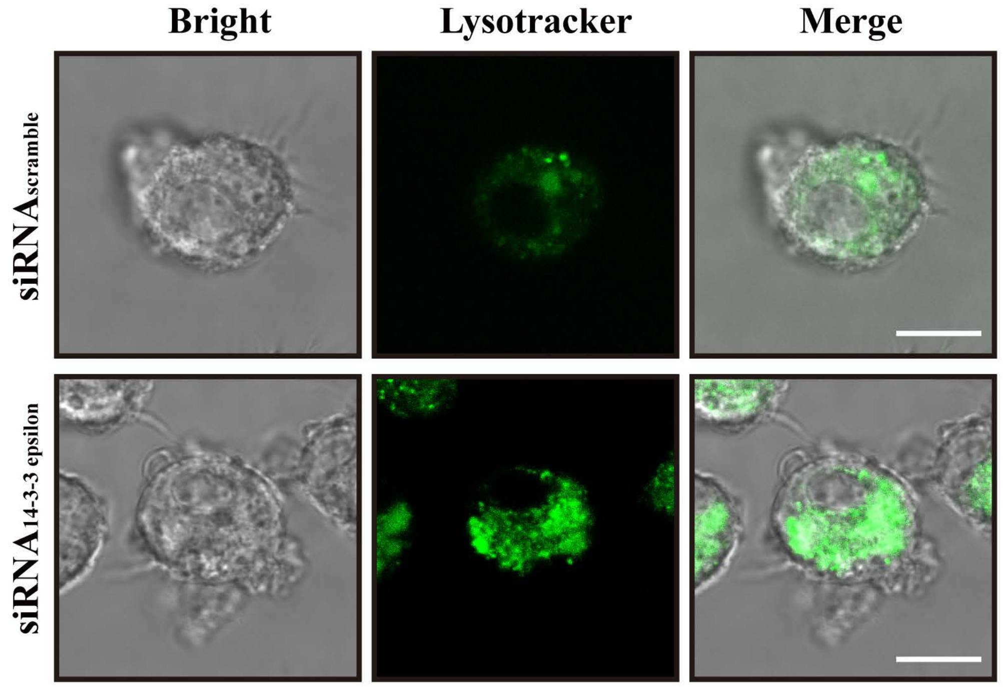

h

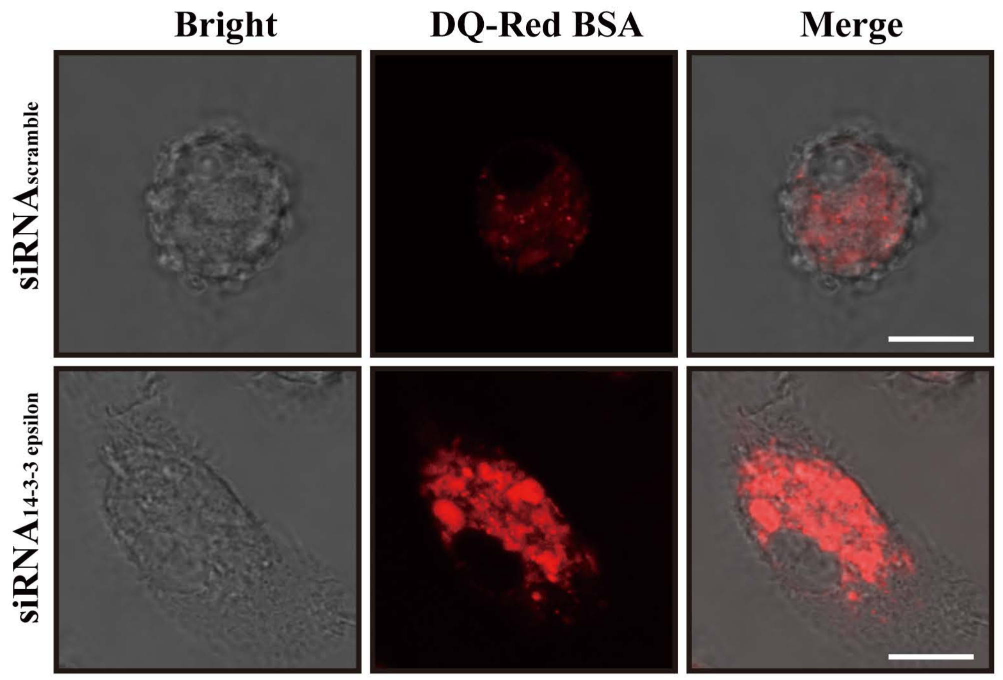

Supplementary Fig.S4 continued

a

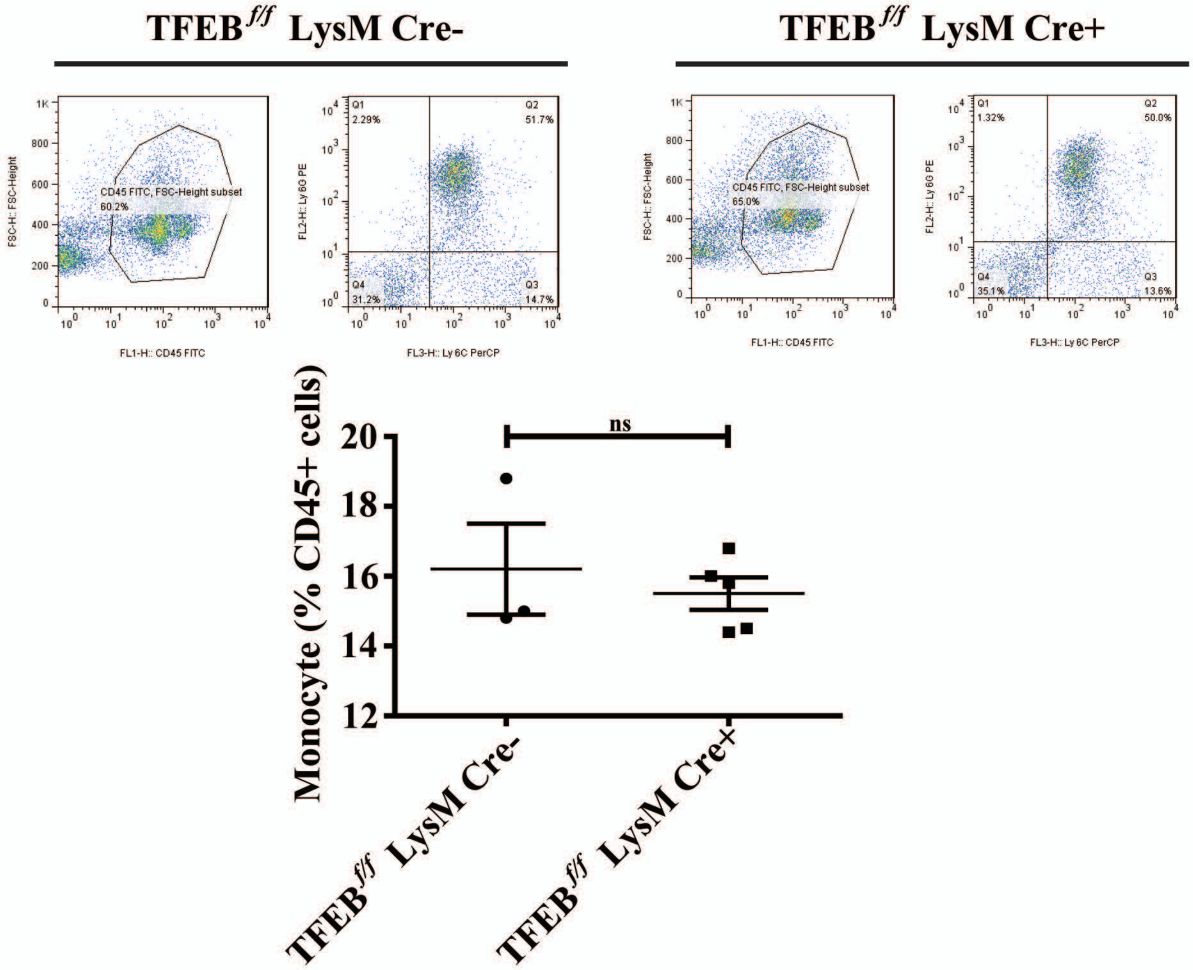

b

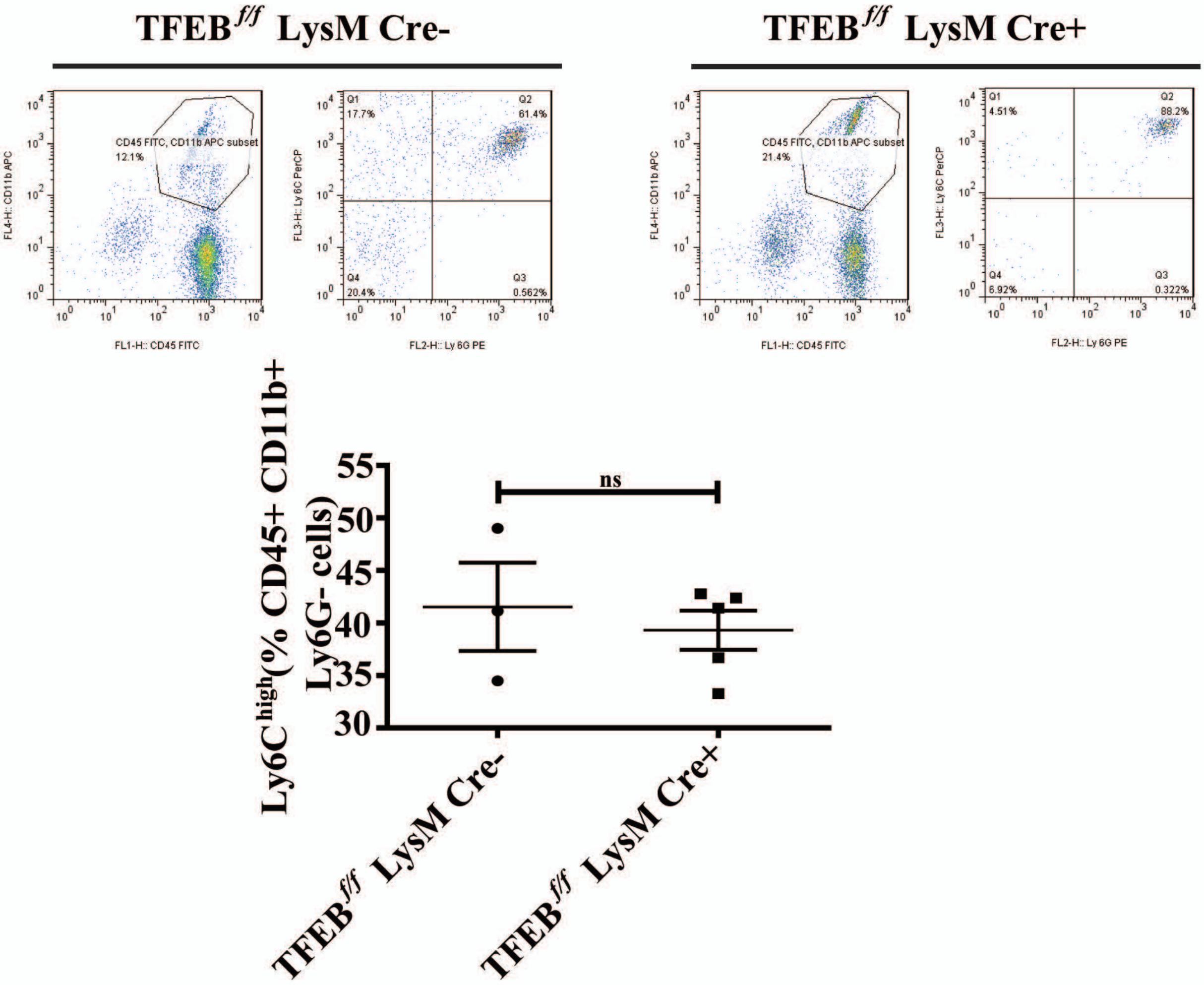

Supplementary Fig.S5

c

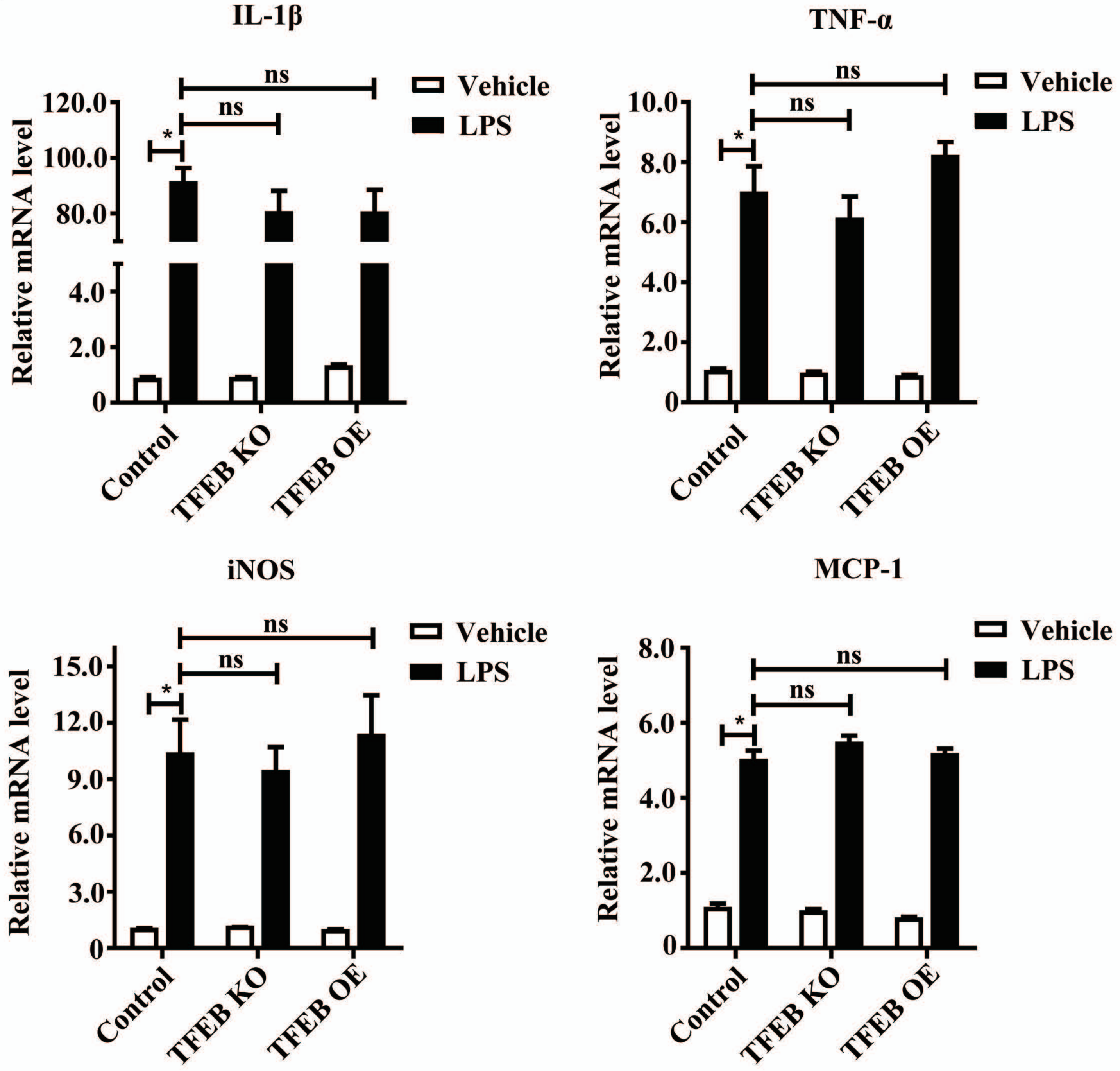

Supplementary Fig.S5 continued 1

d

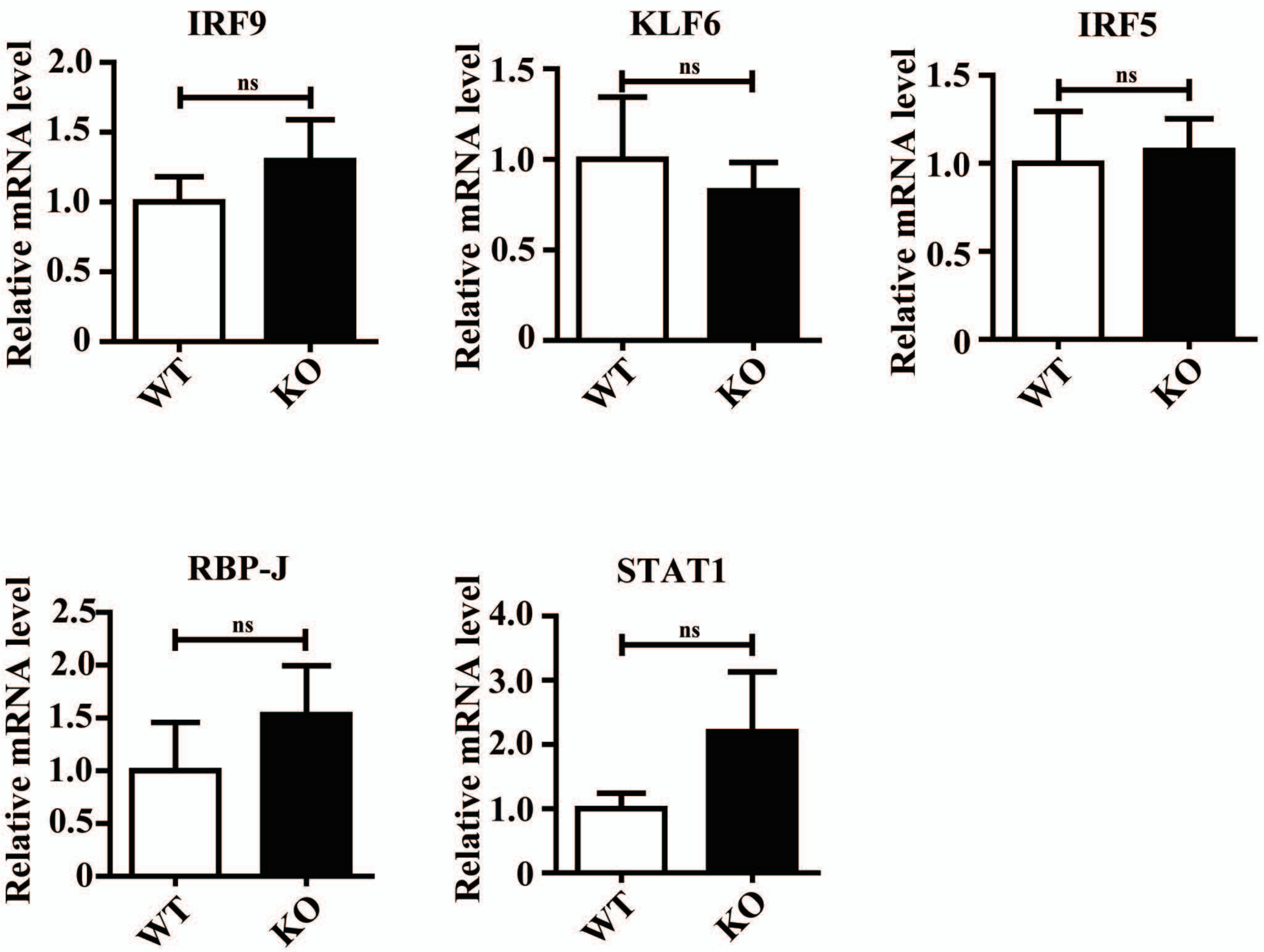

e

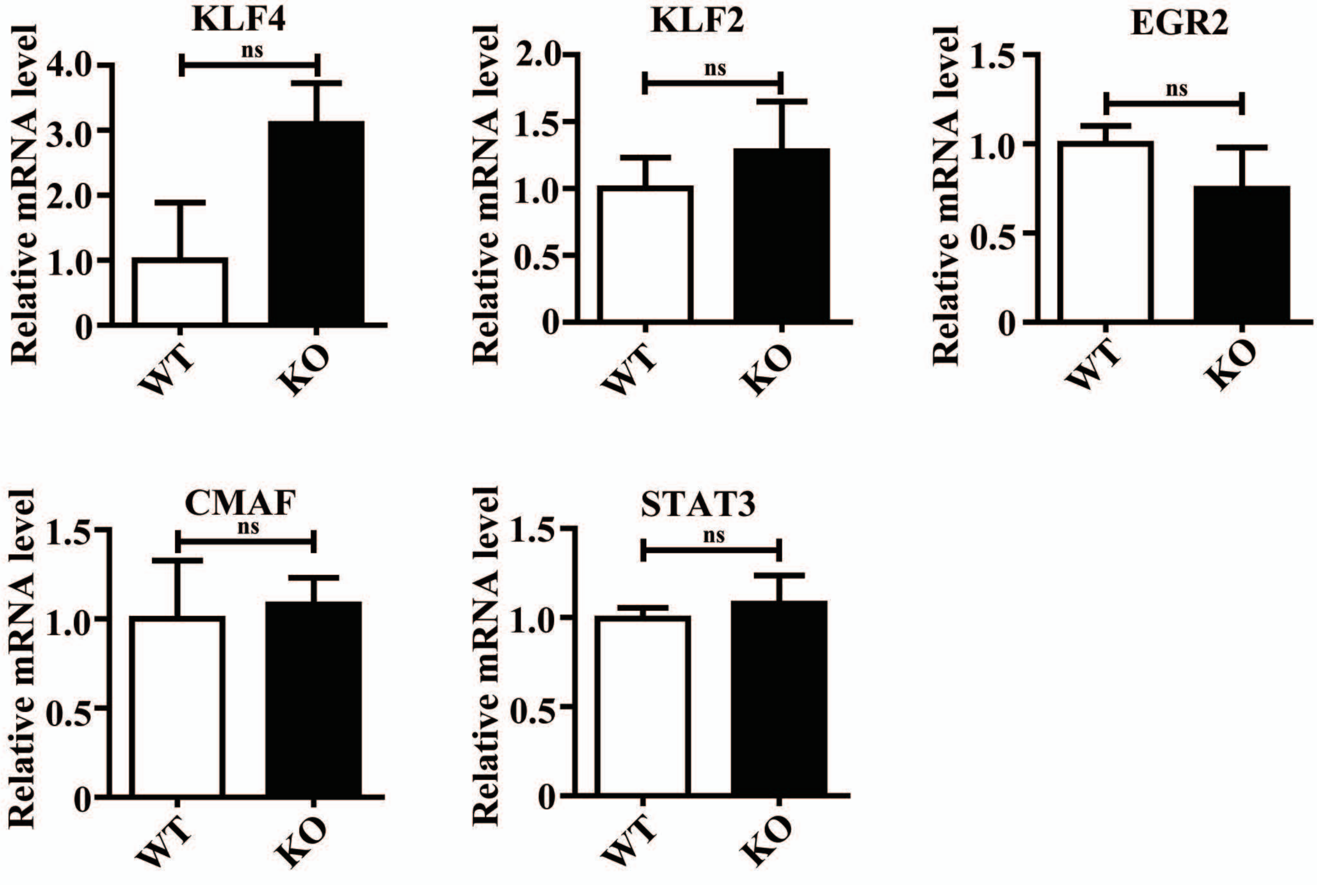

Supplementary Fig.S5 continued 2

f

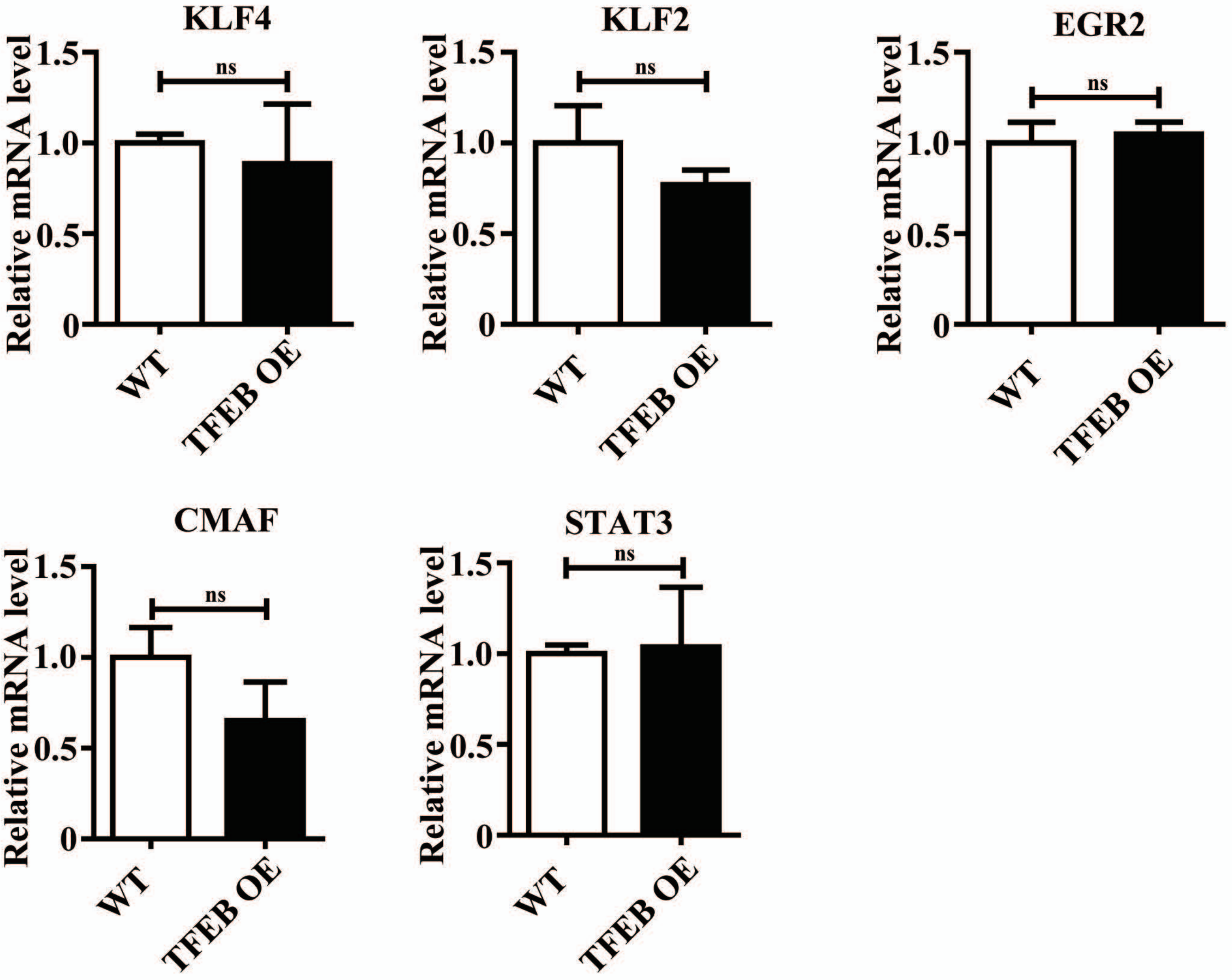

Supplementary Fig.S5 continued 3

Supplementary Table S1. List of drugs in the Connectivity Map and their correlation with doxycycline in drug-responsive expression profiles.

Drug	correlation coefficient	*P* -value
iopamidol	0.323148	0
sulfamonomethoxine	0.2944964	0
DL-PPMP	0.2875107	0
ketotifen	0.2575437	0
podophyllotoxin	0.246211	5.96E-305
methyldopate	0.2448588	1.57E-301
streptomycin	0.2422756	4.72E-295
calcium folinate	0.2413125	1.17E-292
naringenin	0.2363441	1.80E-280
zuclopenthixol	0.2361292	5.96E-280
tolnaftate	0.2329646	2.41E-272
methoxamine	0.2322435	1.26E-270
sulfadimethoxine	0.2304645	2.04E-266
dapsone	0.2299272	3.76E-265
ambroxol	0.2289667	6.73E-263
lisuride	0.2288862	1.04E-262
SB-203580	0.2287643	2.00E-262
fluticasone	0.2278678	2.47E-260
Prestwick-691	0.2248227	2.68E-253
xamoterol	0.2242593	5.22E-252
bumetanide	0.2240391	1.67E-251
levopropoxyphene	0.2228707	7.64E-249
benzathine benzylpenicillin	0.2224484	6.93E-248
betulinic acid	0.2207098	5.82E-244
bacampicillin	0.2197709	7.40E-242
pyrithyldione	0.2194513	3.83E-241
alprostadil	0.2189723	4.49E-240
adiphenine	0.2188048	1.06E-239
nadolol	0.2182309	2.00E-238
3-acetamidocoumarin	0.2155338	1.76E-232
yohimbic acid	0.2101305	8.18E-221
canadine	0.2093337	4.03E-219
decamethonium bromide	0.2091902	8.13E-219
myricetin	0.2087803	5.99E-218
terazosin	0.2079325	3.68E-216
naproxen	0.2063948	6.17E-213
genistein	0.2045541	4.12E-209
benzocaine	0.2026339	3.68E-205
zalcitabine	0.2012554	2.38E-202
ceforanide	0.2010863	5.25E-202

trimethobenzamide	0.2007786	2.21E-201
tetracycline	0.1965853	5.58E-193
folic acid	0.1964884	8.69E-193
merbromin	0.1955401	6.48E-191
ajmaline	0.1951418	3.94E-190
Gly-His-Lys	0.1948474	1.49E-189
diloxanide	0.1940869	4.59E-188
viomycin	0.1936947	2.68E-187
monensin	0.193324	1.41E-186
heptaminol	0.1924032	8.64E-185
bemegride	0.1920649	3.90E-184
iohexol	0.1917371	1.67E-183
pheniramine	0.1906388	2.16E-181
clidinium bromide	0.1901039	2.28E-180
cefaclor	0.1887332	9.28E-178
amylocaine	0.1882577	7.38E-177
khellin	0.1879266	3.11E-176
CP-863187	0.1870222	1.57E-174
Prestwick-1082	0.1868699	3.03E-174
harpagoside	0.1867995	4.11E-174
levomepromazine	0.1864793	1.64E-173
clorsulon	0.1858873	2.09E-172
tocainide	0.1856486	5.82E-172
convolamine	0.1839191	9.35E-169
timolol	0.1833588	1.01E-167
finasteride	0.1830816	3.25E-167
metronidazole	0.1826551	1.97E-166
dicloxacillin	0.18253	3.33E-166
diphenhydramine	0.1820917	2.11E-165
baclofen	0.1817398	9.22E-165
12,13-EODE	0.1814188	3.54E-164
streptozocin	0.1804488	2.03E-162
dihydroergocristine	0.179759	3.56E-161
PHA-00745360	0.178741	2.39E-159
lasalocid	0.1762641	5.98E-155
arcaine	0.1762302	6.86E-155
gentamicin	0.1759707	1.96E-154
5186324	0.1759531	2.11E-154
stachydrine	0.1752102	4.25E-153
gabexate	0.1740994	3.69E-151
meteneprost	0.1735381	3.49E-150
amantadine	0.1733328	7.91E-150
dimenhydrinate	0.1732476	1.11E-149

felbinac	0.1729467	3.68E-149
neostigmine bromide	0.1722928	4.94E-148
lansoprazole	0.1719626	1.82E-147
thioperamide	0.1714351	1.46E-146
maprotiline	0.1713642	1.93E-146
Prestwick-692	0.171231	3.27E-146
guanadrel	0.1711371	4.73E-146
Prestwick-642	0.1706378	3.36E-145
levobunolol	0.1704225	7.82E-145
pentoxifylline	0.1700828	2.95E-144
pivmecillinam	0.1695888	2.03E-143
naftidrofuryl	0.1685557	1.12E-141
metoprolol	0.1675024	6.53E-140
fludrocortisone	0.1674554	7.83E-140
isoflupredone	0.1667989	9.72E-139
biperiden	0.164233	1.66E-134
tyrphostin AG-1478	0.1603463	3.15E-128
cloxacillin	0.1596394	4.21E-127
furazolidone	0.1595606	5.61E-127
BW-B70C	0.1593004	1.45E-126
captopril	0.1589866	4.55E-126
diazoxide	0.1588517	7.44E-126
myosmine	0.1579789	1.76E-124
diphenylpyraline	0.1574091	1.38E-123
Prestwick-983	0.1571796	3.15E-123
brinzolamide	0.1571332	3.72E-123
homatropine	0.1569938	6.14E-123
co-dergocrine mesilate	0.156284	7.82E-122
acebutolol	0.1562032	1.04E-121
melatonin	0.1553946	1.86E-120
cefamandole	0.154203	1.27E-118
thiamphenicol	0.1533077	2.95E-117
tranexamic acid	0.1529879	9.03E-117
quinpirole	0.1528572	1.42E-116
butamben	0.1516902	8.27E-115
topiramate	0.1514835	1.69E-114
sulmazole	0.1507069	2.47E-113
lidocaine	0.1505691	3.97E-113
ethionamide	0.1501076	1.94E-112
pilocarpine	0.1498635	4.46E-112
nizatidine	0.1496271	1.00E-111
iopromide	0.1490071	8.30E-111
carteolol	0.1487656	1.89E-110

meclofenamic acid	0.1485376	4.09E-110
vigabatrin	0.1475688	1.08E-108
cefoxitin	0.1471559	4.33E-108
isoniazid	0.1465427	3.38E-107
tiaprofenic acid	0.1459064	2.82E-106
indoprofen	0.1449092	7.71E-105
lovastatin	0.1441049	1.09E-103
fursultiamine	0.1427542	9.04E-102
cinchonine	0.142438	2.53E-101
atractyloside	0.1423924	2.93E-101
arecoline	0.1423631	3.22E-101
etynodiol	0.1416024	3.78E-100
metampicillin	0.1404852	1.37E-98
chlortalidone	0.1400423	5.63E-98
nimodipine	0.1399829	6.81E-98
5186223	0.1398496	1.04E-97
minoxidil	0.1391999	8.21E-97
vincamine	0.138998	1.56E-96
ursolic acid	0.1383961	1.04E-95
iloprost	0.1377991	6.80E-95
benzbromarone	0.1369824	8.75E-94
bambuterol	0.1368461	1.34E-93
iopanoic acid	0.1357463	4.05E-92
carbimazole	0.1356898	4.82E-92
chenodeoxycholic acid	0.1348766	5.89E-91
clemastine	0.1346165	1.31E-90
hydrochlorothiazide	0.134598	1.38E-90
etiocholanolone	0.1338822	1.23E-89
5149715	0.1338565	1.33E-89
chloropyrazine	0.1338172	1.50E-89
clofazimine	0.1333376	6.44E-89
quipazine	0.1327695	3.59E-88
iodixanol	0.1326528	5.11E-88
minocycline	0.1325871	6.23E-88
tolazamide	0.1323408	1.31E-87
acetohexamide	0.1303514	4.97E-85
metolazone	0.130234	7.03E-85
bretylium tosilate	0.1299527	1.62E-84
dinoprost	0.1266034	2.80E-80
demecarium bromide	0.1254907	6.75E-79
CP-320650-01	0.1253507	1.01E-78
estradiol	0.1252353	1.40E-78
sodium phenylbutyrate	0.1251596	1.73E-78

isometheptene	0.1249611	3.04E-78
Prestwick-967	0.1239228	5.71E-77
spiradoline	0.1239096	5.93E-77
cimetidine	0.1228958	1.01E-75
cinchonidine	0.1228881	1.04E-75
PNU-0293363	0.1227709	1.44E-75
PF-00539745-00	0.1222466	6.16E-75
vinblastine	0.12194	1.44E-74
estropipate	0.120667	4.78E-73
cromoglicic acid	0.1204181	9.44E-73
cefotiam	0.1197374	6.03E-72
azacyclonol	0.1195646	9.63E-72
ribavirin	0.1194659	1.26E-71
mimosine	0.1185861	1.35E-70
moroxydine	0.1182192	3.62E-70
Prestwick-1103	0.117835	1.01E-69
etilefrine	0.1158144	2.13E-67
ikarugamycin	0.1156785	3.04E-67
josamycin	0.1150487	1.58E-66
fendiline	0.1143404	9.93E-66
2-aminobenzenesulfonamide	0.112282	1.96E-63

Supplementary Table S2. Variables of Calcium Phosphate Treated C57BL/6J Mice After Naringenin or Doxycycline Treatment.

Group	NaCl	$CaPO_4$	$CaPO_4$ + NGN	$CaPO_4$ + Dox
N	11	12	11	11
Weight (g)	21.1±1.92	20.3±1.98	20.1±1.41	21.0±1.89
TC (mM)	3.09±0.37	3.73±0.93	3.53±0.45	3.49±0.64
TG (mM)	1.22±0.32	1.22±0.39	0.97±0.22	1.07±0.32
BP (mmHG)	110.7±4.90	117.9±7.53	120.5±4.43	114.4±4.07

TC, total cholesterol; TG, triglycerides; BP, blood pressure.
Data are means ± SEM.

Supplementary Table S3. Variables of AngII infused ApoE$^{-/-}$ Mice After Naringenin Treatment.

**Group**	**AngII**	**AngII + NGN**
N	12	12
Weight (g)	28.8±1.42	28.9±2.03
TC (mM)	12.3±1.36	8.46±2.45 *
TG (mM)	1.66±0.62	0.69±0.35 *
LDL (mM)	2.08±1.19	1.03±0.53 *
HDL (mM)	1.43±0.14	1.46±0.15
BP (mmHG)	131.0±16.2	136.6±14.27

TC, total cholesterol; TG, triglycerides; BP, blood pressure; LDL, low density lipopeotein; HDL, high density lipoprotein.

Data are means ± SEM.

Supplementary Table S4. Variables of AngII infused ApoE$^{-/-}$ Mice Followed by Naringenin Treatment.

	AngII 28 day		AngII 56 day (with AAA)	
**Group**	**Before**	**After**	**Vehicle gavage**	**NGN gavage**
N	70	70	17	21
Weight (g)	27.8±1,11	27.5±1.78	29.4±1.56	29.7±0.67
TC (mM)	10.5±1.22	11.3±3.87	12.6±1.55	9.67±1.33 *
TG (mM)	1.98±0.55	2.33±0.38	2.39±0.51	1.22±0.47 *
LDL (mM)	1.87±0.98	1.95±0.33	2.06±0.58	1.44±0.38*
HDL (mM)	1.88±0.17	1.97±0.28	1.95±0.1	1.84±0.19
BP (mmHG)	149.6±8.97	144.2±10.16	150.4±5.23	158.45±4.24

TC, total cholesterol; TG, triglycerides; BP, blood pressure; LDL, low density lipopeotein; HDL, high density lipoprotein.

Data are means ± SEM.

Supplementary Table S5. Variables of sham operated or CaPO4-induced TFEB$^{flox/flox}$ Mice or TFEB $^{Mϕ\ ko}$ mice with vehicle or naringenin gavage.

Group	Sham		CaPO4			
	TFEB$^{flox/flox}$	TFEB^{Mϕko}	TFEB$^{flox/flox}$	TFEB^{Mϕko}	TFEB$^{flox/flox}$ + NGN	TFEB^{Mϕko} + NGN
N	6	6	6	6	13	14
Weight (g)	22.6±2.15	24.3±2.43	24.6±2.22	25.1±1.23	24.8±0.62	22.9±2.11
TC (mM)	10.6±1.05	12.0±1.22	10.3±1.16	11.8±0.96	10.3±0.46	11.7±2.25
TG (mM)	1.33±0.55	1.44±0.20	1.45±0.72	1.36±0.14	1.55±0.22	1.69±0.45
BP (mmHG)	115.6±13.05	118.8±8.25	122.6±10.53	114.5±7.43	124.6±9.75	118.0±6.45

TC, total cholesterol; TG, triglycerides; BP, blood pressure
Data are means ± SEM.

Supplementary Table S6. Variables of CaPO4-induced AAA in AAV2-GFP and AAV2-TFEB infected wild type Mice.

**Group**	**AAV2-GFP**	**AAV2-TFEB**
N	9	9
Weight (g)	23.1±1.48	24.5±2.43
TC (mM)	11.4±2.06	12.3±3.22
TG (mM)	1.66±0.32	1.45±0.41
BP (mmHG)	114.2±13.54	108.3±13.3

TC, total cholesterol; TG, triglycerides; BP, blood pressure
Data are means ± SEM.

Supplementary Table S7.The Top 10 Putative NGN-binding Proteins in Macrophage.

Uniprot ID	Gene name	Miss-digested peptide number
Q61207	Prosaposin	15
P97429	Annexin A4	10
P28798	Granulins	7
P48036	Annexin A5	7
Q62192	CD180 antigen	7
O09131	Glutathione S-transferase omega-1	5
Q62426	Cystatin-B	4
P14211	Calreticulin	3
P62259	14-3-3 protein epsilon	1
P11438	LAMP1	1

Supplementary Table S8. Primers used for RT-qPCR.

Primer Name	Forward (5'-3')	Reverse (5'-3')
Tcfeb	AGATGCAGATGCCTAACACG	CATTCCCAGCTCCTTGATCC
TFE3	AGTGGCTACCCCAGCTATCA	CGCCTGCGTCGTTCAATTAG
MiTF	CCCTCTCACCTGTTGGAGTC	TCCGTTTCTTCTGCGCTCAT
ATP6V1A	CCACGTAACAGAGGAAGCGT	AAGGGCATCGAGGACTCTCT
ATP6V1D	CGCAAGTGAAGATTCGAGCG	GGCATTTACACGCCTGTTGG
MMP-2	CAAGTTCCCCGGCGATGTC	TTCTGGTCAAGGTCACCTGTC
MMP-9	CTGGACAGCCAGACACTAAA	CTCGCGGCAAGTCTTCAGAG
18 S	ATTGGAGCTGGAATTACCGC	CGGCTACCACATCCAAGGAA
MMP-3	ACATGGAGACTTTGTCCCTTT	TTGGCTGAGTGGTAGAGTCCC
MMP-12	CTGCTCCCATGAATGACAGTG	AGTTGCTTCTAGCCCAAAGAAC
IRF5	GGGGACAACACCATCTTCAA	CGTTGGAGCAGACCTCGTAGA
IRF3	ACGGCAGGACGACACGAT	TCAGCAGCTAACCGCAACAC
STAT1	TGCCTATGATGTCTCGTTTGC	ATCTGTACGGGATCTTCTTGGA
RBP-J	ACTCGAGGCGTCTGCTTAAC	AGCACTGTTTGATCCCCTCG
KLF6	GGACCAAATTCATTCTAGCTC	AGGCGTCGCCATTACCCTTG
IRF9	TGCTTCCTCCAGAGCCAGAC	CACAAGGCGGCAATCCAG
STAT6	GCTGGACAGACCTACAGACC	GGCGTTGTGGAAAGTCAACATA
STAT3	GTACAAAGGCACTCCATCAG	CCCCATATCGTCTGAAACTCC
KLF4	CTGAACAGCAGGGACTGTCA	GTGTGGGTGGCTGTTCTTTT
KLF2	CGCCTCGGGTTCATTTC	AGCCTATCTTGCCGTCCTTT
ERG2	CGGATCACAGGCAGGAGAGA	GAGGTGGAAGTGGTGGATGG
CMAF	GCCACCTTGTTAAATGCTCCG	GGCAATCCATGAGCCAGACA
GATA3	AGCTGTCTGCGAACACTGAG	GCTCAGAGACGGTTGCTCTT
IRF4	AGGTGACTCTGTGCTTTGGTG	GGGAGCGGTGGTAATCTGG
TSC2	CGGGTGAAGAGAGCCGTATC	TTTCCCCTTCGTGATGGAGC
LAMP1	GATGCCCTAGTGCCCACATT	TGGACCTGCACACTGAAGAC
NPC1	TACGAGTTTGCTCCACGGTC	TCTGGAGCCTCTCTGTGTCA
NPC2	CAAGGACTGCGGCTCTAAGG	GACTGAGTGCCGCTGGTAAA
CTSB	CAGGCTGGACGCAACTTCTA	CCAAATGCCCAACAAGAGCC

Supplementary Table S9. Primers used for Chip-PCR.

Primer Name	Sequence (5'-3')
chip-stat6-ATCACGGGAC-F	CAAGGGACTCCCGGAGAC
chip-stat6-GTCATGTGTG-F	CAGAGGCTGAACAAGGGG
chip-stat6-ACCACATGGT-F	TGGAGAGATGGCTCAGATG
chip-stat6-CCCACATGGT-F	GGACTGGAGAGAGAGCTCAGTG
chip-stat6-ATCACGGGAC-R	AAAACAGCTGGGAGCCCGA
chip-stat6-GTCATGTGTG-R	CACGCATACACACACACATAC
chip-stat6-ACCACATGGT-R	TGAGTACACTGTCACTGTCTT
chip-stat6-CCCACATGGT-R	GTGCTCTCAGAGGCTAGAGG
chip-IRF4-GTCACATGCT-F	TAAACGCTCAGAGAGACAAG
chip-IRF4-GTCACATGCT-R	AACTACTAACTGCTCATGGC
chip-IRF4-GGCACAAGGC-F	TTTGTCTGAAGTGGGACAGCC
chip-IRF4-GGCACAAGGC-R	TGCAGGGCGGGCGCTTCGGCCC
chip-IRF4-CCCACGAGGC-F	TAAAGTAAAACCTTCAG
chip-IRF4-CCCACGAGGC-R	ATCCCTGAGTACTCAGGTAGT
chip-IRF4-AACATGTGAA-F	GTGGCCAAAATCAGATGT
chip-IRF4-AACATGTGAA-R	TGTATCATTTTAGATTACC
chip-IRF4-GACACGTGTG-F	AAACTTCTGAAAACAGAG
chip-IRF4-GACACGTGTG-R	AGGACAGAAGCAGCCAACCC
chip-IRF4-ACCATGTGAA-F	CAACGAGCGAGCGAGTA
chip-IRF4-ACCATGTGAA-R	CCTCAGCCAATCTACATCC
chip-IRF4-CTCACATGTT-F	CTCAATATGTCGTGTG
chip-IRF4-CTCACATGTT-R	TGTCGCCATTTCTTACCA
chip-GATA3-CACACCTGAC-F	TGGAGCCTAGGCTGGTCT
chip-GATA3-CACACCTGAC-R	CCGAGACAGCAGAGCTG
chip-GATA3-AACTCCTGAC-F	TAATGGGGTCTCACA
chip-GATA3-AACTCCTGAC-R	TGTAGCCTTTGAAATGGTTT
chip-GATA3-GCCAGGTGCT-F	TCCTCAGCTGAGGTTTCCAG
chip-GATA3-GCCAGGTGCT-R	ATGAATATCCAGCCGGCTTC

Supplementary Table S10. Primers used for subcloning of pGL3-GATA3/IRF4/STAT6-luc plasmids.

Primer Name	Sequence (5'-3')
vectorF	TAGAGGGTATATAATGGAAGCTCGAATTCCAGCTCGAGATCTG CGATCTAAGT
vectorRSTAT6	ACTGAGCTCTCTCTCCAGTCCTATCGATAGAGAAAT
vectorRIRF4	ACACCACACGACATATTGAGTATCGATAGAGAAAT
vectorRGATA3	CAGAACACACAGGGGCTAACTATCGATAGAGAAAT
promoter STAT6F	ATTTCTCTATCGATAGGACTGGAGAGAGAGCTCAGT
promoter STAT6R	CTTCCATTATATACCCTCTACACGCACTCGTCTCCGGG
promoter IRF4F	ATTTCTCTATCGATACTCAATATGTCGTGTGGTGT
promoter IRF4R	CTTCCATTATATACCCTCTACCTCACACTCCTCCTTCTGC
promoter GATA3F	ATTTCTCTATCGATAGTTAGCCCCTGTGTGTTCTG
promoter GATA3R	CTTCCATTATATACCCTCTAGGGAGCAGAATGTCACTCT
